# Supplementary material for: Large-scale analysis of small molecule-RNA interactions using multiplexed RNA structure libraries
Source: Commun Chem. 2024 May 1;7:98. doi: 10.1038/s42004-024-01181-8 (PMC11865577; doi:10.1038/s42004-024-01181-8)

Supplementary Information

Large-scale Analysis of Small Molecule-RNA Interactions using Multiplexed RNA Structure Libraries

Ryosuke Nagasawa,[a,b,‡] Kazumitsu Onizuka,[a,b,c,‡,*] Kaoru R. Komatsu,[d,‡] Emi Miyashita,[d] Hirotaka Murase,[a] Kanna Ojima,[a,b] Shunya Ishikawa,[a,b] Mamiko Ozawa,[a] Hirohide Saito,[d,*] and Fumi Nagatsugi [a,b,*]

[a] Institute of Multidisciplinary Research for Advanced Materials, Tohoku University, Sendai, Miyagi 980-8577, Japan.
[b] Department of Chemistry, Graduate School of Science, Tohoku University, Sendai, Miyagi 980-8578, Japan.
[c] Division for the Establishment of Frontier Sciences of Organization for Advanced Studies, Tohoku University, Sendai, Miyagi 980-8577, Japan.
[d] Center for iPS Cell Research and Application (CiRA), Kyoto University, Kyoto, 606-8507, Japan.

Table of Contents

Supplementary Methods: Experimental procedures (Schemes S1-S5) S2-S13
Supplementary Figures:
Figure S1. HPLC profiles of biotin conjugation of G-clamp-N_3 S14
Figure S2. HPLC profiles of biotin conjugation of TO derivatives S14
Figure S3. Evaluation of the relationship between the number of multiple Gs and the affinity ranking of G-clamp S15
Figure S4. Fluorescence titrations to determine K_D values of G-clamp-N_3 S16-S21
Figure S5. Minimum free energy structures calculated by RNAfold S22
Table S1. The correlation between Z-score and K_D value for G-clamp S23
Figure S6. Surface plasmon resonance (SPR) analysis for G-clamp-N_3 S24
Figure S7. Molecular modeling of the complex structure between hsa-mir-4520-1 and G-clamp–N_3 S25
Figure S8. Molecular modeling structure of mir-4520-1 and mir-548ba S25
Figure S9. Kernel density estimation of TO derivatives and G-clamp Z-scores S26
Figure S10. Fluorescence titrations to compare K_D values for a hsa-mir-4437 loop with mir-4437-mut S27
Figure S11. Fluorescence titrations to determine K_D values of TO-N_3 S28
Figure S12. Fluorescence titrations to determine K_D values of TO-3-N_3 S29
Figure S13. Fluorescence titrations to determine K_D values of TO-PRO-1 S30
Figure S14. Fluorescence titrations to determine K_D values of TO-PRO-3 S31
Figure S15. Minimum free energy structures for the assay with TO derivatives S32
Table S2. The correlation between Z-scores and KD values for TO-N_3 and TO-3-N_3 RNA binding
Table S3. The correlation between Z-scores and KD values for TO-PRO-1 RNA binding S33
Table S4. The correlation between Z-scores and KD values for TO-PRO-3 RNA binding S34
Figure S16. Normalized fluorescences in FID assay S35
Figure S17. Fluorescence titrations to determine K_D values of baicalein S36
Figure S18. Fluorescence titrations to determine K_D values of myricetin S36
Figure S19. Fluorescence titrations to determine K_D values of chelerythrine chloride S37
Figure S20. The precisions of FID assay S37
Figure S21. Fluorescence titrations to determine K_D values of AS 602801 S38
NMR data S39-S50

Supplementary Methods

Experimental Procedures

Material and methods

General chemicals were purchased from FUJIFILM Wako Pure Chemical, the Tokyo Chemical Industry, Kanto Chemical or Aldrich. Target RNAs were purchased from JBioS (Japan). ^{1}H NMR spectra (400, 500, and 600 MHz) were recorded using Bruker AVANCE III 400, 500, and 600 spectrometers, respectively. ^{13}C NMR spectra (125 and 151 MHz) were recorded using Bruker AVANCE III 500 and 600 spectrometers, respectively. High-resolution electrospray mass analysis was performed using a Bruker MicrOTOF-Q II. HPLC purification was performed with a JASCO HPLC System (PU-2089Plus, UV-2075Plus, and CO-2065Plus) using a reverse-phase C_{18} column (COSMOSIL 5C_{18}-AR-II, Nacalai Tesque, 4.6×250 mm for analysis or 10×250 mm for ligand purification).

Synthesis of G-clamp-N_3

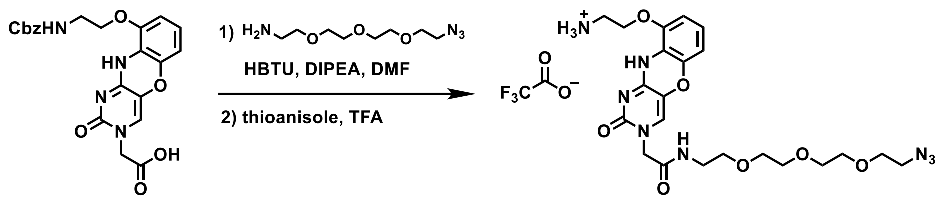

G-clamp-COOH G-clamp-N_3 Scheme S1

To a solution of G-clamp-COOH (100 mg, 0.22 mmol) in DMF (3.4 mL), HBTU (126 mg, 0.33 mmol), DIPEA (224 µL, 1.32 mmol), and 11-azido-3,6,9-trioxaundecan-1-amine (65 µL, 0.33 mmol) were added, and the reaction mixture was stirred at room temperature overnight. The reaction mixture was diluted with DCM, and then washed with saturated aqueous $NaHCO_3$ and brine. The organic layer was separated and dried over Na_2SO_4, filtered, and evaporated. The residue was purified by column chromatography ($CHCl_3$: MeOH = 30:1) to afford Cbz-protected G-clamp-N_3 as a yellow solid (122 mg, 85%).

To a solution of the above compound (10 mg, 0.015 mmol) in TFA (200 µL), thioanisole (43 µL, 0.38 mmol) was added, and the mixture was stirred at room temperature overnight. TFA was completely removed in vacuo, and the resulting residue was dissolved in MeOH, washed with Hexane and evaporated. The crude product was purified by reverse phased HPLC with C-18 column (Nacalai Tesque: COSMOSIL 5C_{18}-AR-II, 10 × 250 mm) by a liner gradient of 0-40%/30 min acetonitrile in 0.1% TFA buffer at a flow rate of 4 mL/min at 40 ^{o}C, and monitored by UV detection at λ = 254 nm, to afford G-clamp-N_3 as a yellow solid (11.6 µmol, 74%). The concentration of G-clamp-N_3 was determined by quantitative ^{1}H-NMR using maleic acid as an internal standard. ^{1}H-NMR (400 MHz, DMSO-d_6) δ 3.22-3.27 (m, 4H), 3.38-3.44 (m, 4H), 3.50-3.57 (m, 8H), 3.59 (t, J = 4.8 Hz, 2H), 4.16 (t, J = 4.8 Hz, 2H), 4.26 (s, 2H), 6.51 (d, J = 8.0 Hz, 1H), 6.67 (d, J = 8.0 Hz, 1H), 6.84 (t, J = 8.0 Hz, 1H), 7.45 (s, 1H), 8.03 (brs, 3H), 8.20 (t, J = 5.4 Hz, 1H), 9.82 (brs, 1H). ^{13}C-NMR (151 MHz, DMSO-d_6) δ 42.3, 50.2, 50.8, 65.1, 69.2, 69.4, 69.79, 69.87, 69.95, 69.98 107.2, 108.7, 116.0, 118.1, 123.4, 125.7, 130.0, 142.5, 145.7, 154.3, 158.2, 167.4. ESI-HRMS (m/z): $[M+H]^+$ calcd for $C_{22}H_{31}N_8O_7^+$, 519.2310; found 519.2334.

Synthesis of TO-N_3

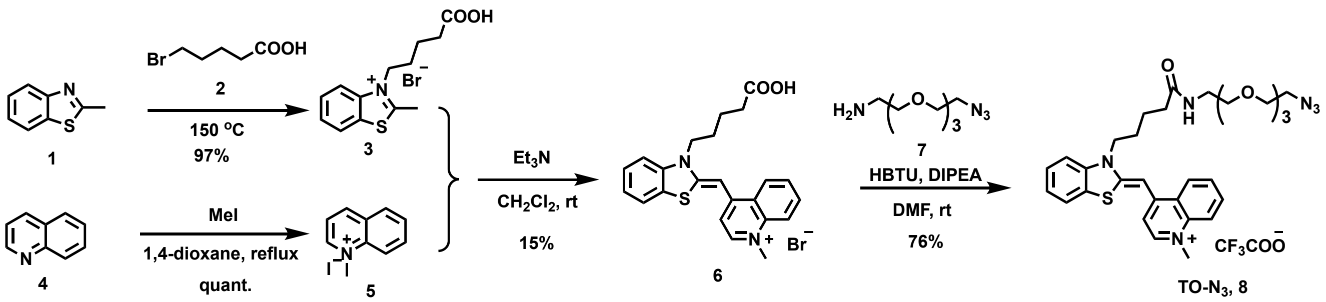

Scheme S2

Synthesis of compound 3

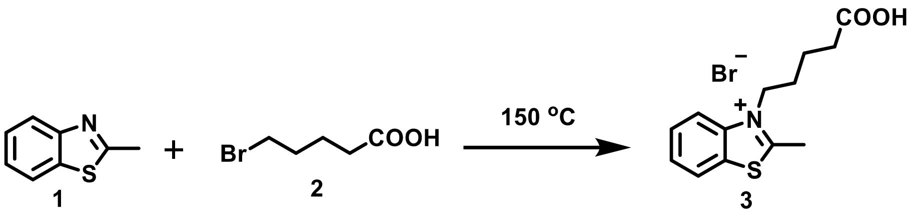

Compound **1** (2.98 g, 20.0 mmol) and compound **2** (5.41 g, 29.9 mmol) were mixed neat at 150 °C for 23 h. After the mixture was cooled to room temperature, Et_2O (10 mL) was added. The mixture was washed with Et_2O (30 mL) to provide compound **3** (7.61 g, 97% yield) as a brown solid. The yield was calculated by ^{1}H-NMR analysis because the crude contained byproduct which is HBr salt of compound **1** (compound **3**: HBr salt of compound **1** = 1 : 0.28). ESI-HRMS (*m/z*): $[M-Br^-]^+$ calcd for $C_{13}H_{16}NO_2S^+$, 250.0896, found 250.0889.

Synthesis of compound 5

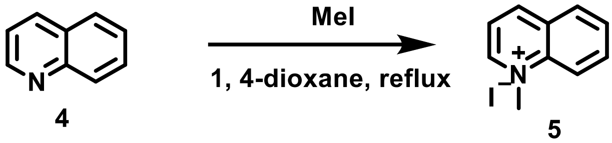

To a solution of quinoline **4** (1.13 g, 8.72 mmol) in 1,4-dioxane (15 mL), MeI (2.3 g, 16 mmol) was added dropwise and refluxed for 22 h. The mixture was cooled to room temperature before filtration. The mixture was washed with Et_2O (3 mL×3) and hexane (3 mL×3) to afford compound **5** (2.27 g, quant) as a yellow solid. ^{1}H-NMR (400 MHz, DMSO-*d₆*) δ 4.64 (s, 3H), 8.08 (dt, *J* = 1.2, 6.0 Hz, 1H), 8.17 (dd, *J* = 5.6, 8.4 Hz, 1H), 8.30 (dt, *J* = 1.6, 7.2 Hz, 1H), 8.48 (dd, *J* = 1.2, 8.0 Hz, 1H), 8.52 (dd, *J* = 0.8, 8.8 Hz, 1H), 9.28 (d, *J* = 8.4 Hz, 1H), 9.50 (dd, *J* = 0.8, 5.8Hz, 1H).

Synthesis of compound 6

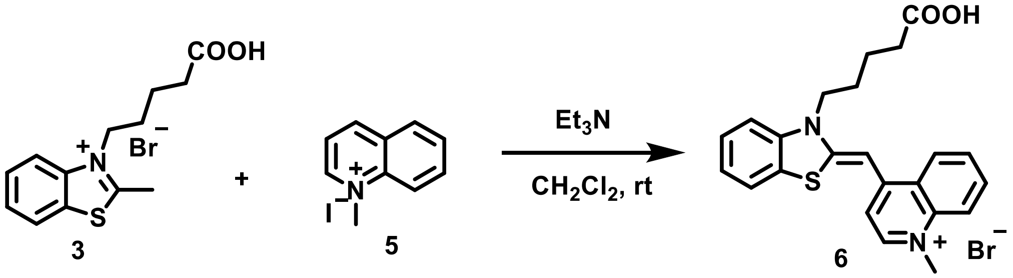

To a solution of compound **3** (0.195 g, 0.590 mmol), compound **5** (0.162 g, 0.635 mmol) in CH_2Cl_2 (5.6 mL) and Et_3N (0.62 g, 6.1 mmol) were added and the mixture was stirred at room temperature for 18 h. The mixture was evaporated under reduced pressure, filtrated with acetone (20 mL) and dried under reduced pressure. Then, the mixture was filtrated with H_2O (10 mL) and dried under reduced pressure. The mixture was dissolved in MeOH (10 mL) and solid residues were filtrated out. The filtrate was evaporated under reduced pressure. The residue was purified by reprecipitation with MeOH (2 mL) and Et_2O (20 mL) and filtrated to afford compound **6** (42.7 mg, 15% yield) as a red solid. ^{1}H-NMR (400 MHz, DMSO-*d₆*) δ 1.69 (m, 2H), 1.77 (m, 2H), 2.28 (t, *J* = 6.8 Hz, 2H), 4.13 (s, 3H), 4.56 (t, *J* = 7.6 Hz, 2H), 6.89 (s, 1H), 7.32 (d, *J* = 6.8 Hz, 1H), 7.39 (t, *J* = 7.6 Hz, 1H), 7.58 (t, *J* = 8.0 Hz, 1H), 7.72 (d, *J* = 8.4 Hz, 1H), 7.76 (td, *J* = 1.6, 7.2 Hz, 1H), 7.97 (m, 3H), 8.56 (d, *J* = 7.2 Hz, 1H), 8.76 (d, *J* = 8.4 Hz, 1H).

Synthesis of compound 8

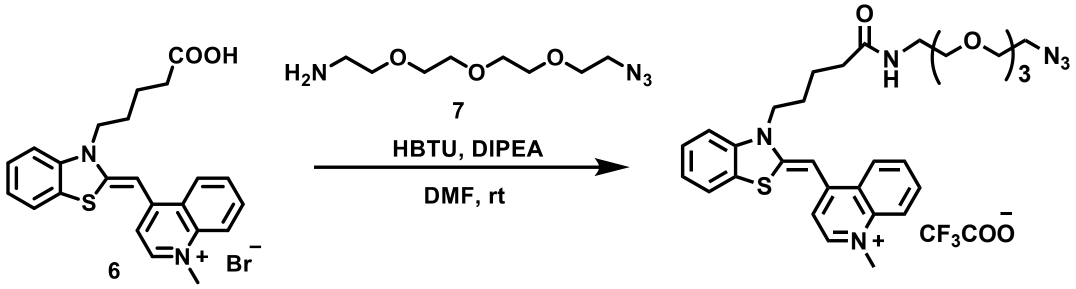

TO-N$_3$, 8

To a solution of compound **6** (9.7 mg, 21 μmol) in DMF (0.40 mL), DIPEA (16 mg, 0.12 mmol) and HBTU (14 mg, 36 μmol) were added first, then compound **7** (11 mg, 50 μmol) was added, and the mixture was stirred at room temperature for 20 h. The mixture was evaporated under reduced pressure and filtrated with DMSO (1.2 mL). The filtrate was purified by reverse phased HPLC to provide compound **8** (10.7 μmol, 76% yield) as a red solid. The concentration of **TO-N$_3$** was determined by quantitative ^{1}H-NMR using maleic acid as an internal standard. HPLC conditions: A: 0.1% TFA in distilled water, B: 0.1% TFA in MeCN; B: 0%→60% (～20 min) → 100% (～25 min). Flow rate = 4 mL/min; Temp. = 28.8 °C; UV = 254 nm, C-18 column (Nacalai tesque : COSMOSIL 5C18-AR-II, 10×250 mm). ^{1}H-NMR (600 MHz, DMSO-*d$_6$*) *δ* 1.72 (m, 2H), 1.79 (m, 2H), 2.18 (t, *J* = 7.2 Hz, 2H), 3.16 (m, 2H), 3.30-3.57 (m, 14H),4.19 (s, 3H), 4.63 (t, *J* = 7.8 Hz, 2H), 6.96 (s, 1H), 7.42 (dt, *J* = 1.2, 6.6 Hz, 2H), 7.61 (tt, *J* = 1.2, 5.2 Hz, 1H), 7.80 (m, 2H), 7.88 (t, *J* = 5.4 Hz, 1H), 8.06 (m, 3H), 8.63 (d, *J* = 7.2 Hz, 1H), 8.81 (d, *J* = 7.8 Hz, 1H). ^{13}C-NMR (151 MHz, DMSO-*d$_6$*) *δ* 22.3, 26.3, 34.6, 38.4, 42.4, 45.5, 49.9, 69.1, 69.2, 69.5, 69.6, 69.7, 69.7, 87.5, 108.0, 112.9, 118.3, 122.9, 123.8, 124.1, 124.5, 125.6, 127.0, 128.2, 133.2, 138.1, 140.0, 145.1, 148.9, 159.3, 171.9. ESI-HRMS (*m/z*): $[M\text{-}CF_3COO^-]^+$ calcd for $C_{31}H_{39}N_6O_4S^+$, 591.2748, found 591.2748.

Synthesis of TO-3-N$_3$

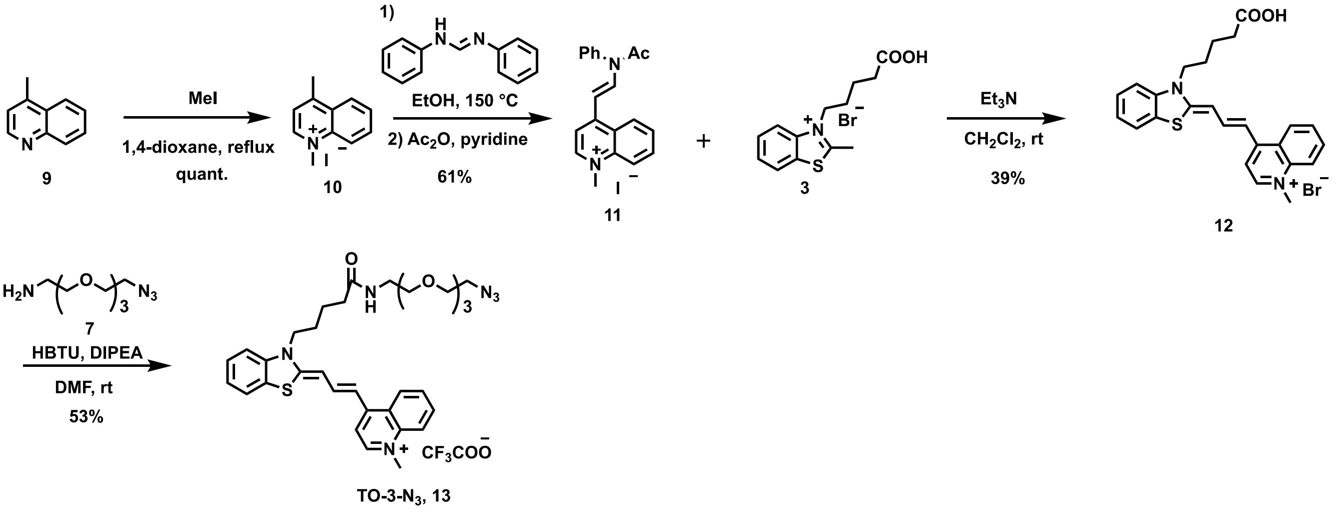

Scheme S3

Synthesis of compound 10

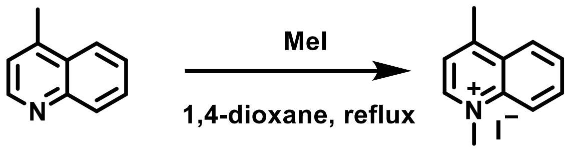

9 10

To a solution of 4-methylquinoline **9** (1.08 g, 7.58 mmol) in 1,4-dioxane (13 mL), MeI (2.1 g, 14.4 mmol) was added dropwise and refluxed for 19 h. The mixture was cooled to room temperature before filtration with Et_2O (5 mL). The mixture was washed with Et_2O (3 mL×3) and hexane (3 mL×3) to provide compound **10** (2.20 g, quant) as a yellow solid. ^{1}H-NMR (400 MHz, DMSO-*d*$_6$) δ 3.00 (s, 3H), 4.57 (s, 3H), 8.06 (m, 2H), 8.27 (dt, *J* = 2.0, 6.8 Hz, 1H), 8.48 (d, *J* = 8.8 Hz, 1H), 8.53 (dd, *J* = 1.0, 8.8 Hz, 1H), 9.34 (d, *J* = 6.0 Hz, 1H).

Synthesis of compound 11

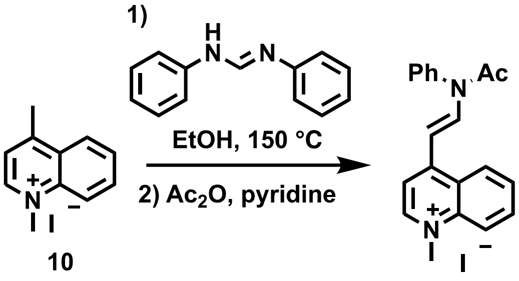

10 11

To a solution of compound **10** (0.518 g, 1.82 mmol) in EtOH (3.0 mL), diphenylformamidine (0.694 g, 3.54 mmol) was added and the mixture was stirred at 150 °C for 40 min. The mixture was cooled to room temperature and filtrated with EtOH (20 mL) to afford enamine intermediate (1.00 g). Pyridine (5.6 mL) and Ac_2O (1.8 g, 18 mmol) were added to the intermediate and the mixture was stirred at room temperature for 18 h. The mixture was diluted with AcOEt (28 mL), filtrated with AcOEt (30 mL) and dried under reduced pressure. To the residue, CH_2Cl_2 (10 mL) was added and stirred at room temperature for 40 min to be dissolved. Solid residues in the solution were filtrated out and the filtrate was evaporated under reduced pressure. The residue was purified by reprecipitation with CH_2Cl_2 (0.5 mL) and Et_2O (8.4 mL) and filtration with Et_2O (40 mL) to afford compound **11** (0.476 g, 61% yield) as a pale green solid. ^{1}H-NMR (400 MHz, DMSO-*d*$_6$) δ 2.07 (s, 3H), 4.46 (s, 3H), 6.04 (d, *J* = 14 Hz, 1H), 7.73 (d, *J* = 8.4 Hz, 2H), 7.69 (m, 3H), 7.89 (m, 2H), 8.17 (dt, *J* = 2.0, 6.8 Hz, 1H), 8.35 (d, *J* = 8.8 Hz, 1H), 8.36 (d, *J* = 6.8 Hz, 1H), 8.90 (d, *J* = 13.6 Hz, 1H), 9.14 (d, *J* = 6.8 Hz, 1H).

Synthesis of compound 12

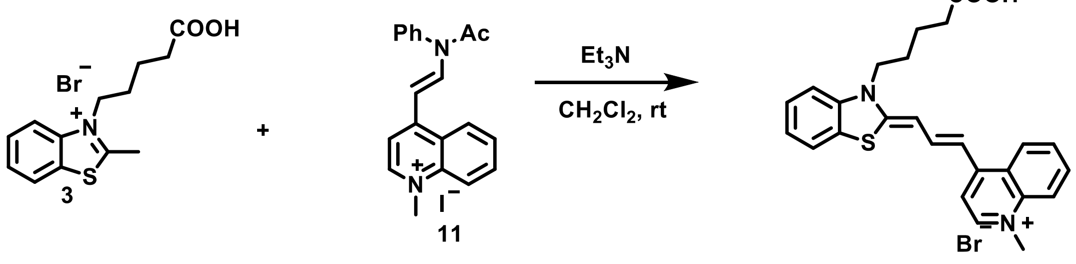

3 11 12

To a solution of compound **3** (0.103 g, 0.313 mmol), compound **11** (0.132 g, 0.306 mmol) in CH_2Cl_2 (1.0 mL) and Et_3N (147 mg, 6.1 mmol) were added and stirred at room temperature for 16 h. The mixture was evaporated under reduced pressure, filtrated with CH_2Cl_2 (6 mL), washed with acetone (20 mL) and dried under reduced pressure to provide compound **12** (61.6 mg, 39% yield) as a green-purple solid. ^{1}H-NMR(400 MHz, DMSO-*d*$_6$) δ1.64 (m, 2H), 1.72 (m, 2H), 2.20 (t, *J* = 6.4 Hz, 2H), 4.11 (s, 3H), 4.21 (t, *J* = 7.2 Hz, 2H), 6.51 (d, *J* = 12 Hz, 1H), 7.11 (d, *J* = 13.6 Hz, 1H), 7.27 (t, *J* = 7.6 Hz, 1H), 7.42 (t, *J* = 8.0 Hz, 1H), 7.55 (d, *J*

= 8.4 Hz, 1H), 7.71 (m, 1H), 7.82 (t, J = 7.2, 2H), 7.96 (m, 2H), 8.11 (t, J = 12.4 Hz, 1H), 8.36 (d, J = 6.8 Hz, 1H), 8.46 (d, J = 8.8 Hz, 1H).

Synthesis of compound 13

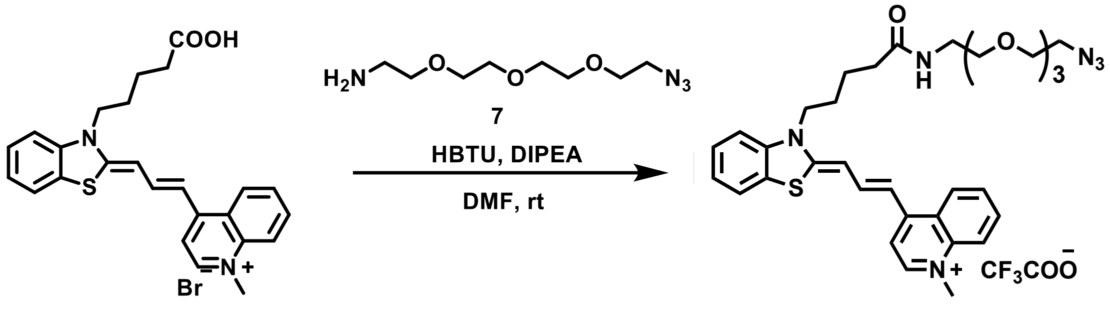

To a solution of compound **12** (10.2 mg, 20.5 μmol) in DMF (0.60 mL), DIPEA (16 mg, 0.13 mmol) and HBTU (12.3 mg, 32.4 μmol) were added first, then compound **7** (11 mg, 50 μmol) was added and stirred at room temperature for 21 h. Then, HBTU (6.5 mg, 17.1 μmol) and compound **7** (11 mg, 50 μmol) were added to the mixture and the mixture was stirred for further 21 h. The mixture was evaporated under reduced pressure and filtrated with DMSO (1.2 mL). The filtrate was purified by reverse phased HPLC to provide compound **13** (10.9 μmol, 53% yield) as a blue solid. The concentration of **TO-3-N_3** was determined by quantitative ^{1}H-NMR using maleic acid as an internal standard. HPLC conditions: A: 0.1% TFA in distilled water, B: 0.1% TFA in MeCN; B: 0% → 60% (～20 min)→100%(～25 min). Flow rate = 4 mL/ min; Temp. = 26.6 °C; UV = 254 nm, C-18 column (Nacalai Tesque: COSMOSIL 5C18-AR-II, 10×250 mm). ^{1}H-NMR (600 MHz, DMSO-d_6) δ 1.66 (m, 4H), 2.18 (t, J = 4.4 Hz, 2H), 3.18 (m, 2H), 3.35-3.57 (m, 14H), 4.13 (s, 3H), 4.23 (t, J = 7.2 Hz, 2H), 6.50 (d, J = 12.6 Hz, 1H), 7.13 (d, J = 13.2 Hz, 1H), 7.30 (dt, J = 0.6, 7.5 Hz, 1H), 7.48 (dt, J = 0.6, 7.2 Hz, 1H), 7.58 (d, J = 8.4 Hz, 1H), 7.73 (dt, J = 1.8, 6.6 Hz, 1H), 7.86 (d, J = 7.8, 2H), 7.92 (t, J = 5.4 Hz, 1H), 7.99 (m, 2H), 8.14 (t, J = 12.6 Hz, 1H), 8.41 (d, J = 6.6 Hz, 1H), 8.47 (d, J = 8.4 Hz, 1H). ^{13}C-NMR (151 MHz, DMSO-d_6) δ 22.4, 26.6, 34.8, 38.5, 42.3, 45.4, 50.1, 69.2, 69.3, 69.6, 69.7, 69.8, 69.8, 98.4, 109.5, 109.7, 112.5, 118.2, 122.7, 124.1, 124.2, 124.8, 125.0, 126.9, 127.7, 133.5, 139.0, 141.5, 143.4, 143.9, 150.6, 160.8, 171.9. ESI-HRMS (m/z): $[M-CF_3COO^-]^+$ calcd for $C_{33}H_{41}N_6O_4S^+$, 617.2905, found 617.2907.

Synthesis of TO-N$_3$-2

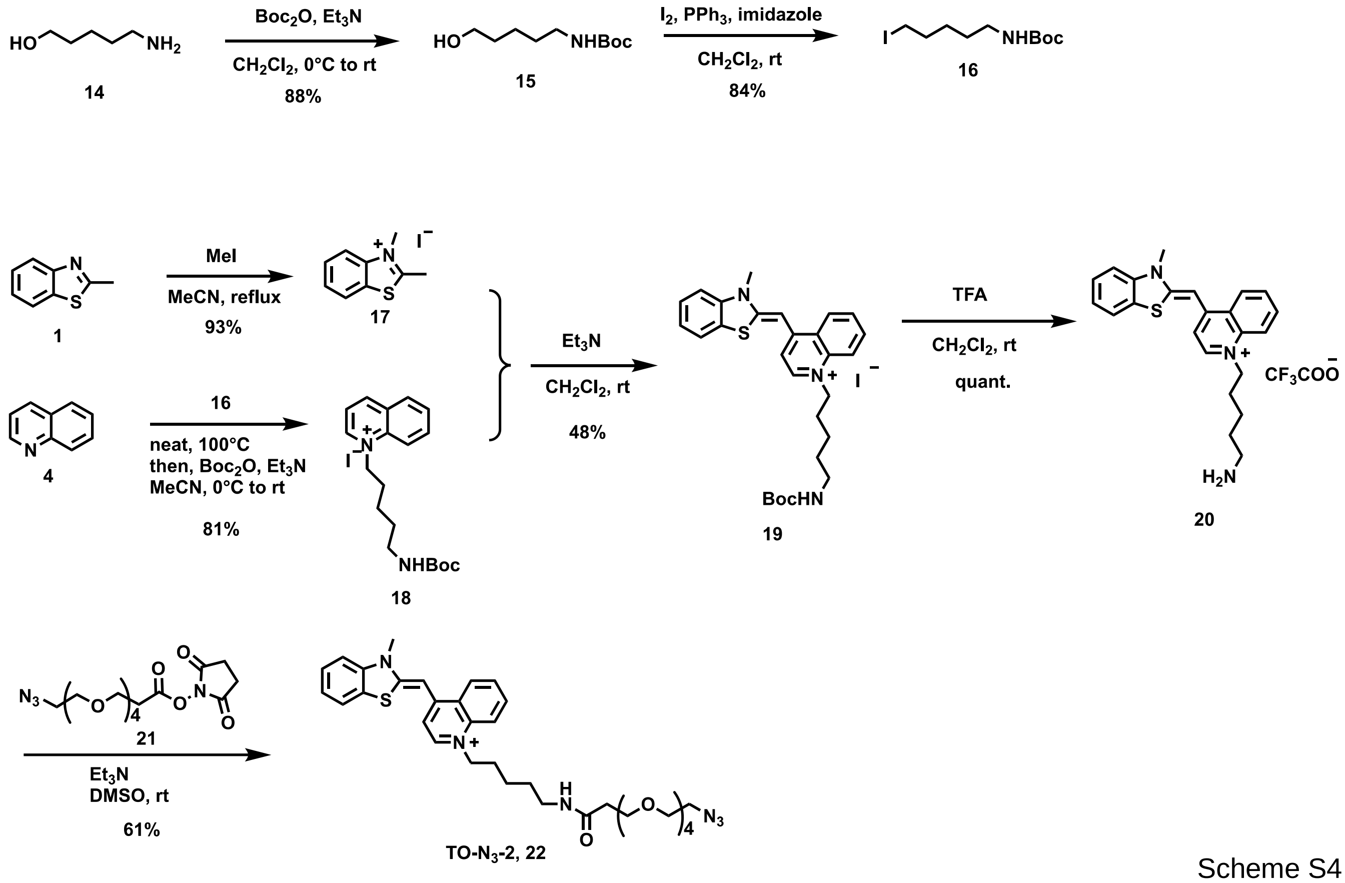

Scheme S4

Synthesis of compound 15

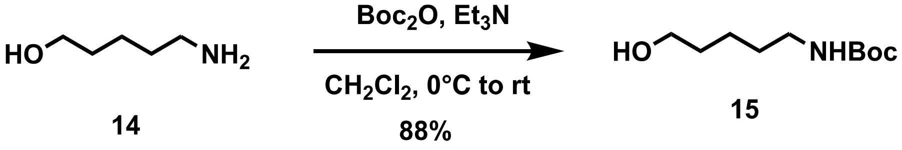

To a solution of 5-amino-1-pentanol **14** (1.01 g, 9.75 mmol) in CH_2Cl_2 (19 mL), Et_3N (1.1 g, 11 mmol) was added and cooled to 0 °C, then Boc_2O (2.4 g, 11 mmol) was added and stirred. After 6 min, the temperature of the mixture was raised to room temperature and stirred for 20 h. H_2O (50 mL) and $CHCl_3$ (50 mL) were added to the mixture, the organic and aqueous layers were separated, then the aqueous layer was extracted with $CHCl_3$ (30 mL×3). The organic layers were combined and dried over Na_2SO_4. The solution was filtered and concentrated under reduced pressure. The residue (3.48 g) was purified by column chromatography (hexane/AcOEt = 2/1 to 1/1 to 1/2) to afford compound **15** as a colourless oil (1.74 g, 88% yield). ^{1}H-NMR (500 MHz, $CDCl_3$) δ 1.39 (m, 2H), 1.43 (s, 9H), 1.51 (quintet, J = 7.3 Hz, 2H), 1.59 (quintet, J = 7.0 Hz, 2H), 3.12 (q, J = 6.3 Hz, 2H), 3.64 (t, J = 6.5 Hz, 2H), 4.55 (brs, 1H). ESI-HRMS (m/z): $[M+H]^+$ calcd for $C_{10}H_{22}NO_3^+$, 204.1594, found 204.1578.

Synthesis of compound 16

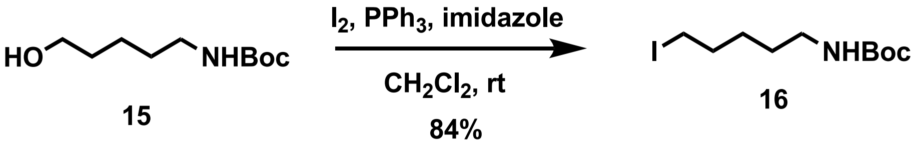

To a solution of I_2 (2.62 g, 10.3 mmol) in CH_2Cl_2 (7.0 mL), PPh_3 (3.38 g, 12.9 mmol) and imidazole (0.877 g, 12.9 mmol) were added at 0 °C. A solution of compound **15** (1.74 g, 8.56 mmol) in CH_2Cl_2 (10 mL) was added

to the mixture and then warmed to room temperature and stirred for 15 h. Sat. $Na_2S_2O_3$ aq. (50 mL) and CH_2Cl_2 (50 mL) were added to the mixture, the organic and aqueous layers were separated, then the aqueous layer was extracted with CH_2Cl_2 (50 mL). The organic layers were combined and washed with sat. $Na_2S_2O_3$ aq. (50 mL) and brine (50 mL), then dried over Na_2SO_4. The solution was filtered and evaporated under reduced pressure. The residue (6.41 g) was purified by column chromatography (hexane/Et_2O = 8/1 to 4/1) to afford compound **16** as a colourless oil (2.26 g, 84% yield). ^{1}H-NMR (400 MHz, $CDCl_3$) δ 1.41 (m, 2H), 1.44 (s, 9H), 1.49 (m, 2H), 1.84 (quintet, J = 7.2 Hz, 2H), 3.12 (q, J = 6.3 Hz, 2H), 3.18 (t, J = 7.0 Hz, 2H), 4.52 (brs, 1H). ESI-HRMS (*m*/*z*): $[M+Na]^+$ calcd for $C_{10}H_{20}INNaO_2^+$, 336.0421, found 336.0423.

Synthesis of compound 17

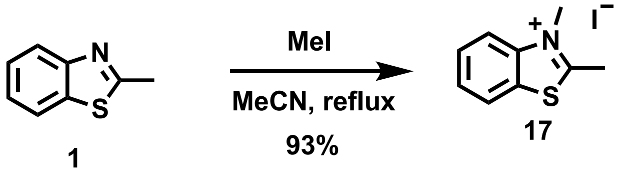

To a solution of 2-methylbenzothiazole **1** (0.504 g, 3.38 mmol) in MeCN (12 mL), MeI (1.0 g, 7.3 mmol) was added and the mixture was refluxed for 23 h. Further MeI (0.91 g, 6.4 mmol) was added to the mixture and refluxed for 17 h. After the mixture was cooled to room temperature, the solid was filtrated and washed with AcOEt (30 mL) to afford compound **17** as a white solid (0.914 g, 93% yield). ^{1}H-NMR (500 MHz,DMSO-d_6) δ 3.17 (s, 3H), 4.20 (s, 3H), 7.81 (dt, J = 0.8, 7.8 Hz, 1H), 7.90 (dt, J = 1.2, 8.0 Hz, 2H), 8.29 (d, J = 8.5 Hz, 1H), 8.44 (d, J = 7.5 Hz, 1H). ESI-HRMS (*m*/*z*): $[M-I]^+$ calcd for $C_9H_{10}NS^+$, 164.0528, found 164.0534.

Synthesis of compound 18

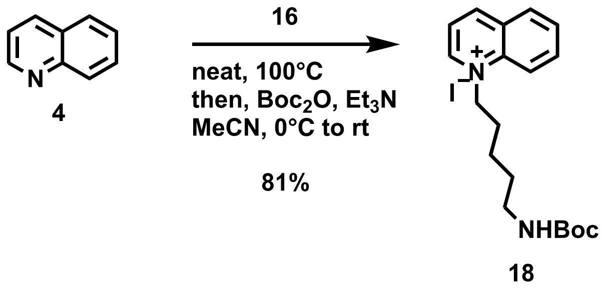

Compound **16** (2.10 g, 6.71 mmol) and quinoline **4** (0.75 g, 5.8 mmol) were added to a round flask, and the mixture was stirred for 4 h at 100 °C. After the mixture was cooled to room temperature, MeCN (13 mL) and Et_3N (0.58 g, 5.7 mmol) were added to the mixture, and it was cooled to 0 °C. Boc_2O (1.2 g, 5.7 mmol) was added to the stirred solution at 0 °C. After 10 min, the temperature of the solution was raised to room temperature and stirred for 17 h. The mixture was evaporated under reduced pressure and filtrated with Et_2O (30 mL). The solid was dissolved in DCM/MeOH = 9/1 (50 mL). The DCM/MeOH washes were evaporated and dried to afford compound **18** as a red solid (2.44 g, 81% yield). The yield was determined by ^{1}H-NMR analysis because T.M. contained byproduct. The compound **18** was used in the next reaction without further purification. ESI-HRMS (*m*/*z*): $[M-I]^+$ calcd for $C_{19}H_{27}N_2O_2^+$, 315.2067, found 315.2075.

Synthesis of compound 19

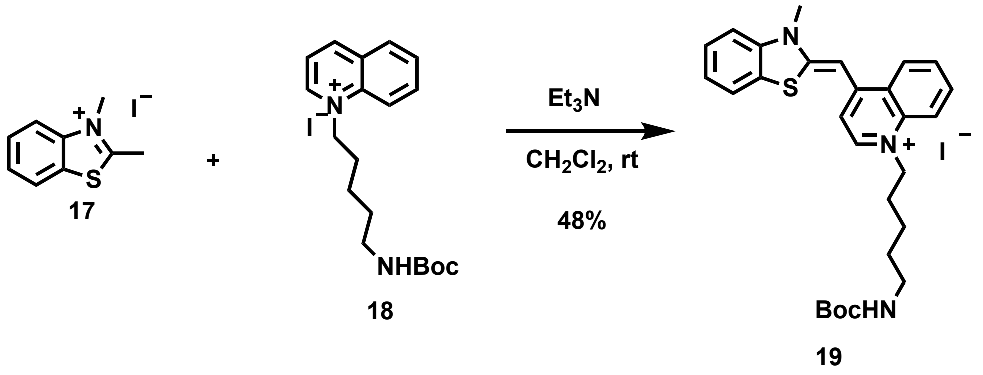

To a solution of compound **18** (2.60 mmol) and compound **17** (0.897 g, 3.08 mmol) in CH_2Cl_2 (29 mL), Et_3N (3.2 g, 31 mmol) was added and stirred for 17 h at room temperature. The mixture was evaporated under reduced pressure, filtrated and washed with Et_2O (60 mL) and H_2O (30 mL). The solid was dissolved in DCM/MeOH = 20/1 (100 mL) and filtrated. The DCM/MeOH washes were evaporated and dried to afford compound **19** as a red solid (1.48 mmol, 48% yield). The yield was determined by ^{1}H-NMR analysis because T.M. contained by-product. The compound **19** was used in the next reaction without further purification. ESI-HRMS (*m/z*): $[M-I]^+$ calcd for $C_{28}H_{34}N_3O_2S^+$, 476.2366, found 476.2369.

Synthesis of compound 20

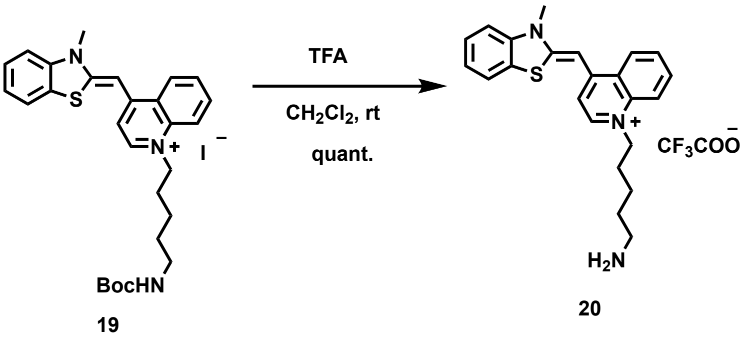

To a solution of compound **19** (48.5 μmol) in CH_2Cl_2 (67 μL), TFA (0.60 mL) was added and the mixture was stirred for 3.5 h at room temperature. The mixture was evaporated under reduced pressure to afford compound **20** (quant) as a red solid. The compound **20** was used in the next reaction without further purification.

Synthesis of compound 22

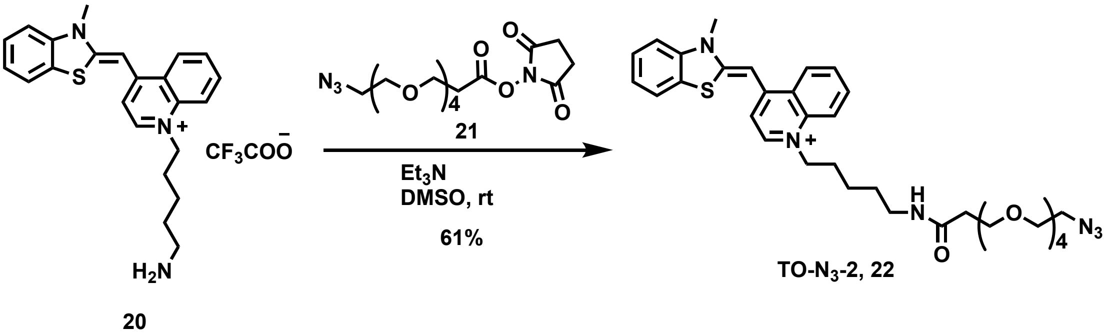

To a solution of compound **20** (15.3 μmol) in DMSO (925 μL), a solution of Azido-PEG$_4$-NHS-Ester **21** (6.54 mg, 16.8 μmol) in DMSO (17 μL) and Et_3N (1.76 mg, 17.4 μmol) were added and stirred for 3 d at room temperature. DMSO (200 μL) was added to the mixture, and it was filtrated and purified by reverse phased HPLC to afford compound **22** as a red solid (61% yield). The concentration of **TO-N$_3$-2** was determined by quantitative ^{1}H-NMR using maleic acid as an internal standard. HPLC conditions: A: 0.1% TFA in distilled

water, B: 0.1% TFA in MeCN; B: 0% → 60% (～15 min) → 100% (～20 min). Flow rate = 4 mL/min; Temp. = 35.0 °C; UV = 254 nm, C-18 column (Nacalai Tesque : COSMOSIL 5C18-AR-II, 10×250 mm). ^{1}H-NMR (600 MHz, DMSO-*d*$_6$) *δ* 1.34 (quintet, *J* = 7.5 Hz, 2H), 1.45 (quintet, *J* = 7.1 Hz, 2H), 1.86 (quintet, *J* = 7.7 Hz, 2H), 2.27 (t, *J* = 6.6 Hz, 2H), 3.04 (q, *J* = 6.6 Hz, 2H), 3.37 (t, *J* = 5.1 Hz, 2H), 3.5-3.6 (m, 16H), 4.02 (s, 3H), 4.58 (t, *J* = 7.2 Hz, 2H), 6.93 (s, 1H), 7.37 (d, *J* = 7.2 Hz, 1H), 7.42 (dt, *J* = 0.9, 7.5 Hz, 1H), 7.62 (dt, *J* = 1.2, 7.8 Hz, 1H),7.76 (dt, *J* = 0.9, 7.5 Hz, 1H), 7.79 (d, *J* = 8.4 Hz, 1H), 7.81 (t, *J* = 5.4 Hz, 1H), 7.99 (dt, *J* = 1.4, 7.8 Hz, 1H), 8.05 (dd, *J* = 0.9, 8.4 Hz, 1H), 8.14 (d, *J* = 9.0 Hz, 1H), 8.61 (d, *J* = 7.2 Hz, 1H), 8.80 (dd, *J* = 0.6, 9.0 Hz, 1H). ^{13}C-NMR (151 MHz, DMSO-*d*$_6$) *δ* 23.7, 28.9, 29.1, 34.3, 36.6, 38.5, 40.5, 50.4, 54.6, 67.3, 69.7, 69.9, 70.1, 70.2, 70.2, 70.2, 88.5, 108.3, 113.5, 118.6, 123.3, 124.3, 124.7, 125.0, 126.3, 127.2, 128.6, 133.7, 137.5, 140.9, 144.8, 149.0, 160.5, 170.3. ESI-HRMS (*m*/*z*): [M-CF_3COO]$^+$ calcd for $C_{34}H_{45}N_6O_5S^+$, 649.3167, found 649.3185.

Synthesis of TO-3-N_3-2

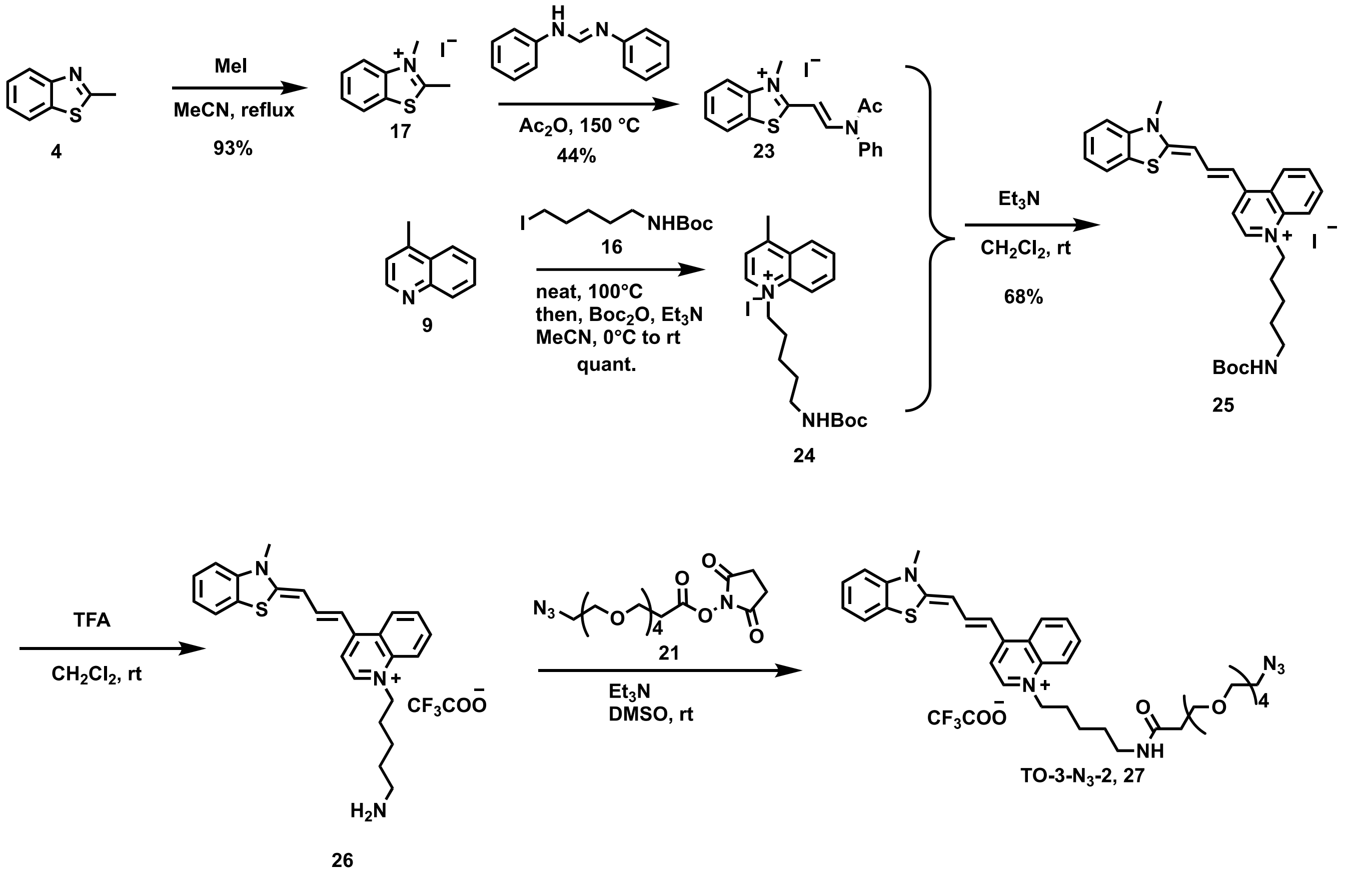

Scheme S5

Synthesis of compound 23

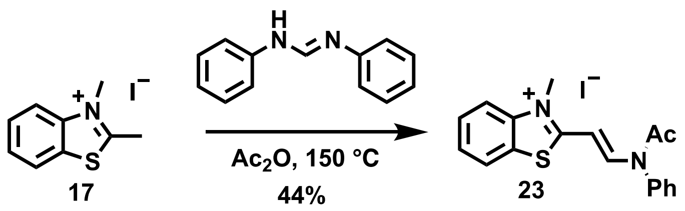

To compound **17** (0.510 g, 1.75 mmol), diphenylformamidine (0.683 g, 3.48 mmol) and Ac_2O (3.5 mL) were added and stirred at 150 °C for 1.5 h. The mixture was cooled to room temperature before Et_2O (30 mL) was added. The mixture was decantated with Et_2O (10 mL×2) and AcOEt (10 mL×2). The mixture was filtrated with AcOEt (10 mL) to afford compound **23** (0.336 g, 44% yield) as a red solid.
^{1}H-NMR (400 MHz, DMSO-d_6) δ 2.06 (s, 3H), 3.88 (3H, s), 5.69 (d, J = 14 Hz, 1H), 7.53 (m, 2H), 7.69 (m, 3H), 7.72 (dd, J = 1.2, 8.0 Hz, 1H), 7.79 (dt, J = 1.2, 7.2 Hz, 1H), 8.10 (d, J = 8.4 Hz, 1H), 8.31 (dd, J = 0.8, 8.0 Hz, 1H), 8.80 (d, J = 14 Hz, 1H). ESI-HRMS (*m*/*z*): $[M-I]^+$ calcd for $C_{18}H_{17}N_2OS^+$, 309.1056, found 309.1069.

Synthesis of compound 24

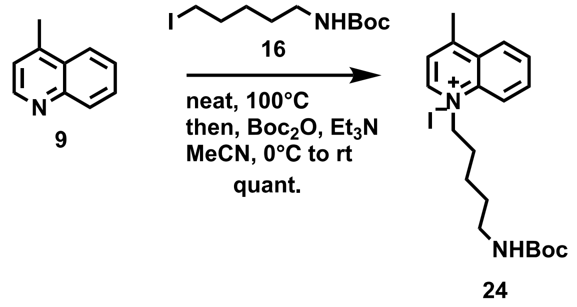

Compound **16** (0.509 g, 1.63 mmol) and lepidine **9** (0.22 g, 1.5 mmol) were added to a round flask. The mixture was stirred for 3.5 h at 100 °C. After the mixture was cooled to room temperature, MeCN (3.2 mL) was added and further cooled to 0 °C. Boc_2O (0.17 g, 0.78 mmol) was added to the stirred solution at 0 °C. After 12 min, the temperature of the solution was raised to room temperature and stirred for 22 h. The mixture was evaporated under reduced pressure and MeCN (1 mL) was added. The solution was decantated with Et_2O (10 mL) and evaporated under reduced pressure. The decantation process was performed 3 times in the end to obtain compound **24** (0.943 g, quant. yield) as an orange solid. The yield was determined by ^{1}H-NMR analysis because T.M. contained byproduct. The compound **24** was used in the next reaction without further purification. ESI-HRMS (*m*/*z*): $[M-I]^+$ calcd for $C_{20}H_{29}N_2O_2^+$, 329.2224, found 329.2248.

Synthesis of compound 25

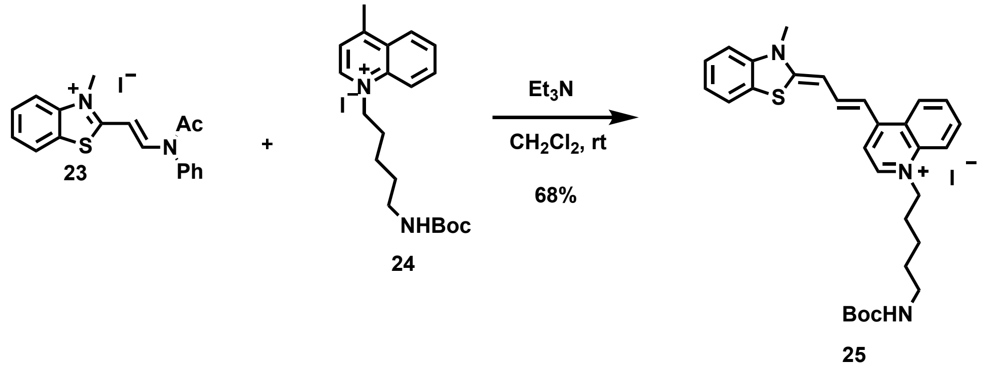

To a solution of compound **24** (0.281 mmol) and compound **23** (0.113 g, 0.259 mmol) in CH_2Cl_2 (5.0 mL), Et_3N (0.23 g, 2.3 mmol) was added and stirred for 21 h at room temperature. The mixture was evaporated under reduced pressure to obtain crude (0.285 g). The crude was purified by column chromatography (hexane/$CHCl_3$ = 1/1 then only $CHCl_3$ then $CHCl_3$/MeOH = 40/1 to 20/1 to 10/1) to afford compound **25** (0.111

g, 68% yield) as a dark green solid. ^{1}H-NMR (600 MHz, DMSO-d_6) δ 1.32 (m, 2H), 1.35 (s, 9H), 1.41 (quintet, J = 7.2 Hz, 2H), 1.82 (quintet, J = 7.2 Hz, 2H), 2.90 (q, J = 6.4 Hz, 2H), 3.74 (s, 3H), 4.53 (m, 2H), 6.49 (d, J = 12 Hz, 1H), 6.78 (t, J = 5.7 Hz, 1H), 7.10 (d, J = 13.2 Hz, 1H), 7.31 (dt, J = 1.2, 6.6 Hz, 1H), 7.49 (dt, J = 1.2, 7.2 Hz, 1H), 7.58 (t, J = 8.4 Hz, 1H), 7.70 (t, J = 7.2 Hz, 1H), 7.84 (dd, J = 1.8, 7.2 Hz, 1H), 7.88 (d, J = 7.8 Hz, 1H), 7.94 (dt, J = 1.2, 7.2 Hz, 1H), 8.07 (d, J = 9.0 Hz, 1H), 8.15 (t, J = 12.9 Hz, 1H), 8.39 (d, J = 7.2 Hz, 1H), 8.46 (d, J = 9.0 Hz, 1H). ^{13}C-NMR (151 MHz, DMSO-d_6) δ 23.2, 28.3, 28.5, 29.1, 32.9, 54.0, 77.4, 98.8, 109.3, 109.6, 112.5, 118.0, 122.5, 124.2, 124.2, 124.6, 125.2, 126.7, 127.6, 133.4, 137.9, 142.0, 142.5, 143.8, 150.3, 155.6, 161.6. ESI-HRMS (m/z): [M-I]$^+$ calcd for $C_{30}H_{36}N_3O_2S^+$, 502.2523, found 502.2542.

Synthesis of compound 27

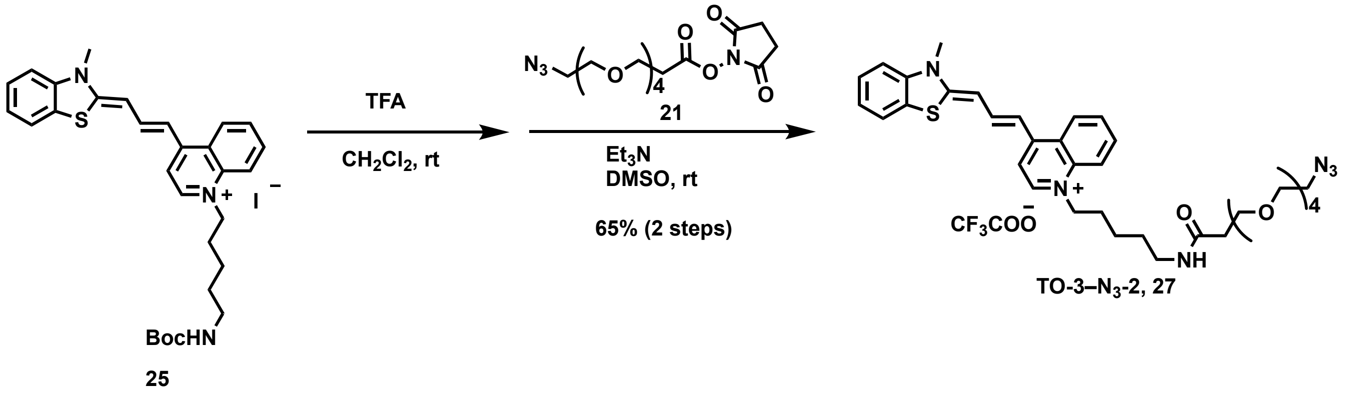

To a solution of compound **25** (13.8 mg, 22.0 μmol) in CH_2Cl_2 (26 μL), TFA (0.25 mL) was added and stirred for 2 h at room temperature. The mixture was evaporated under reduced pressure to obtain the intermediate. To the intermediate in DMSO (1.2 mL), a solution of Azido-PEG$_4$-NHS-Ester **21** (9.39 mg, 24.2 μmol) in DMSO (0.20 mL) and Et_3N (6.2 mg, 61 μmol) were added and stirred for 2 d at room temperature. To the mixture, DMSO (800 μL) was added, and the mixture was filtrated and purified by reverse phased HPLC to afford compound **27** as a blue solid (65% yield in 2 steps). The concentration of **TO-3-N$_3$-2** was determined by quantitative ^{1}H-NMR using maleic acid as an internal standard. HPLC conditions; A: 0.1% TFA in distilled water, B: 0.1% TFA in MeCN; B: 0% → 60% (～15 min) → 100% (～20 min) → 100%(～25 min). Flow rate = 4 mL/min; Temp. = 35.0 °C; UV = 254 nm, C-18 column (Nacalai Tesque: COSMOSIL 5C18-AR-II, 10×250 mm). ^{1}H-NMR (600 MHz, DMSO-d_6) δ 1.32 (m, 2H), 1.43 (quintet, J = 7.2 Hz, 2H), 1.82 (quintet, J = 7.7 Hz, 2H), 2.27 (t, J = 6.6 Hz, 2H), 3.03 (q, J = 6.0 Hz, 2H), 3.36 (t, J = 4.8 Hz, 2H), 3.45-3.58 (m, 16H), 3.72 (s, 3H), 4.51 (t, J = 7.5 Hz, 2H), 6.47 (d, J = 12.6 Hz, 1H), 7.08 (d, J = 13.2 Hz, 1H), 7.30 (dt, J = 0.9, 7.8 Hz, 1H), 7.48 (dt, J = 0.8, 7.5 Hz, 1H), 7.56 (d, J = 8.4 Hz, 1H), 7.69 (dt, J = 0.6, 7.2 Hz, 1H), 7.81 (brs, 1H), 7.82 (d, J = 7.2 Hz, 1H), 7.86 (dd, J = 1.2, 7.8 Hz, 1H), 7.94 (dt, J = 1.2, 7.8 Hz, 1H), 8.05 (d, J = 9.0 Hz, 1H), 8.14 (dt, J = 1.2, 12.9 Hz, 1H), 8.36 (d, J = 7.2 Hz, 1H), 8.45 (dd, J = 1.2, 7.8 Hz, 1H). ^{13}C-NMR (151 MHz, DMSO-d_6) δ 23.3, 28.5, 28.7, 32.9, 36.2, 38.1, 50.0, 54.0, 66.9, 69.3, 69.6, 69.7, 69.8, 69.8, 69.9, 98.9, 109.3, 109.6, 112.6, 115.8, 117.8, 118.0, 122.6, 124.2, 124.3, 124.7, 125.2, 126.8, 127.7, 133.4, 137.9, 142.0, 142.5, 143.9, 150.4, 157.9, 158.1, 161.7, 163.1, 170.0. ESI-HRMS (m/z): [M-CF$_3$COO]$^+$ calcd for $C_{36}H_{47}N_6O_5S^+$, 675.3323, found 675.3340.

Biotin modification of G-clamp-N_3 by SPAAC

To a solution of DBCO-SS-biotin in DMSO (Argutus Medical Ltd., 10 mM, 50 μL), a solution of G-clamp-N_3 in DMSO (10 mM, 50 μL) was added, and the mixture (5 mM, 100 μL) was incubated at 37 °C. After 30 min, the mixture was analyzed by reverse phased HPLC with C-18 column (Nacalai Tesque: COSMOSIL 5C_{18}-AR-II, 4.6 × 250 mm) by a liner gradient of 10-100%/20 min acetonitrile in 0.1% TFA buffer at a flow rate of 1 mL/min at 40 °C, and monitored by UV detection at λ = 254 nm (Figure S1). The mixture was used without any purification. G-clamp-biotin: ESI-HRMS (*m*/*z*): $[M+H]^+$ calcd for $C_{64}H_{87}N_{14}O_{15}S_3^+$, 1387.5632; found 1387.5620.

Biotin modification of TO-N_3, TO-N_3-2, TO-3-N_3, and TO-3-N_3-2 by SPAAC

To a solution of DBCO-biotin in DMSO (9 mM, 1 μL), a solution of TO-N_3, TO-N_3-2, TO-3-N_3, or TO-3-N_3-2 in DMSO (10 mM, 1 μL) was added, and the mixture (4.5 and 5 mM, 2 μL) was incubated at 37 °C. After 30 min, the mixture was analyzed by reverse phased HPLC with C-18 column (Nacalai Tesque: COSMOSIL 5C_{18}-AR-II, 4.6 × 250 mm) by a liner gradient of 10-100%/20 min acetonitrile in 0.1% TFA buffer at a flow rate of 1 mL/min at 40 °C and monitored by UV detection at λ = 254 nm (Fig. S2). The mixture was used without any purification. TO-biotin: ESI-HRMS (*m*/*z*): $[M+2H]^{2+}$ calcd for $C_{70}H_{91}N_{11}O_{12}S_2^{2+}$, 670.8142; found 670.8142, TO-3-bitotin: ESI-HRMS (*m*/*z*): $[M+2H]^{2+}$ calcd for $C_{72}H_{93}N_{11}O_{12}S_2^{2+}$, 683.8218; found 683.8220, TO-biotin-2: ESI-HRMS (*m*/*z*): $[M+2H]^{2+}$ calcd for $C_{73}H_{97}N_{11}O_{13}S_2^{2+}$, 699.8349; found 699.8357, TO-3-biotin-2: ESI-HRMS (*m*/*z*): $[M+2H]^{2+}$ calcd for $C_{75}H_{99}N_{11}O_{13}S_2^{2+}$, 712.8427; found 712.8439.

Supplementary Figures

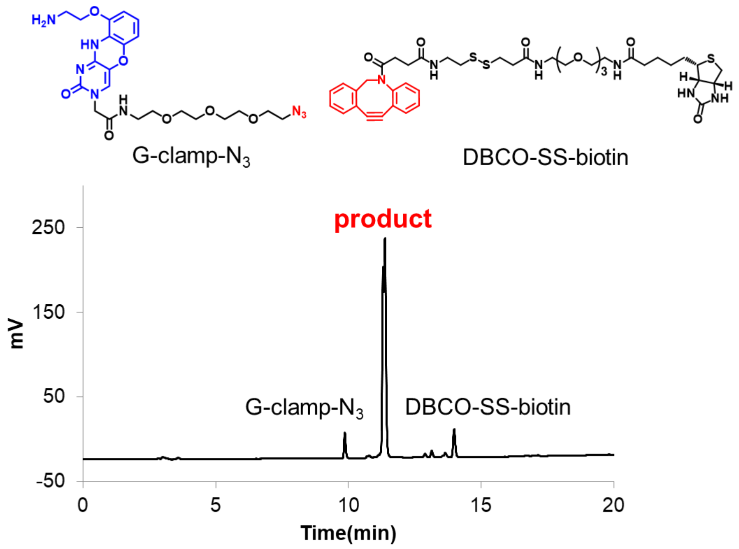

Figure S1. HPLC profiles of biotin conjugation of G-clamp-N_3 by strain-promoted azide-alkyne cycloaddition (SPAAC).

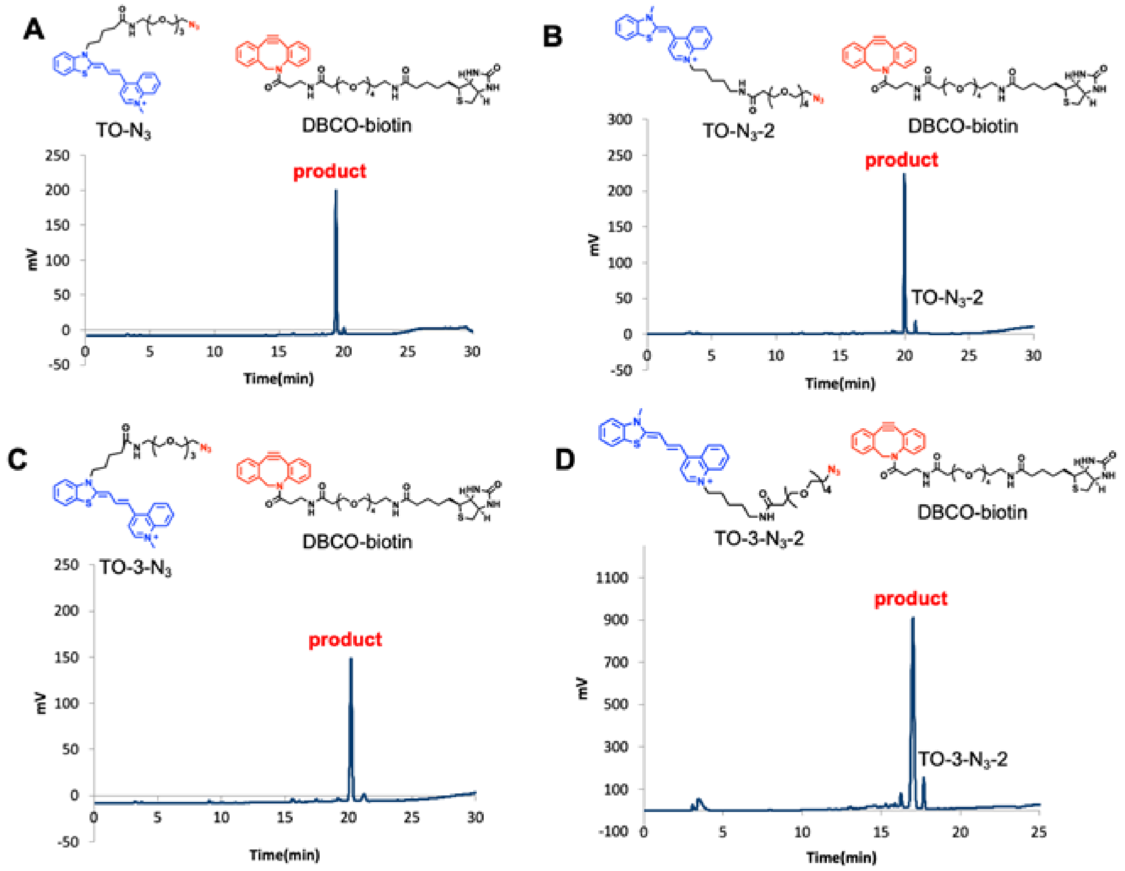

Figure S2. HPLC profiles of biotin conjugation of TO derivatives by strain-promoted azide-alkyne cycloaddition (SPAAC). The reactions of (A) TO-N_3, (B) TO-N_3-2, (C) TO-3-N_3-1, and (D) TO-3-N_3-2.

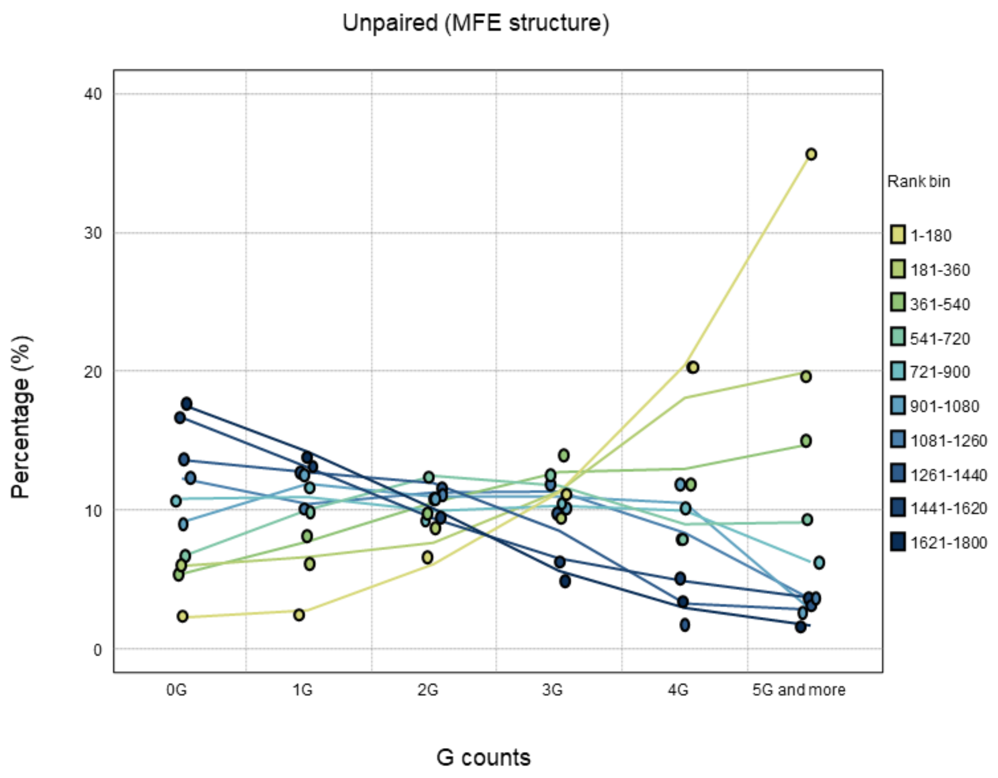

Figure S3. Evaluation of the relationship between the number of multiple Gs and the affinity ranking of G-clamp to human pre-miRNA (n = 1800) in library-1. MFE (minimum free energy) structures calculated by RNAfold were used to count G in unpaired nucleotide (ssRNA). The X-axis shows bins determined by the number of Gs in each secondary structure; the Y-axis shows the number of RNA motifs in each bin of G counts as a percentage.

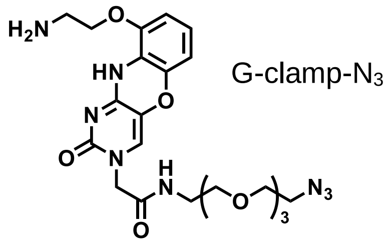

Figure S4. Fluorescence titrations to determine the apparent dissociation constants (K_{Dapp}) of G-clamp-N_3. Fluorescence titration spectra were measured using G-clamp-N_3 (0.01 μM for hsa-mir-4520-1, its mutG2A, and hsa-mir-6847, or 0.1 μM for other RNAs) upon addition of RNA (0-21 μM) in phosphate buffer (pH 7.0). The representative data and K_{Dapp} values are shown.

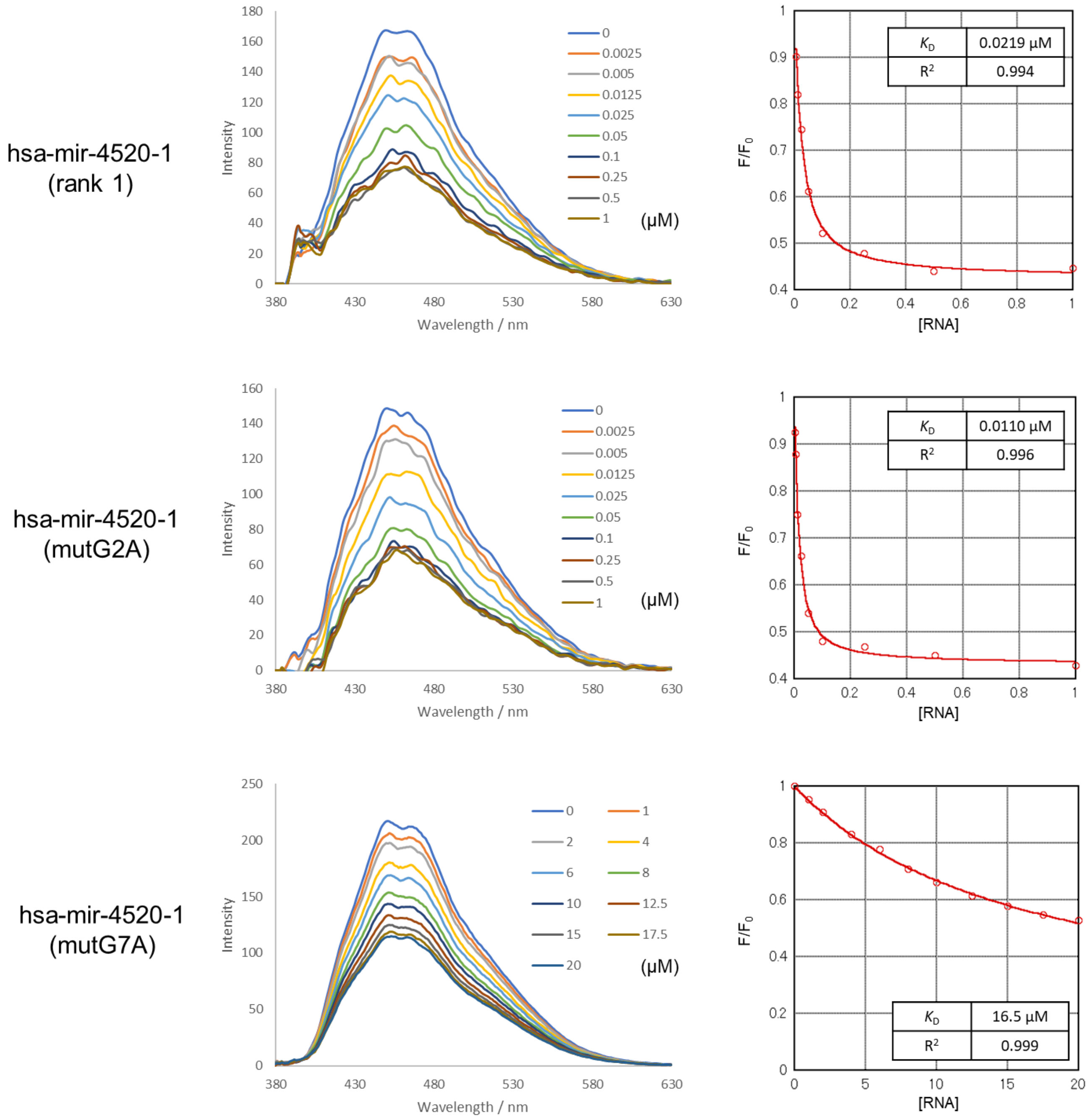

hsa-mir-6847
(rank 2)

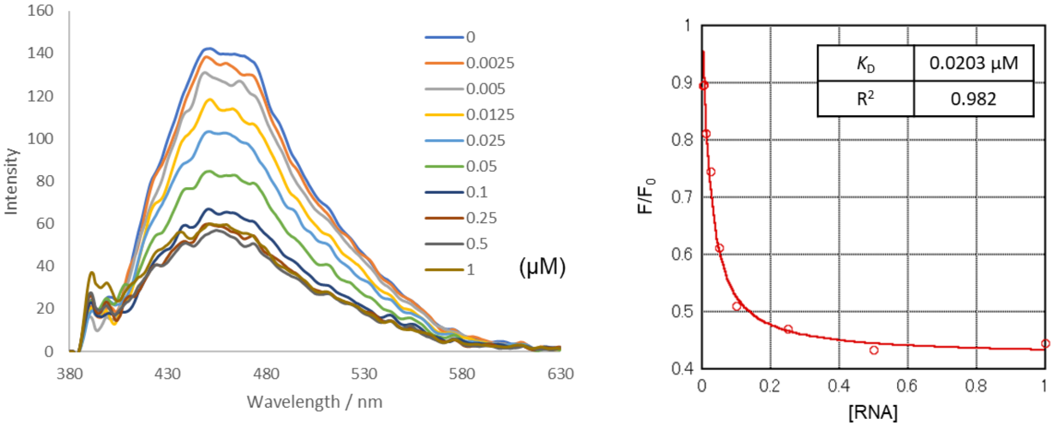

hsa-mir-125a
(rank 4)

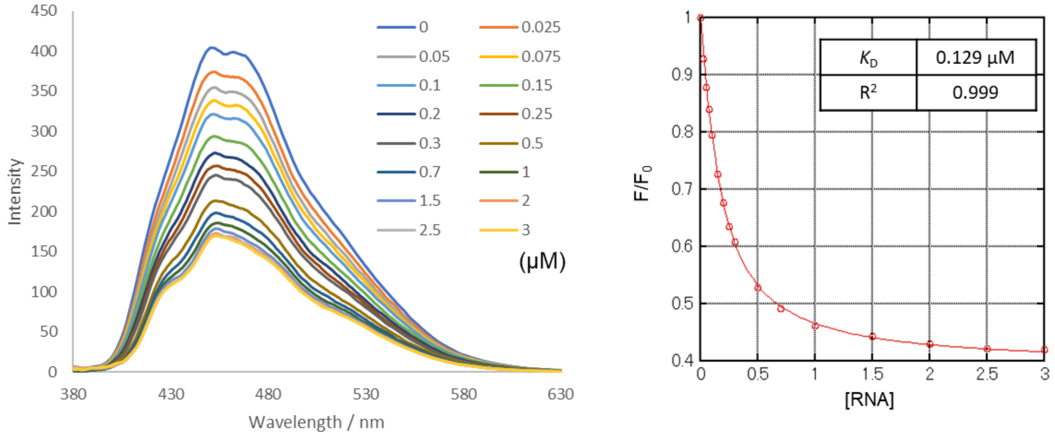

hsa-mir-6790
(rank 13)

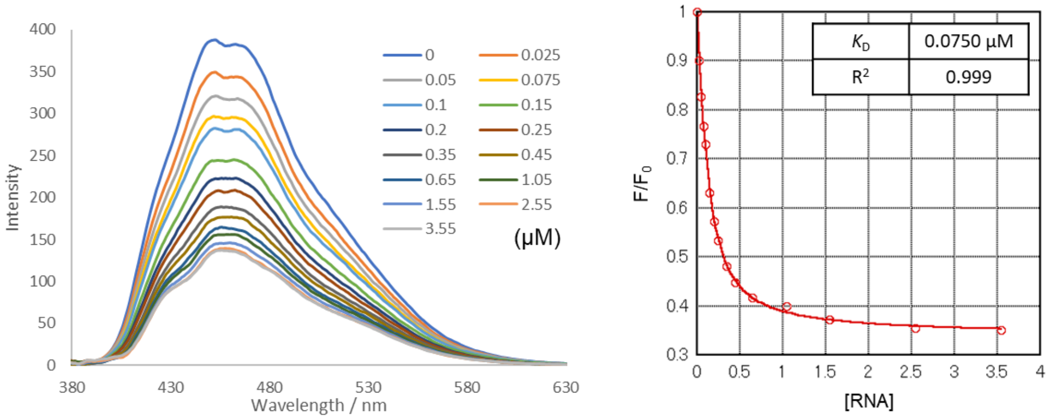

hsa-mir-6850
(rank 28)

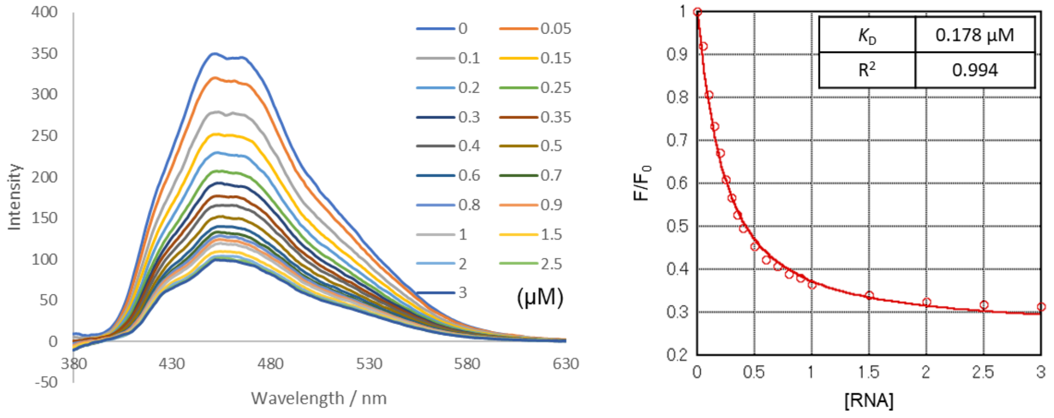

G4_(GGGU)$_6$
(rank 38)

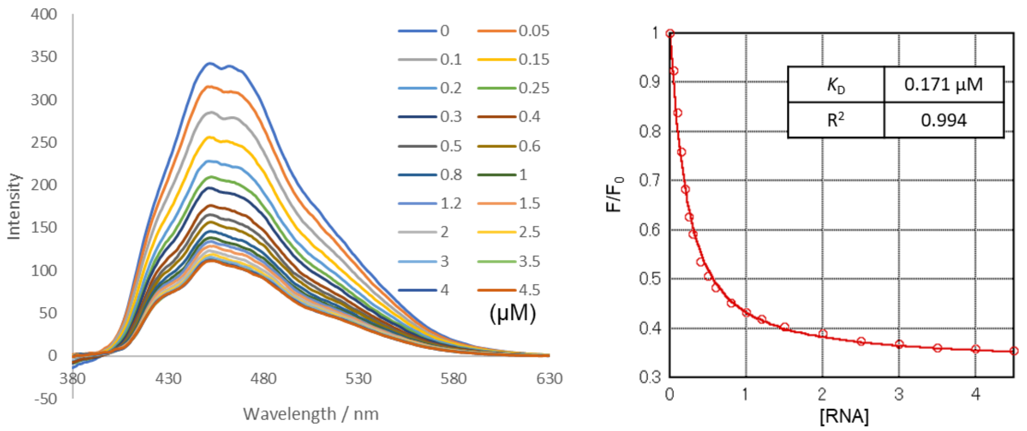

r(CUG)$_{16}$
(rank 43)

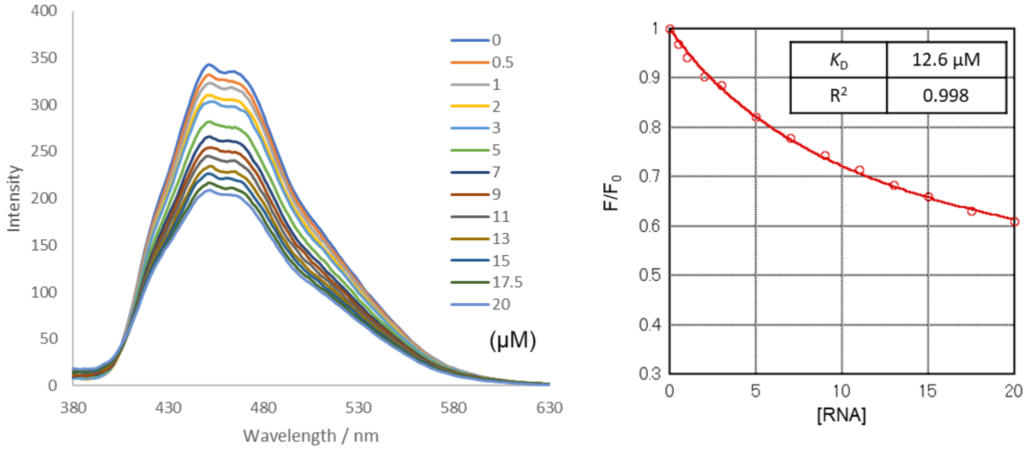

hsa-mir-4291
(rank 83)

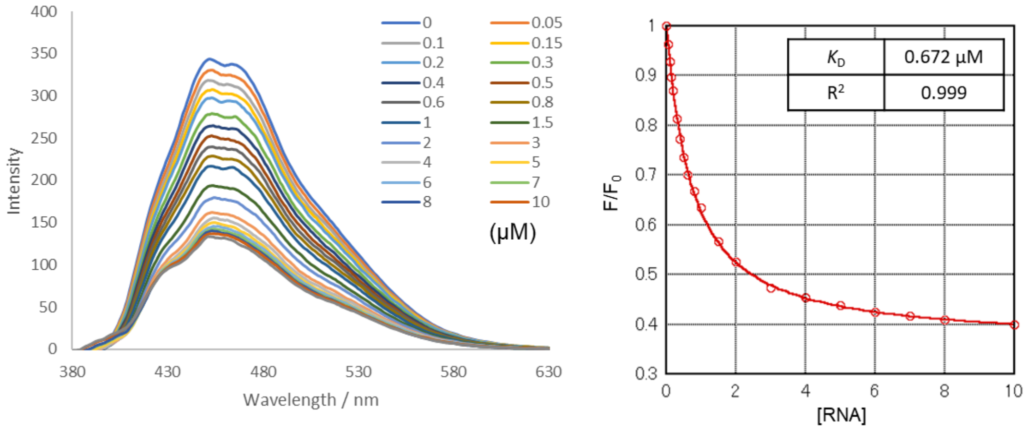

hsa-mir-526a-1
(rank 111)

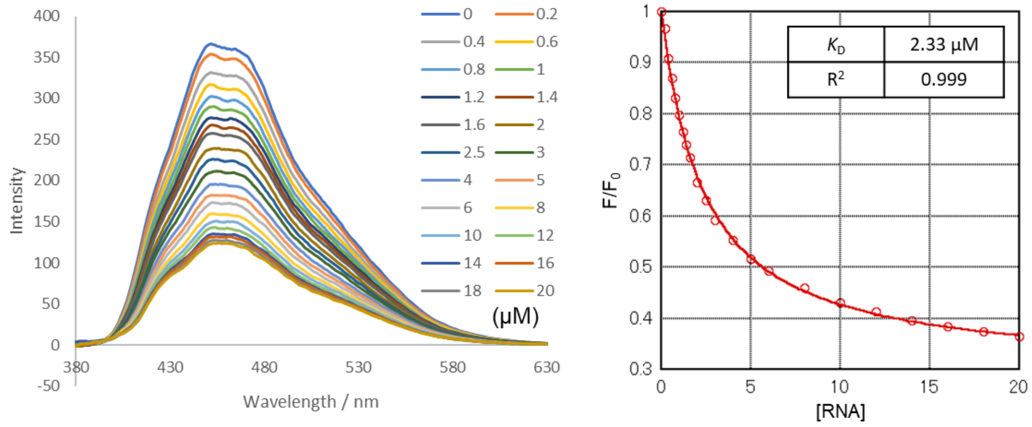

hsa-mir-105-1
(rank 160)

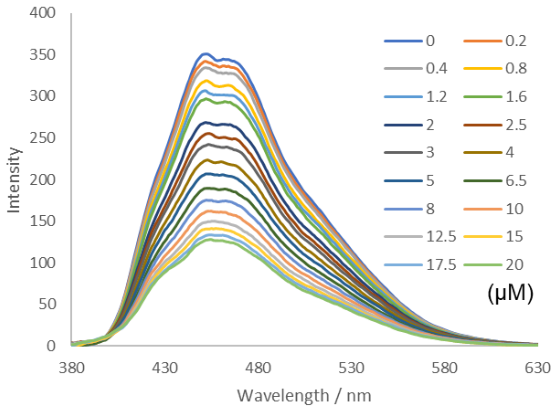

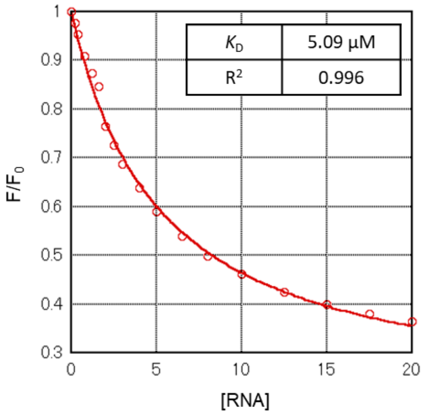

hsa-mir-100
(rank 225)

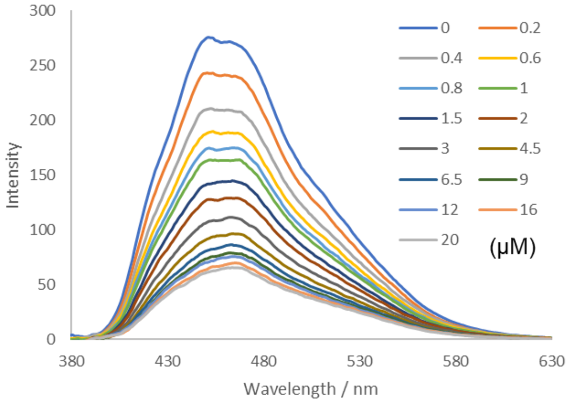

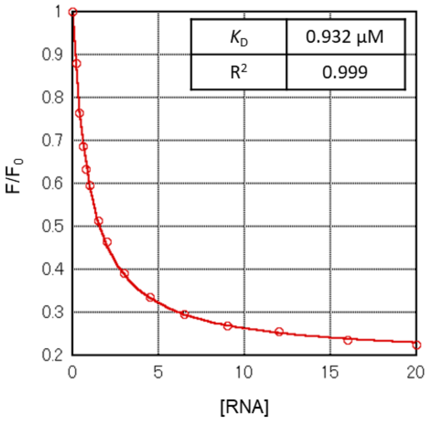

hsa-mir-548ba
(rank 522)

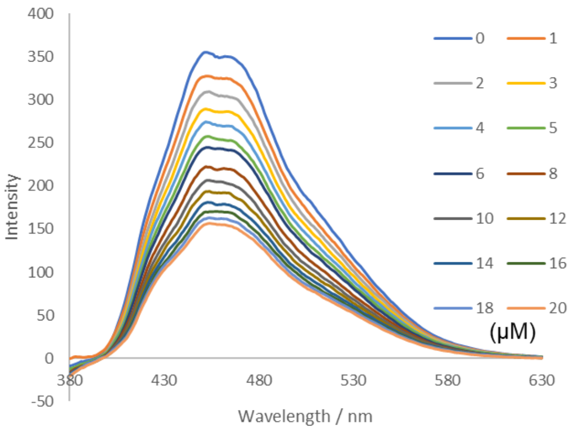

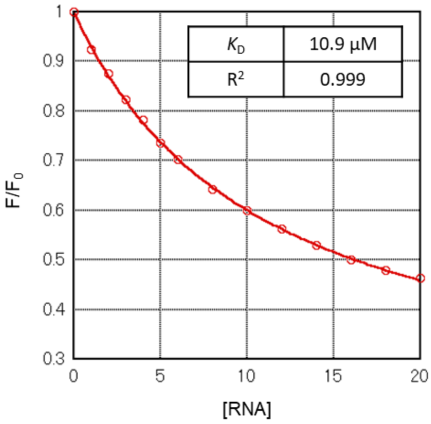

hsa-mir-203b
(rank 633)

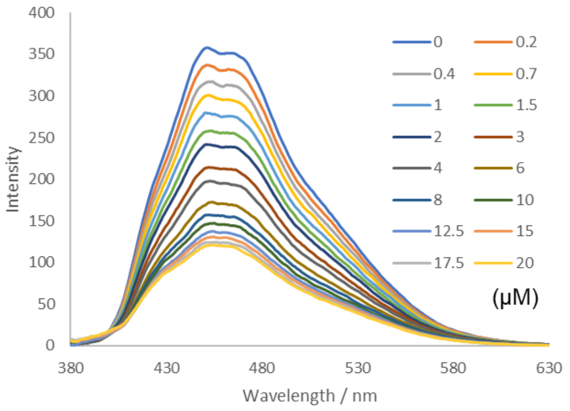

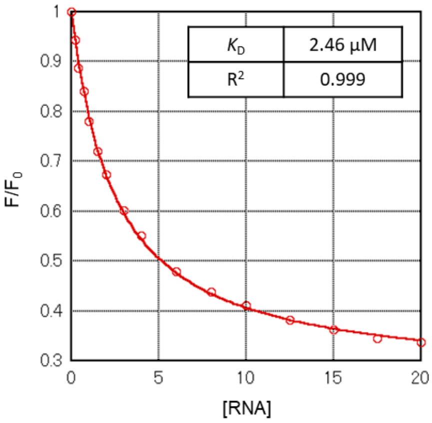

hsa-mir-6786
(rank 945)

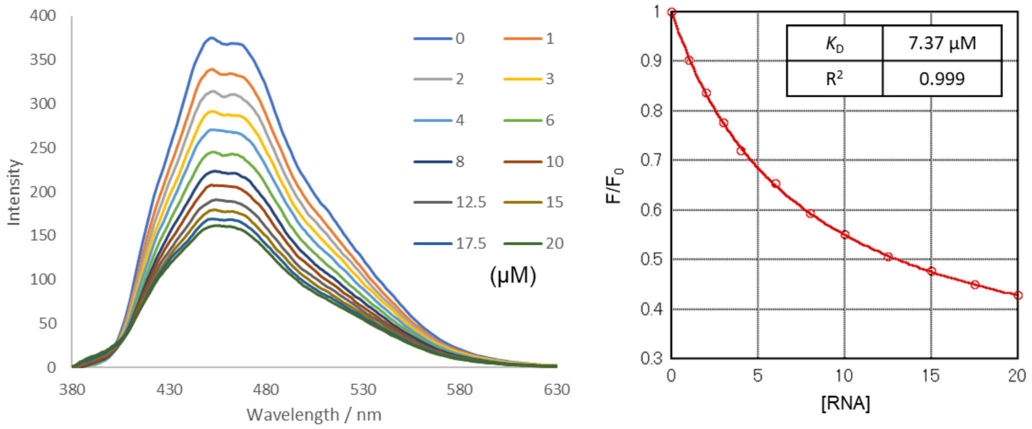

hsa-mir-299
(rank 1034)

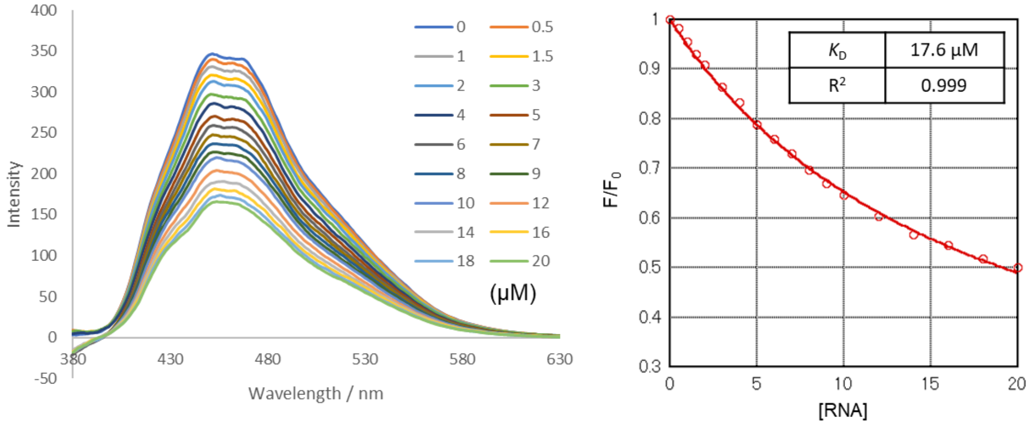

hsa-mir-4773-1
(rank 1192)

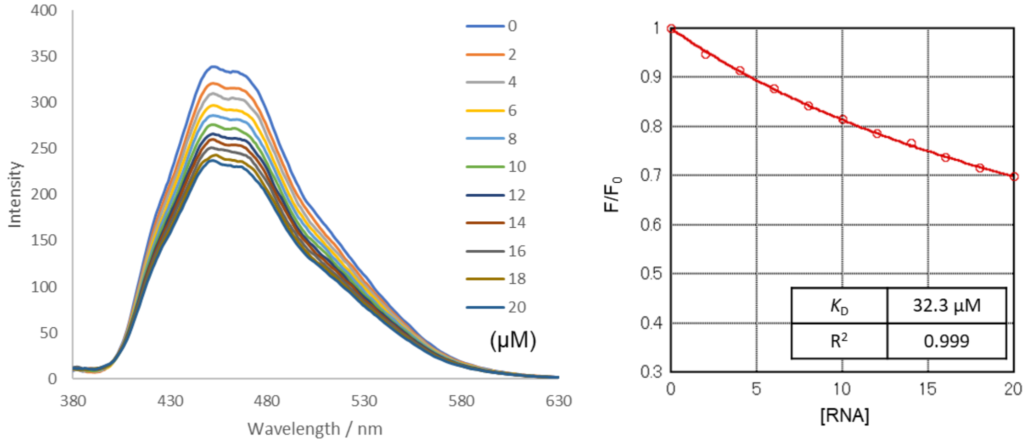

hsa-mir-4282
(rank 1775)

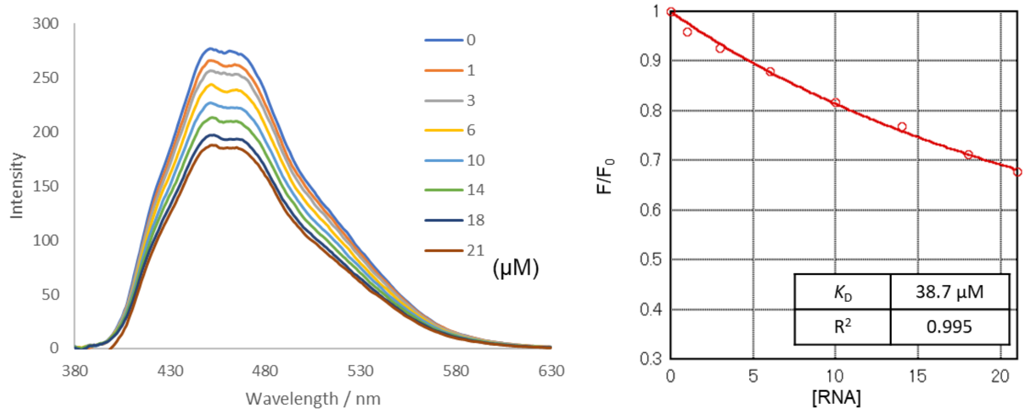

Common stem

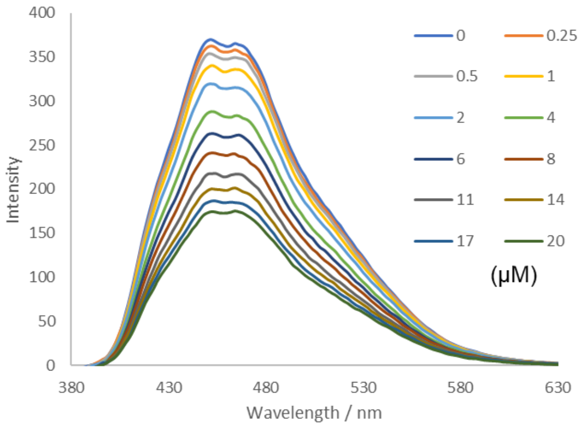

Intensity
Wavelength / nm
0
0.25
0.5
1
2
4
6
8
11
14
17
20
(μM)

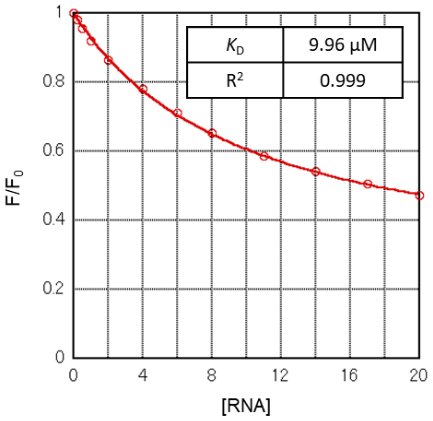

K_D 9.96 μM
R^2 0.999
F/F_0
[RNA]

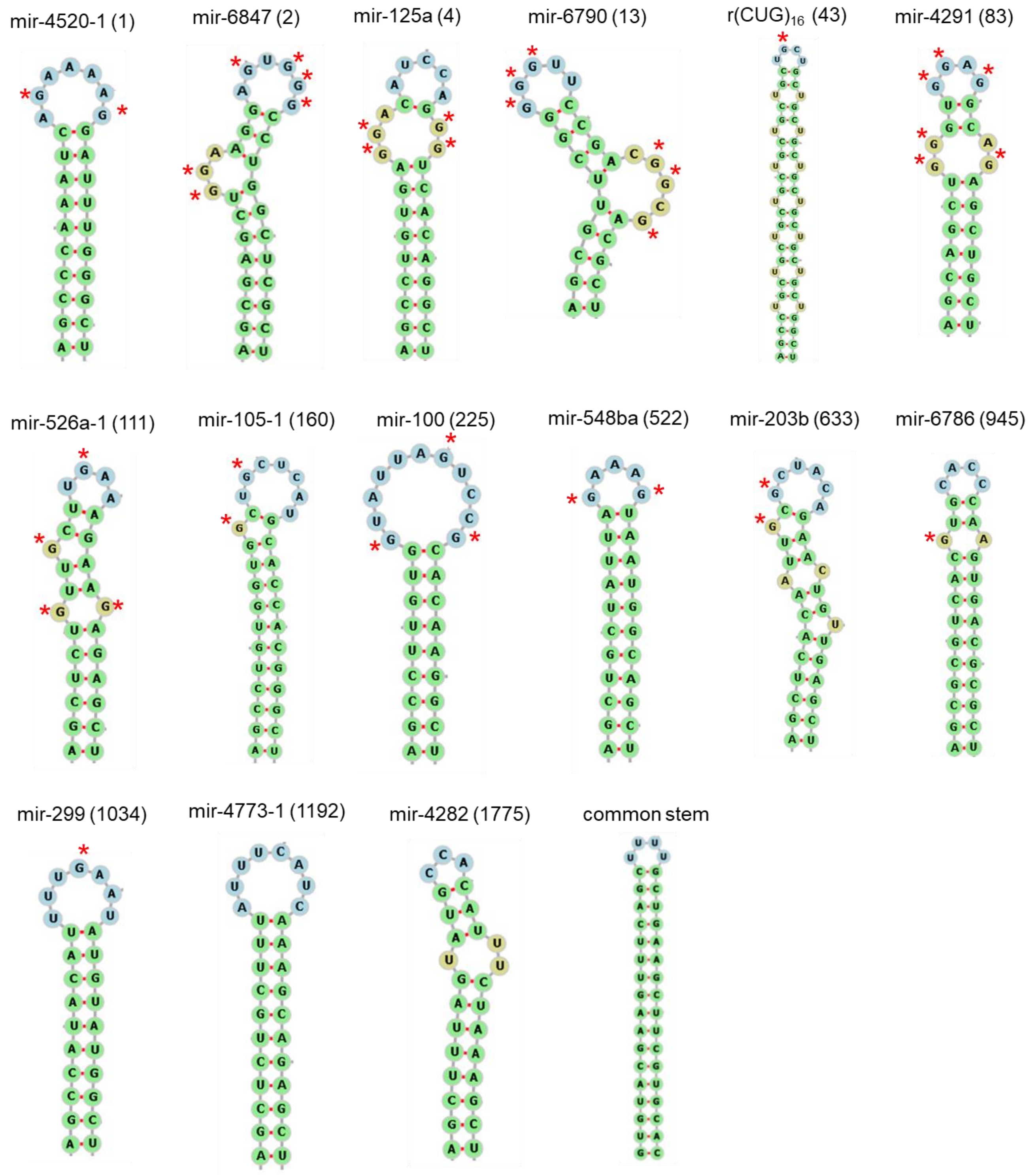

Figure S5. Minimum free energy structures calculated by RNAfold. The red asterisks (*) indicate the unpaired G base.

Table S1. The correlation between Z-scores and K_{Dapp} values for G-clamp RNA binding.

Rank	Name	Seq.	Z-score	K_{Dapp} (μM) [a]
1	hsa-mir-4520-1	CCAAAUCAGAAAAGGAUUUGG	4.82	0.024
-	mutG2A	CCAAAUCA**A**AAAAGGAUUUGG	-	0.011
-	mutG7A	CCAAAUCAGAAAA**A**GAUUUGG	-	15
-	mutG2,7A	CCAAAUCA**A**AAAA**A**GAUUUGG	-	3.7
2	hsa-mir-6847	GAGCUGGAAGGAGUGGGCCUGGCUC	4.26	0.022
4	hsa-mir-125a	CUGUGAGGACAUCCAGGGUCACAG	3.77	0.14
13	hsa-mir-6790	GUUCGGGGGUUCCGACGGCGAC	3.11	0.071
28	hsa-mir-6850	CGGGGCGGGAGGGGAAGGGACGCCCG	2.77	0.19
38	$(GGGU)_6$	GGGUGGGUGGGUGGGUGGGUGGGU	2.59	0.15
43	$(CUG)_{16}$	$(CUG)_{16}$	2.53	13
83	hsa-mir-4291	AGCUGGGUGGAGGCAGAGCU	2.00	0.56
111	hsa-mir-526a-1	UCUGUUGCUUGAAAGAAGAGA	1.72	2.3
160	hsa-mir-105-1	CUGUGGUGGCUGCUCAUGCACCACGG	1.49	5.4
225	hsa-mir-100	CUUGUGGUAUUAGUCCGCACAAG	1.22	1.1
522	hsa-mir-548ba	UGCUAUUAGAAAGUAAUGGCA	0.53	10
633	hsa-mir-203b	UCACAAUUGCGCUACAGAACUGUUGA	0.33	2.9
945	hsa-mir-6786	GCGUCACGUGCACCCAAGUGACGC	-0.12	6.6
1034	hsa-mir-299	CAUACAUUUUGAAUAUGUAUG	-0.22	16
1192	hsa-mir-4773-1	UCUGCUUUAUUUCAUCAAAGCAGA	-0.40	>20
1775	hsa-mir-4282	UUUAGUAUGCCACAUUUCUAAA	-1.44	>20
-	Common stem	GUGUACGAAGUUUCAGCUUUUGCUGAAGCUU CGUGCAC	-	9.0

a) Mean data from two independent experiments are shown.

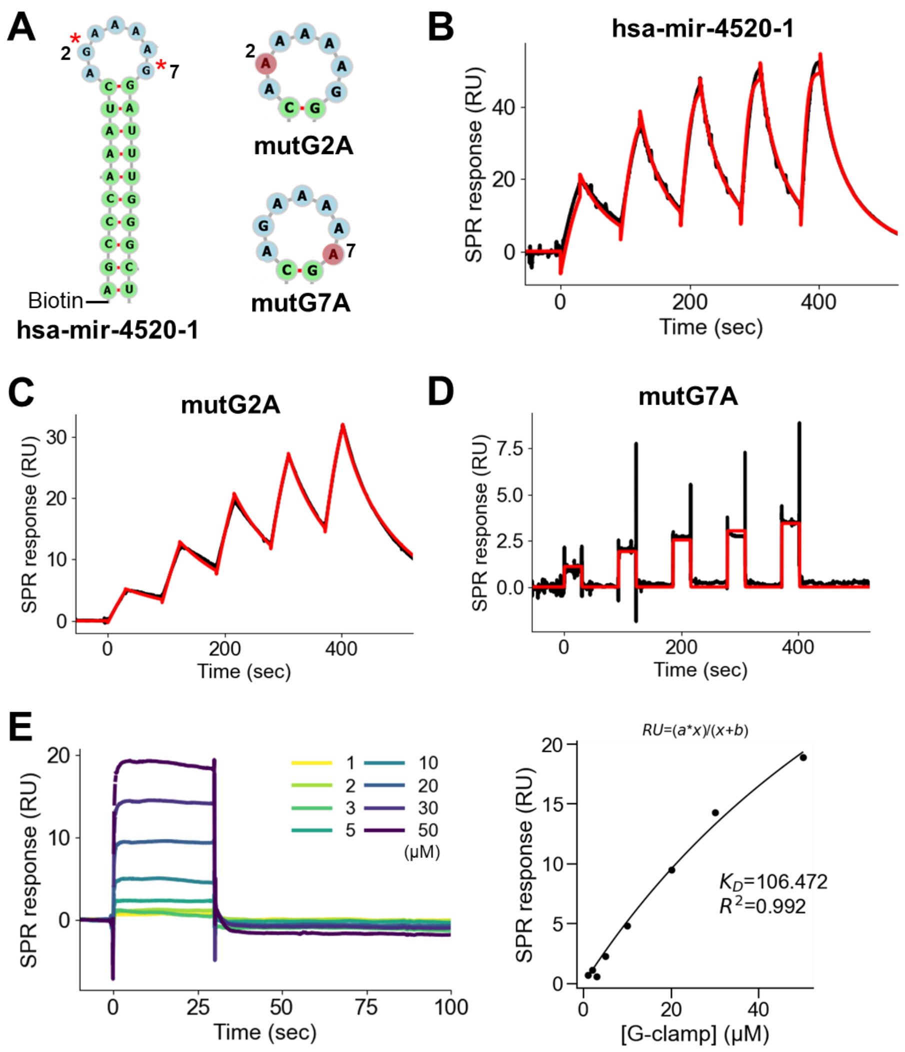

G

RNA	k_a (1/μM·s)	k_d (1/s)	K_{Dapp} (μM)	K_{Dapp} (μM)1)
hsa-mir-4520-1	0.67±0.5	0.049±0.02	0.10±0.02	0.024
mutG2A	0.76±0.4	0.027±0.01	0.044±0.008	0.011
mutG7A	-	-	>50[2)]	15

1) The K_{Dapp} value was determined by fluorescence titration (n = 2).
2) The K_{Dapp} value was determined by multi-cycle kinetic mode.

Figure S6. Surface plasmon resonance (SPR) analysis for G-clamp-N_3. (A) Minimum free energy structure of hsa-mir-4520-1 and its mutants. The 5'-end was modified with biotin. (B-D) The representative SPR sensorgram of G-clamp-N_3 (100, 200, 300, 400, and 500 nM for hsa-mir-4520-1 (B); 20, 40, 60, 80, and 100 nM for G2A mutant (C); 1000, 2000, 3000, 4000, 5000 nM for G7A mutant (D)) binding to the RNA. The black line represents the SPR response with G-clamp and the red line represents the fitting curve. The binding kinetics were determined in single-cycle kinetic mode. (E) The representative SPR sensorgram of G-clamp-N_3 (1, 2, 3, 5, 10, 20, 30, and 50 μM) binding to G7A mutant RNA. The K_{Dapp} value was determined by multi-cycle kinetic mode. (F) Summary table of the SPR results. The data are mean±SE (n = 3).

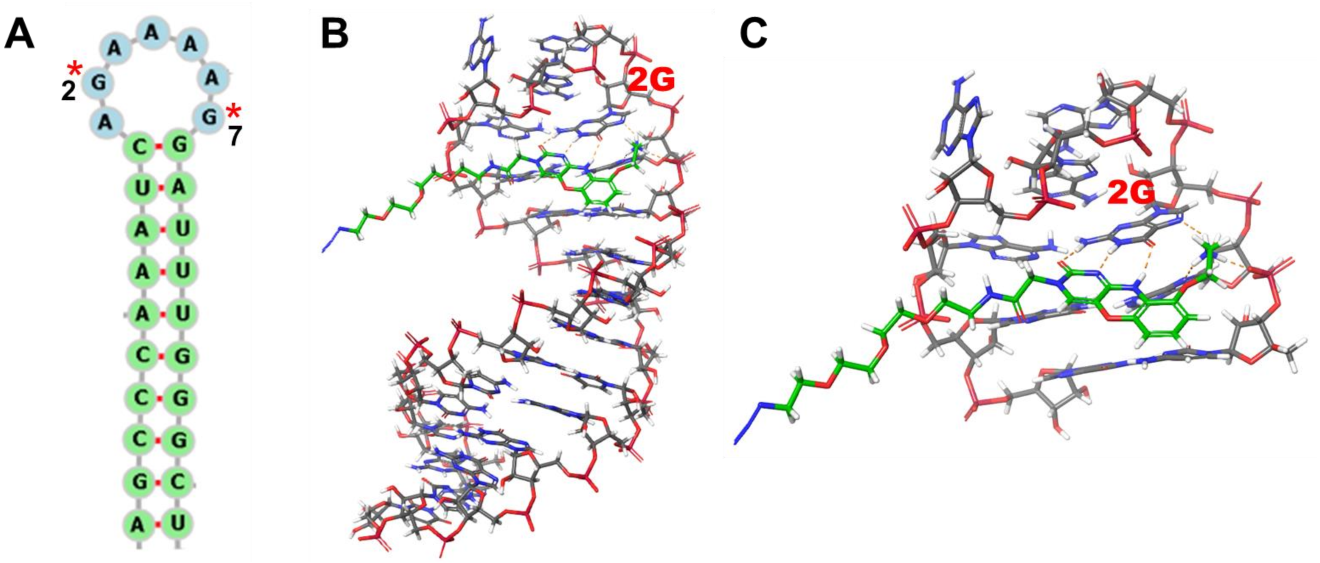

Figure S7. Molecular modeling of the complex structure between hsa-mir-4520-1 and G-clamp–N_3. G-clamp–N_3 binds to 2G. (A) Minimum free energy structure of hsa-mir-4520-1 loop motif. The red asterisks (*) indicate the G base in the single-stranded region. (B) Molecular modeling of the complex structure between hsa-mir-4520-1 and G-clamp–N_3. G-clamp–N_3 binds to 2G. The green molecule indicates G-clamp–N_3. The orange dash line indicates the hydrogen bond. The complex structure was modeled by RNAComposer and MacroModel. (C) Modeling structure focusing on interactions with G-clamp. The green molecule indicates G-clamp–N_3.

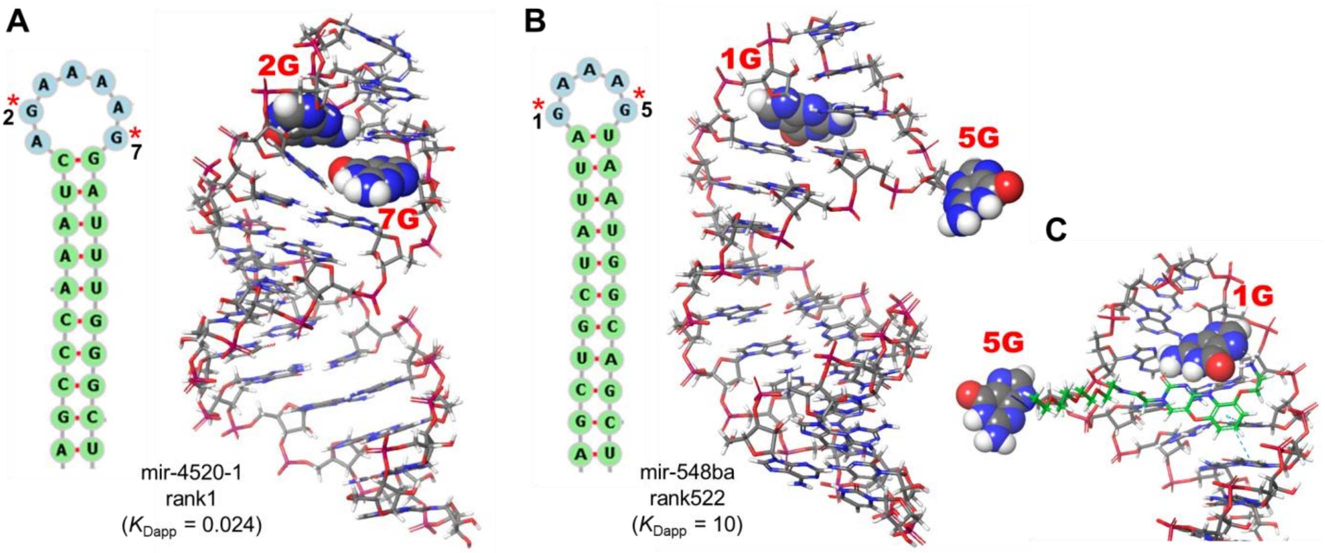

Figure S8. Molecular modeling structure of (A) hsa-mir-4520-1, (B) hsa-mir-548ba and (C) the complex between hsa-mir-548ba and G-clamp-N_3. The structure was modeled by RNAComposer and MacroModel. Red asterisks (*) and CPK models indicate the G base in single-stranded regions. The green molecule indicates G-clamp–N_3. The orange dash and blue lines indicate the hydrogen bond and stacking interaction, respectively.

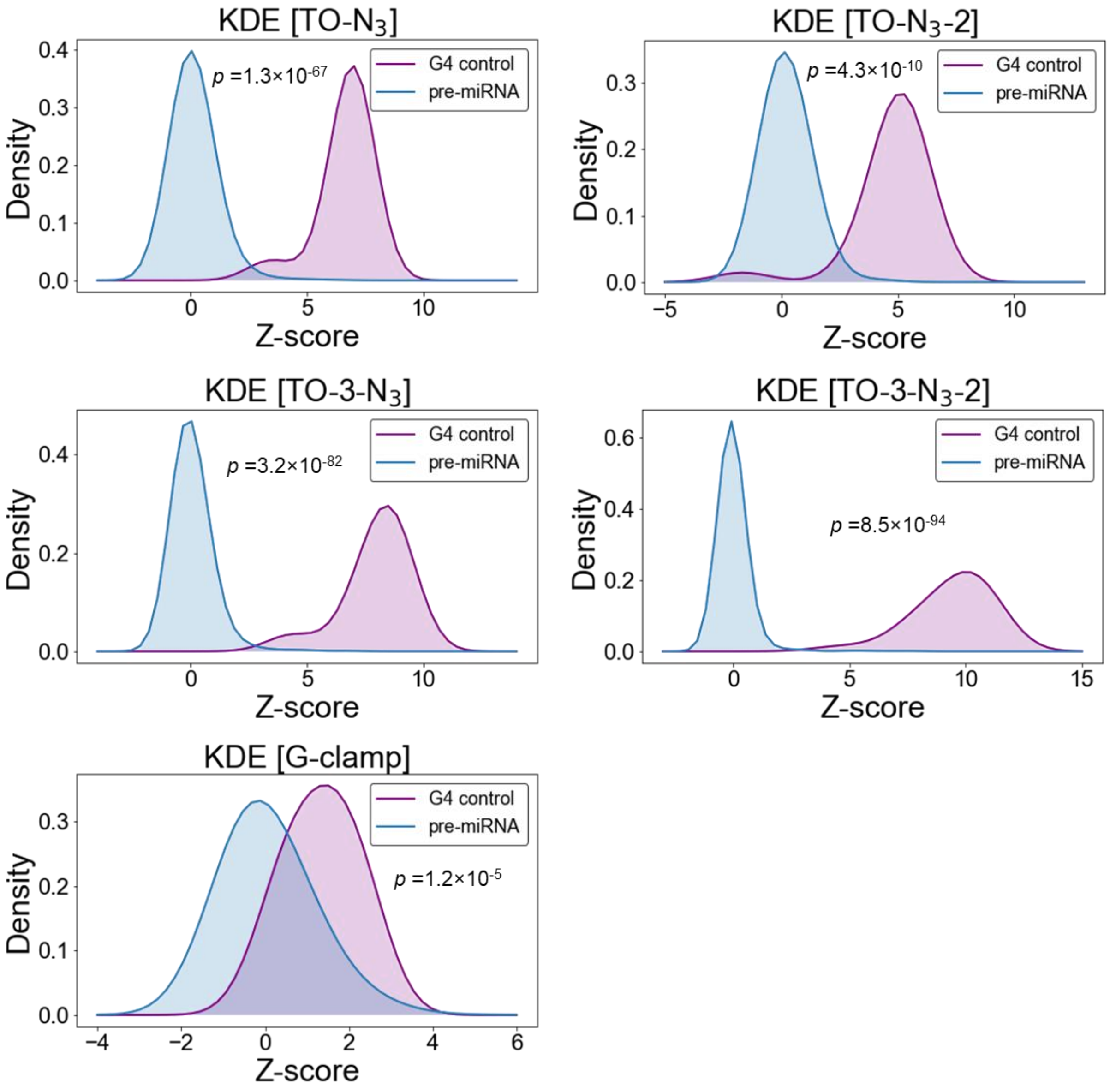

Figure S9. Kernel density estimation of TO derivatives and G-clamp Z-scores. The *p*-values were determined by two-tailed Brunner–Munzel test.

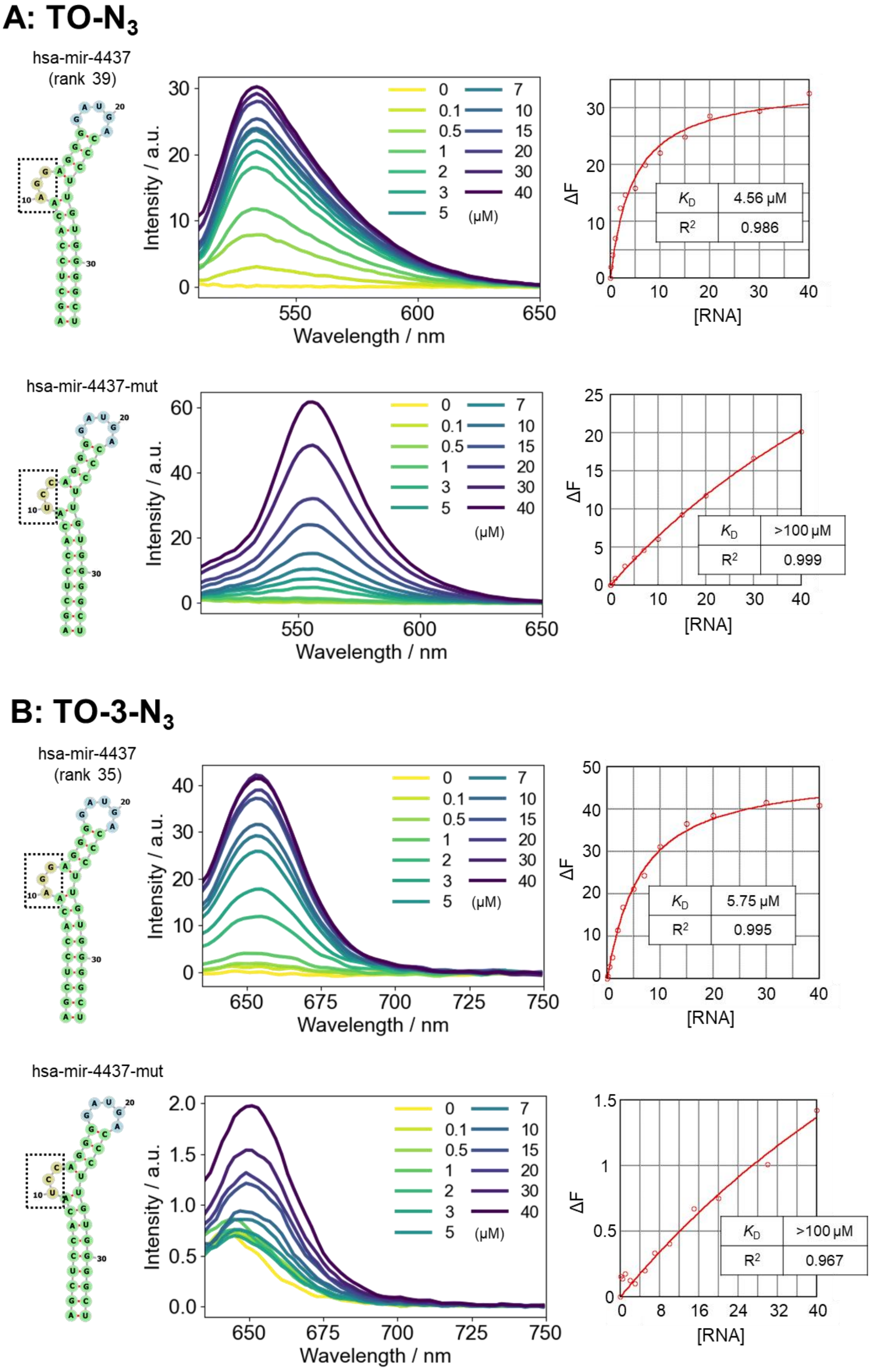

Figure S10. Fluorescence titrations to compare the K_{Dapp} values for a hsa-mir-4437 loop with mir-4437-mut (AGG to UCC). Fluorescence titration spectra were measured using (A) TO-N_3 (0.1 μM) or (B) TO-3-N_3 (1 μM) upon addition of RNA (0-40 μM) in phosphate buffer (pH 7.0). Conditions: λex: 501 nm, λem: 530 nm for TO-N_3 and λex: 623 nm, λem: 655 nm for TO-3-N_3. The representative data and K_{Dapp} values are shown.

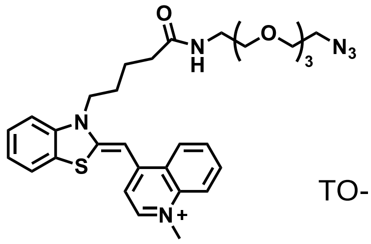

TO-N_3

Figure S11. Fluorescence titrations to determine the apparent dissociation constants (K_{Dapp}) of TO-N_3. Fluorescence titration spectra were measured using TO-N_3 (0.1 μM) upon addition of RNA (0-40 μM) in phosphate buffer (pH 7.0). Conditions: λex: 501 nm, λem: 530 nm. The representative data and K_{Dapp} values are shown.

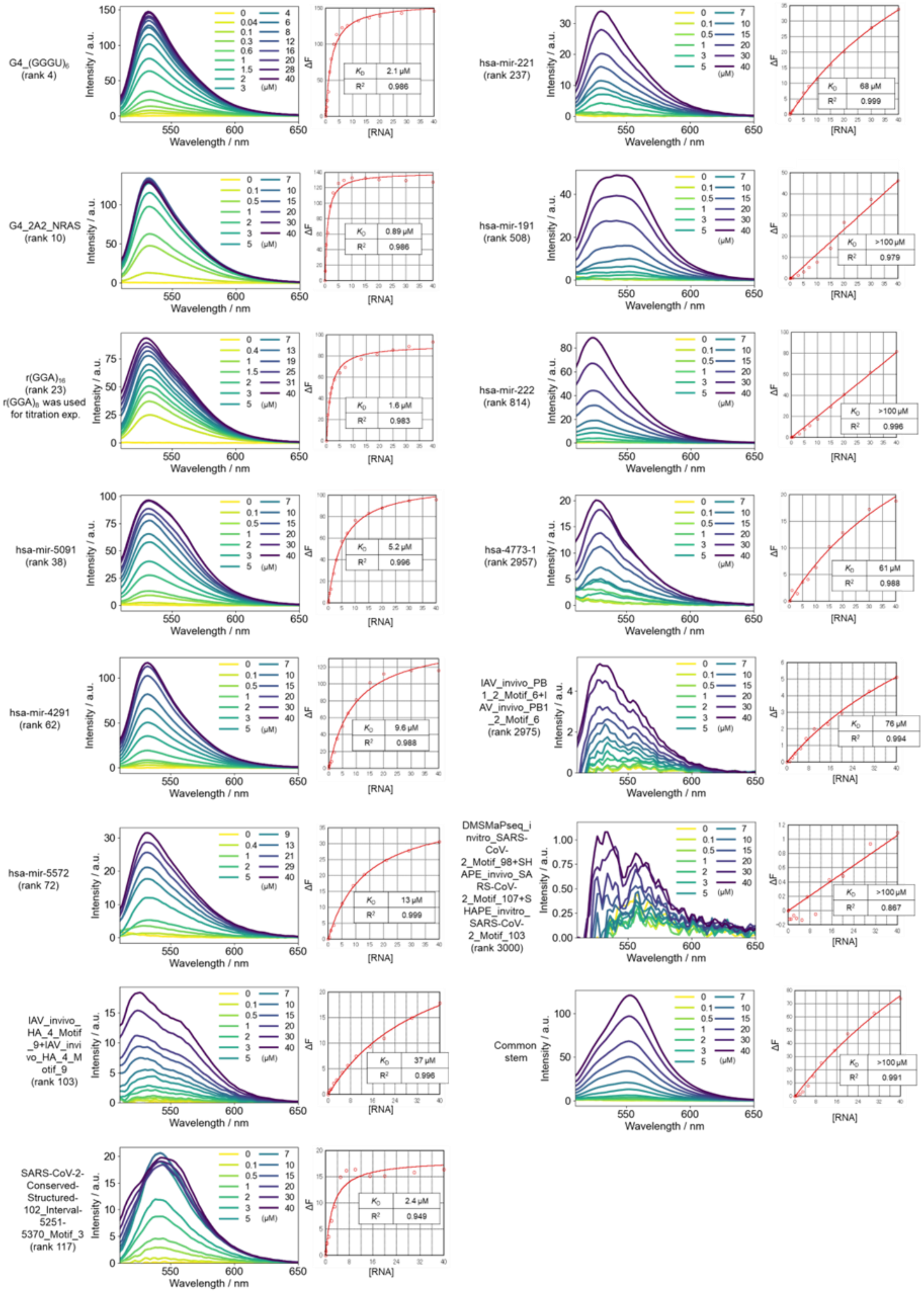

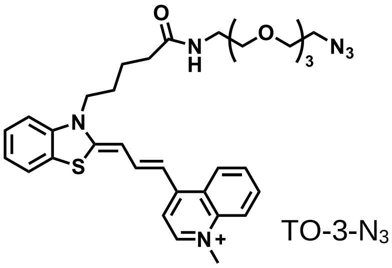

TO-3-N$_3$

Figure S12. Fluorescence titrations to determine the apparent dissociation constants (K_{Dapp}) of TO-3-N$_3$. Fluorescence titration spectra were measured using TO-3-N$_3$ (1 µM) upon addition of RNA (0-40 µM) in phosphate buffer (pH 7.0). Conditions: λex: 623 nm, λem: 655 nm. The representative data and K_{Dapp} values are shown.

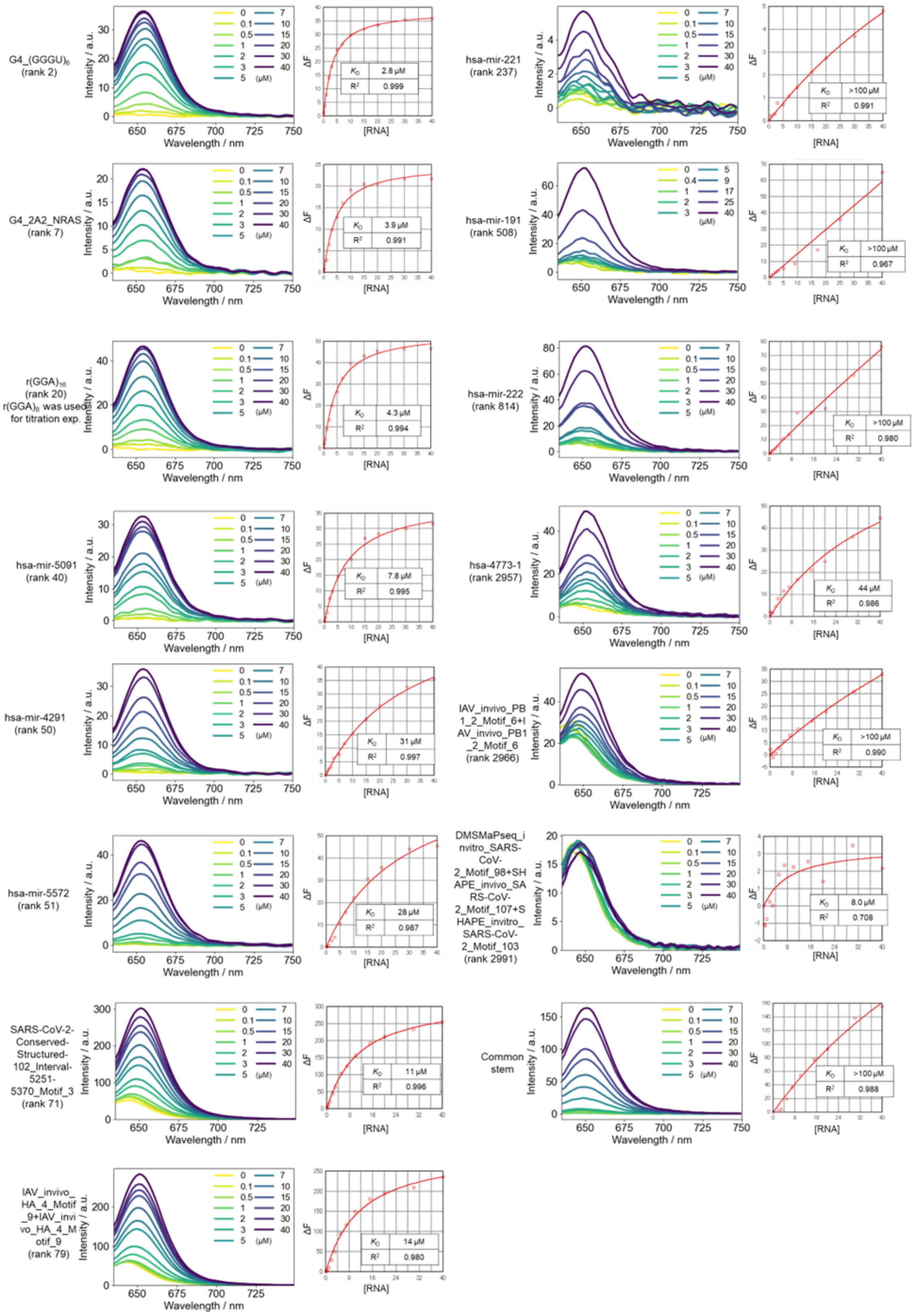

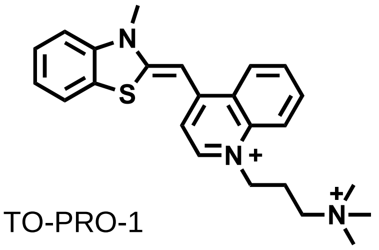

TO-PRO-1

Figure S13. Fluorescence titrations to determine the apparent dissociation constants (K_{Dapp}) of TO-PRO-1. Fluorescence titration spectra were measured using TO-PRO-1 (0.1 μM) upon addition of RNA (0-10 μM) in phosphate buffer (pH 7.0). Conditions: λex: 501 nm, λem: 530 nm. The representative data and K_{Dapp} values are shown.

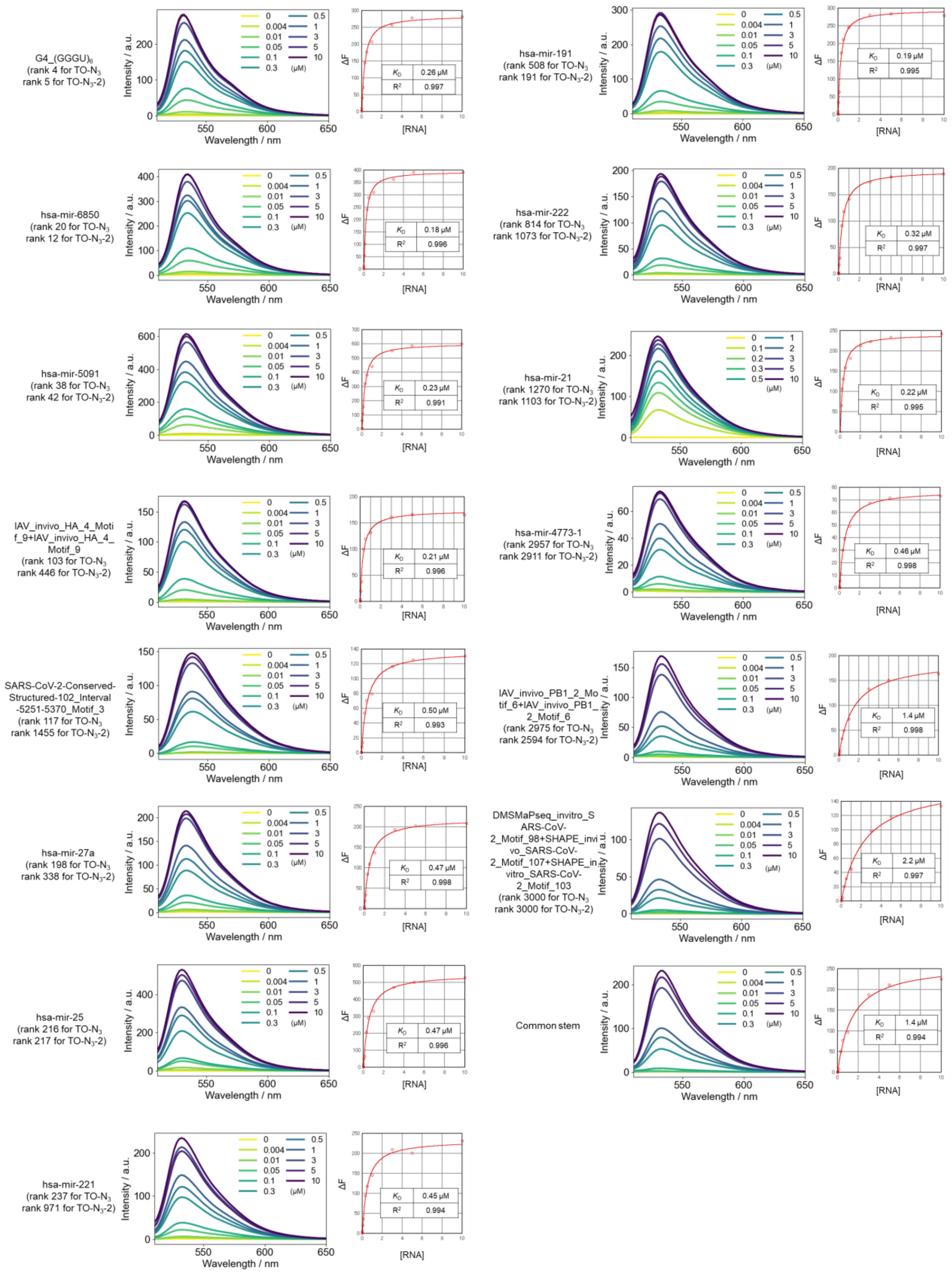

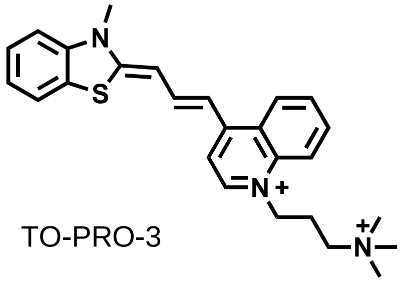

TO-PRO-3

Figure S14. Fluorescence titrations to determine the apparent dissociation constants (K_{Dapp}) of TO-PRO-3. Fluorescence titration spectra were measured using TO-PRO-3 (0.1 μM for G4_r(GGGU)6, hsa-mir-6850 and hsa-mir-5091, or 0.5 μM for the others) upon addition of RNA (0-1 μM for G4_r(GGGU)6, hsa-mir-6850 and hsa-mir-5091, or 0-10 μM for the others) in phosphate buffer (pH 7.0). Conditions: λex: 623 nm, λem: 655 nm. The representative data and K_{Dapp} values are shown.

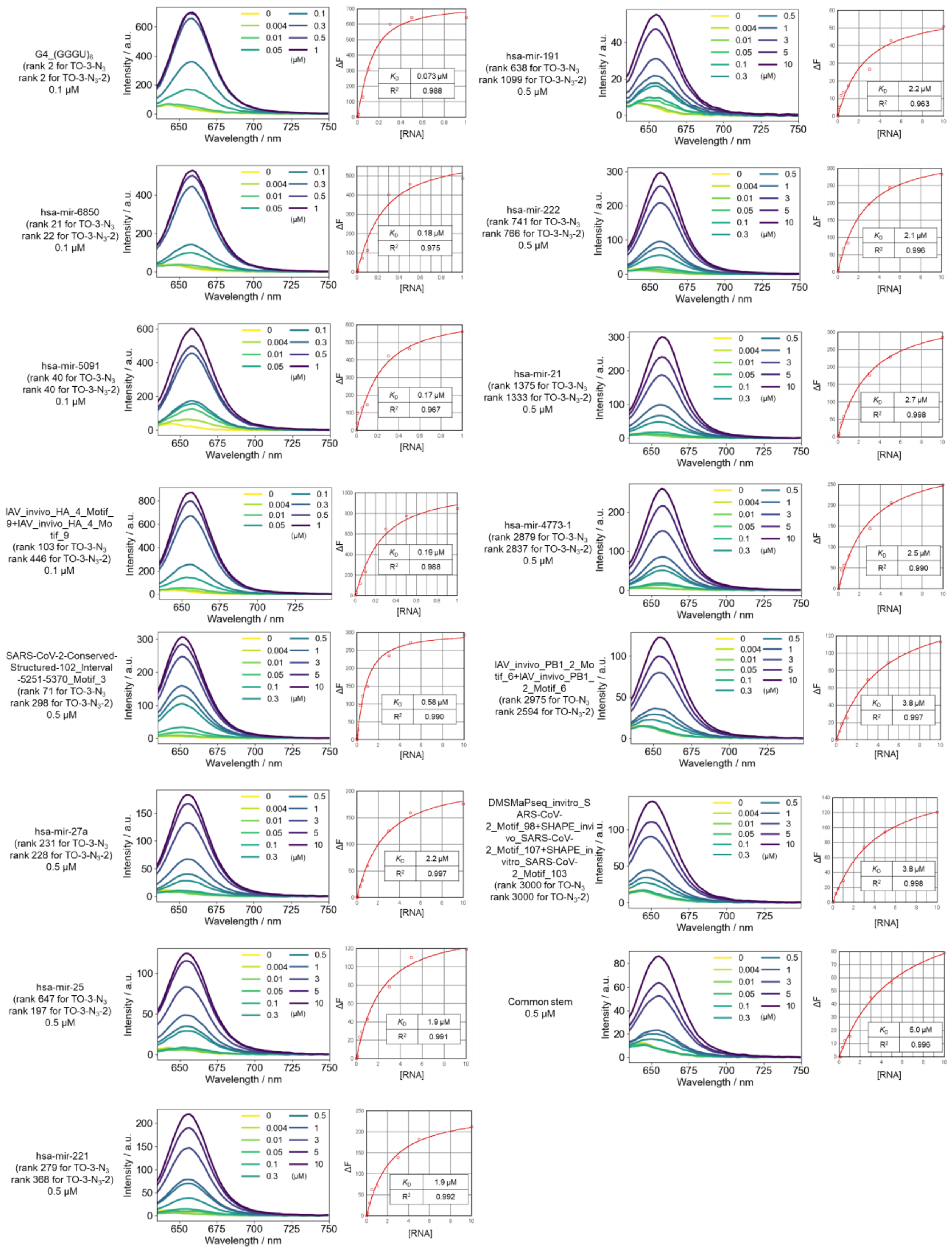

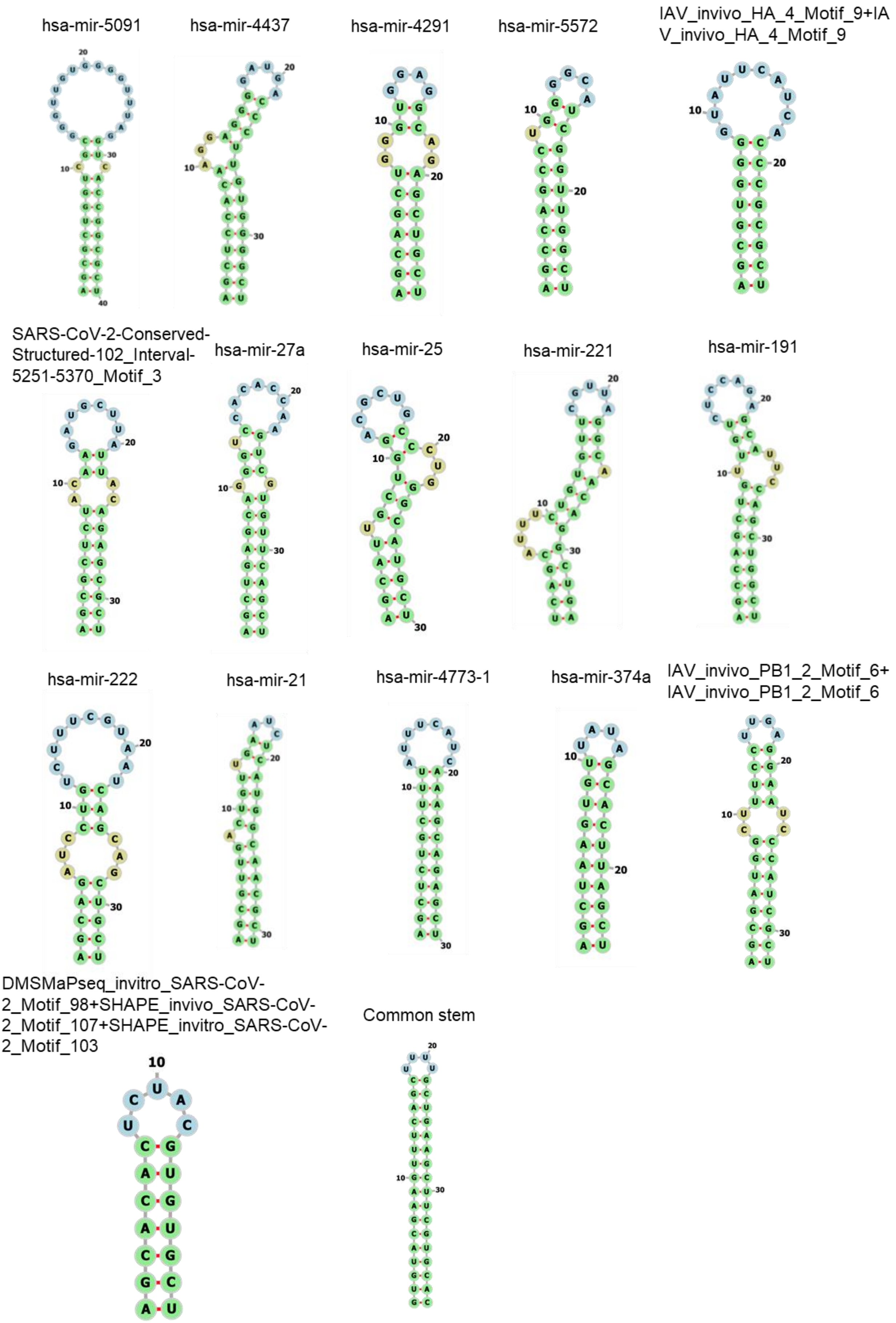

Figure S15. Minimum free energy structures calculated by RNAfold for the assay with TO derivatives.

Table S2. The correlation between Z-scores and K_{Dapp} values for TO-N_3 and TO-3-N_3 RNA binding.

Rank (TO-N_3)	Rank (TO-3-N_3)	Name	Seq.	Z-score (TO-N_3)	Z-score (TO-3-N_3)	TO-N_3's K_D (μM)[a]	TO-3-N_3's K_D (μM)[a]
4	2	G4_r(GGGU)6	GGGUGGGUGGGUGGGUGGGUGGGU	7.43	9.01	1.6±0.3	2.5±0.3
10	7	G4_2A2_NRAS	AAGGGAGGGGCGGGUCGGGAA	7.02	8.72	1.0±0.1	4.9±1
23	20	r(GGA)16	GGAGGAGGAGGAGGAGGAGGAGGAGGAGGAG GAGGAGGAGGAGGAGGA	5.89	7.15	1.8±0.5[b]	6.3±1[b]
38	40	hsa-mir-5091	GCUGGUCGCGGGUUGUGGGGUUUAGGUCACC GGC	3.21	3.15	3.6±0.9	9.5±1
39	35	hsa-mir-4437	UCCACAAGGAGGGGAUGACCCUUGUGGG	3.14	4.12	4.4±1	11±2
-	-	hsa-mir-4437-mut	UCCACA**UCC**AGGGGAUGACCCUUGUGGG	-	-	>40	>40
62	50	hsa-mir-4291	AGCUGGGUGGAGGCAGAGCU	2.34	2.46	11±1	29±4
72	51	hsa-mir-5572	CAGCCUGGGGCAUCGGUUG	2.01	2.41	9.2±2	31±6
103	79	IAV_invivo_HA_4_Motif_9+IAV_invivo_HA_4_Motif_9	GUGGGGUAUUCAUCACCCGC	1.62	1.69	37±0.3	12±0.6
117	71	SARS-CoV-2-Conserved-Structured-102_Interval-5251-5370_Motif_3	GCUCUACAAGAUGCUUAUUACAGAGC	1.52	1.83	n.d.[c]	12±0.5
237	279	hsa-mir-221	AUUUCUGUGUUCGUUAGGCAACAG	1.07	0.79	>40	>40
508	638	hsa-mir-191	CAGCUGUUGUCUCCAGAGCAUUCCAGCUG	0.65	0.37	>40	>40
814	741	hsa-mir-222	AGAUCCUGUCUUUCGUAAUCAGCAGCU	0.34	0.28	>40	>40
2957	2879	hsa-mir-4773-1	UCUGCUUUAUUUCAUCAAAGCAGA	-1.36	-0.92	>40	>40
2975	2966	IAV_invivo_PB1_2_Motif_6+IAV_invivo_PB1_2_Motif_6	GAUGGCUUUCCUUGAGGAAUCCCAUC	-1.41	-1.05	>40	>40
3000	2991	DMSMaPseq_invitro_SARS-CoV-2_Motif_98+SHAPE_invivo_SARS-CoV-2_Motif_107+SHAPE_invitro_SARS-CoV-2_Motif_103	ACACUCUACGUGU	-1.78	-1.14	n.d.[d]	n.d.[d]
-	-	Common stem	GUGUACGAAGUUUCAGCUUUUGCUGAAGCUUC GUGCAC	-	-	>40	>40

a) Data are mean±standard error from three independent experiments.
b) r(GGA)$_8$ was used instead of r(GGA)$_{16}$.
c) K_D could not be determined due to irregular fluorescence change.
d) K_D could not be determined due to negligible fluorescence change.

Table S3. The correlation between Z-scores and K_{Dapp} values for TO-PRO-1 RNA binding.

Rank (TO-N_3)	Rank (TO-N_3-2)	Name	Seq.	Z-score (TO-N_3)	Z-score (TO-N_3-2)	K_D (μM)[a]
4	5	G4_r(GGGU)6	GGGUGGGUGGGUGGGUGGGUGGGU	7.43	5.53	0.23±0.05
20	12	hsa-mir-6850	CGGGGCGGGAGGGGAAGGGACGCCCG	6.39	5.10	0.20±0.03
38	42	hsa-mir-5091	GCUGGUCGCGGGUUGUGGGGUUUAGGUCACCGGC	3.21	2.87	0.17±0.0003
39	232	hsa-mir-4437	UCCACAAGGAGGGGAUGACCCUUGUGGG	3.14	1.37	0.26±0.07
-	-	hsa-mir-4437-mut	UCCACA**UCC**AGGGGAUGACCCUUGUGGG	-	-	0.84±0.07
103	446	IAV_invivo_HA_4_Motif_9+IAV_invivo_HA_4_Motif_9	GUGGGGUAUUCAUCACCCGC	1.62	0.90	0.21±0.005
117	1455	SARS-CoV-2-Conserved-Structured-102_Interval-5251-5370_Motif_3	GCUCUACAAGAUGCUUAUUACAGAGC	1.52	-0.10	0.64±0.1
198	338	hsa-mir-27a	UGAGCAGGGUCCACACCAAGUCGUGUUCA	1.14	1.09	0.45±0.02
216	217	hsa-mir-25	AUUGCUGGACGCUGCCCUGGGCAU	1.12	1.40	0.46±0.08
237	971	hsa-mir-221	AUUUCUGUGUUCGUUAGGCAACAG	1.07	0.32	0.44±0.09
508	191	hsa-mir-191	CAGCUGUUGUCUCCAGAGCAUUCCAGCUG	0.65	1.46	0.20±0.03
814	1073	hsa-mir-222	AGAUCCUGUCUUUCGUAAUCAGCAGCU	0.34	0.23	0.40±0.06
1270	1103	hsa-mir-21	GUUGACUGUUGAAUCUCAUGGCAAC	0.00	0.20	0.21±0.008
2966	2989	hsa-mir-374a	UAAGUGUUAUAGCACUUA	-1.38	-1.79	1.1±0.17
2957	2911	hsa-mir-4773-1	UCUGCUUUAUUUCAUCAAAGCAGA	-1.36	-1.41	0.43±0.01
2975	2594	IAV_invivo_PB1_2_Motif_6+IAV_invivo_PB1_2_Motif_6	GAUGGCUUUCCUUGAGGAAUCCCAUC	-1.41	-0.99	1.4±0.04
3000	3000	DMSMaPseq_invitro_SARS-CoV-2_Motif_98+SHAPE_invivo_SARS-CoV-2_Motif_107+SHAPE_invitro_SARS-CoV-2_Motif_103	ACACUCUACGUGU	-1.78	-1.93	2.2±0.04
-	-	Common stem	GUGUACGAAGUUUCAGCUUUUGCUGAAGCUUCGUG CAC	-	-	1.1±0.08

a) Data are mean±standard error from three independent experiments.

Table S4. The correlation between Z-scores and K_{Dapp} values for TO-PRO-3 RNA binding.

Rank (TO-3-N_3)	Rank (TO-3-N_3-2)	Name	Seq.	Z-score (TO3-N_3)	Z-score (TO3-N_3-2)	K_D (μM)[a]
2	4	G4_r(GGGU)6	GGGUGGGUGGGUGGGUGGGUGGGU	9.01	10.96	0.092±0.02
21	21	hsa-mir-6850	CGGGGCGGGAGGGGAAGGGACGCCCG	6.93	7.68	0.19±0.01
40	39	hsa-mir-5091	GCUGGUCGCGGGUUGUGGGGUUUAGGUCACCGGC	3.15	2.61	0.14±0.01
35	35	hsa-mir-4437	UCCACAAGGAGGGGAUGACCCUUGUGGG	4.12	3.03	0.28±0.05
-	-	hsa-mir-4437-mut	UCCACA**UCC**AGGGGAUGACCCUUGUGGG	-	-	1.7±0.03
79	126	IAV_invivo_HA_4_Motif_9+IAV_invivo_HA_4_Motif_9	GUGGGGUAUUCAUCACCCGC	1.69	0.60	0.18±0.02
71	213	SARS-CoV-2-Conserved-Structured-102_Interval-5251-5370_Motif_3	GCUCUACAAGAUGCUUAUUACAGAGC	1.83	0.42	0.98±0.3
231	324	hsa-mir-27a	UGAGCAGGGUCCACACCAAGUCGUGUUCA	0.87	0.30	2.0±0.08
647	299	hsa-mir-25	AUUGCUGGACGCUGCCCUGGGCAU	0.36	0.32	1.5±0.3
279	593	hsa-mir-221	AUUUCUGUGUUCGUUAGGCAACAG	0.79	0.13	1.2±0.3
638	959	hsa-mir-191	CAGCUGUUGUCUCCAGAGCAUUCCAGCUG	0.37	-0.01	2.2±0.2
741	1539	hsa-mir-222	AGAUCCUGUCUUUCGUAAUCAGCAGCU	0.28	-0.15	1.5±0.3
1375	1792	hsa-mir-21	GUUGACUGUUGAAUCUCAUGGCAAC	-0.11	-0.21	1.9±0.3
2867	2040	hsa-mir-374a	UAAGUGUUAUAGCACUUA	-0.90	-0.27	6.1±1.4
2879	2938	hsa-mir-4773-1	UCUGCUUUAUUUCAUCAAAGCAGA	-0.92	-0.61	2.4±0.3
2966	2797	IAV_invivo_PB1_2_Motif_6+IAV_invivo_PB1_2_Motif_6	GAUGGCUUUCCUUGAGGAAUCCCAUC	-1.05	-0.49	3.7±0.2
2991	2770	DMSMaPseq_invitro_SARS-CoV-2_Motif_98+SHAPE_invivo_SARS-CoV-2_Motif_107+SHAPE_invitro_SARS-CoV-2_Motif_103	ACACUCUACGUGU	-1.14	-0.48	4.3±0.6
-	-	Common stem	GUGUACGAAGUUUCAGCUUUUGCUGAAGCUUCGUGCAC	-	-	4.7±0.6

a) Data are mean±standard error from three independent experiments.

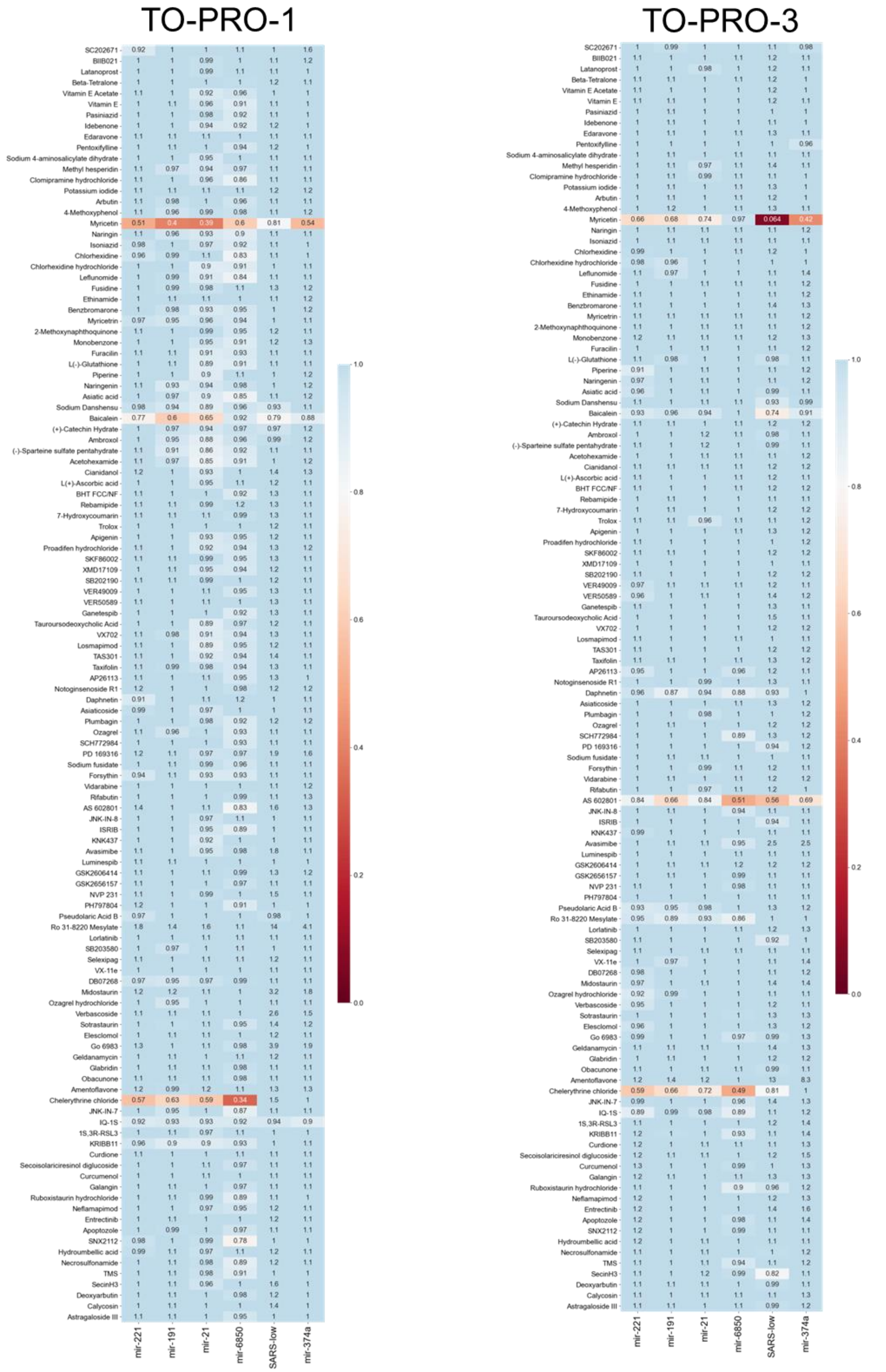

Figure S16. Normalized fluorescences in FID assay using TO-PRO-1 (left) and TO-PRO-3 (right). The data are mean from two independent experiments.

baicalein

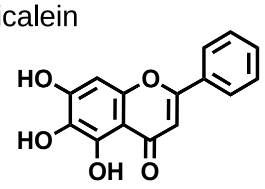

Figure S17. Fluorescence titrations to determine the apparent dissociation constants (K_{Dapp}) of baicalein. Fluorescence titration spectra were measured using baicalein (5 or 10 µM) upon addition of RNA (0-40 µM) in phosphate buffer (pH 7.0). Conditions: λex: 315 nm, λem: 404 nm. The representative data and K_{Dapp} values are shown.

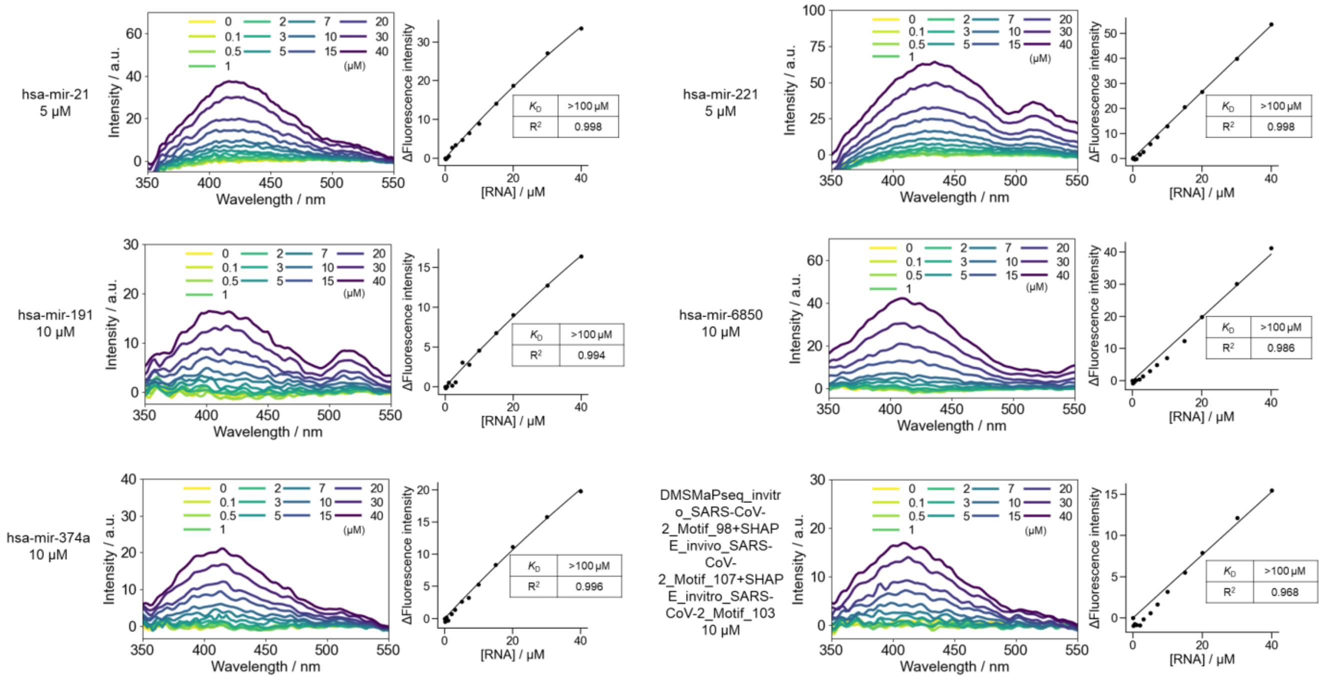

myricetin

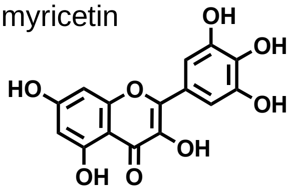

Figure S18. Fluorescence titrations to determine the apparent dissociation constants (K_{Dapp}) of myricetin. Fluorescence titration spectra were measured using myricetin (1 or 5 µM) upon addition of RNA (0-40 µM) in phosphate buffer (pH 7.0). Conditions: λex: 376 nm, λem: 542 nm. The representative data and K_{Dapp} values are shown.

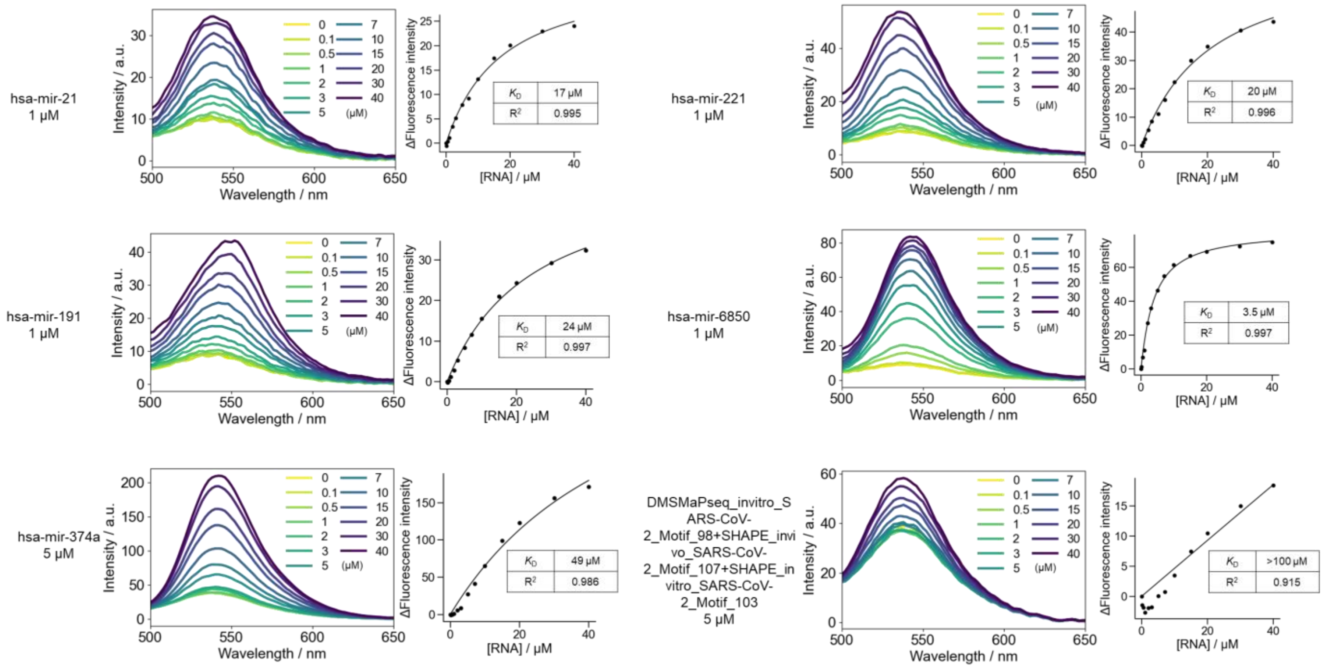

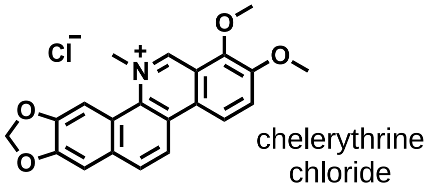

Figure S19. Fluorescence titrations to determine the apparent dissociation constants (K_{Dapp}) of chelerythrine chloride. Fluorescence titration spectra were measured using chelerythrine (1 µM) upon addition of RNA (0-40 µM) in phosphate buffer (pH 7.0). Conditions: λex: 320 nm, λem: 414 nm. The representative data and K_{Dapp} values are shown.

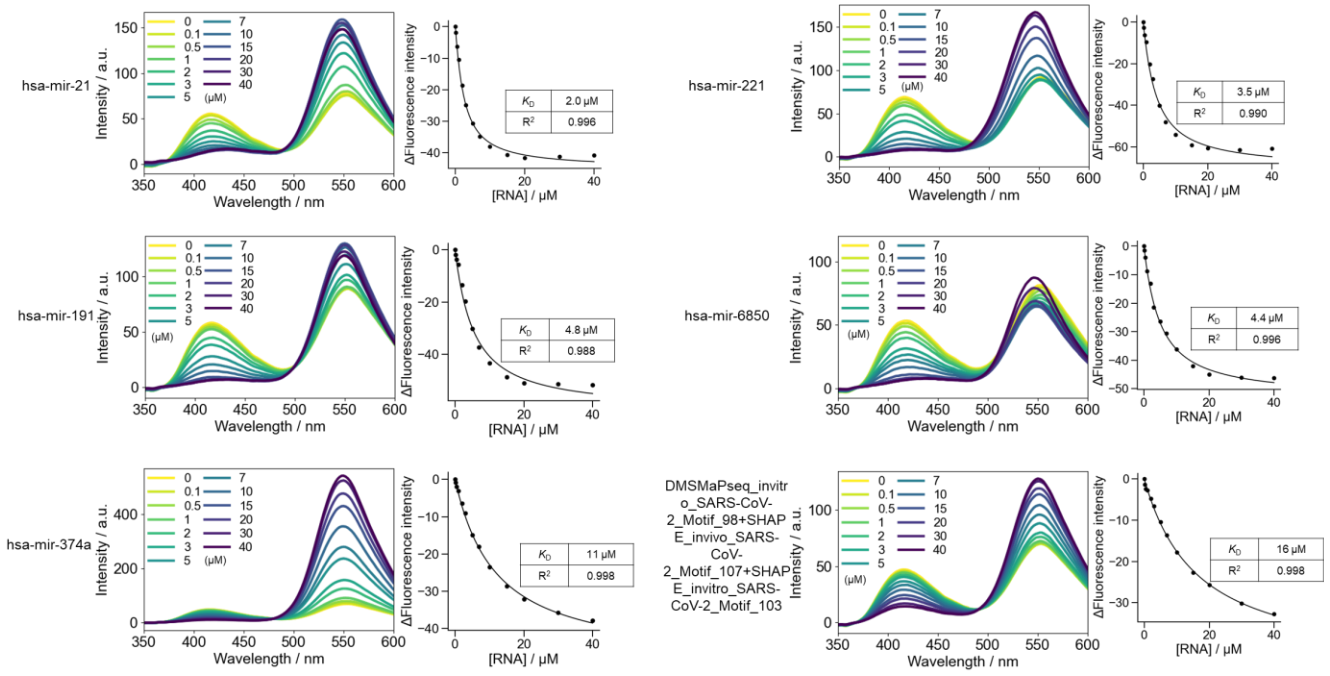

A

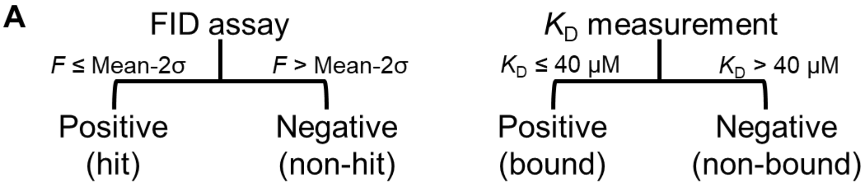

True positive (TP): the number of hit and bound

False positive (FP): the number of hit and non-bound

Precision = TP/(TP+FP)

B

precision	221	191	21	6850	SARS	374a
TO-PRO-1	0.67	0.67	0.67	1	0	0
TO-PRO-3	0.67	1	1	1	0	0

Figure S20. The precisions of FID assay. (A) Definition of true positive, false positive and precision. *F* denotes fluorescence intensity in FID assay and σ denotes standard division. (B) The precisions of FID assay using TO-PRO-1 and TO-PRO-3 across the investigated RNAs.

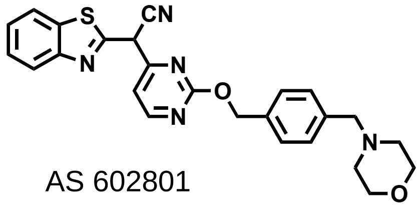

AS 602801

Figure S21. Fluorescence titrations to determine the apparent dissociation constants (K_{Dapp}) of AS 602801. Fluorescence titration spectra were measured using AS 602801 (1 µM) upon addition of RNA (0-40 µM) in phosphate buffer (pH 7.0). Conditions: λex: 372 nm, λem: 432 nm. The representative data and K_{Dapp} values are shown.

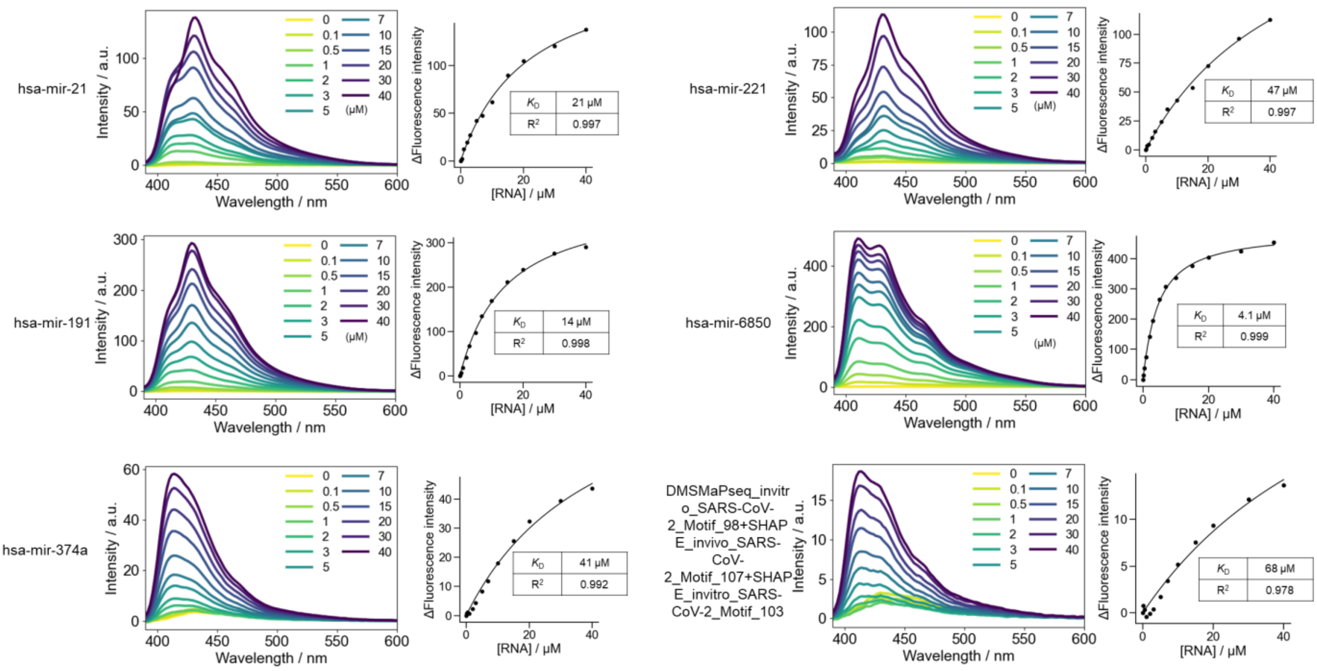

NMR Data

G-clamp-N_3, ^{1}H NMR (400 MHz, DMSO-d_6)

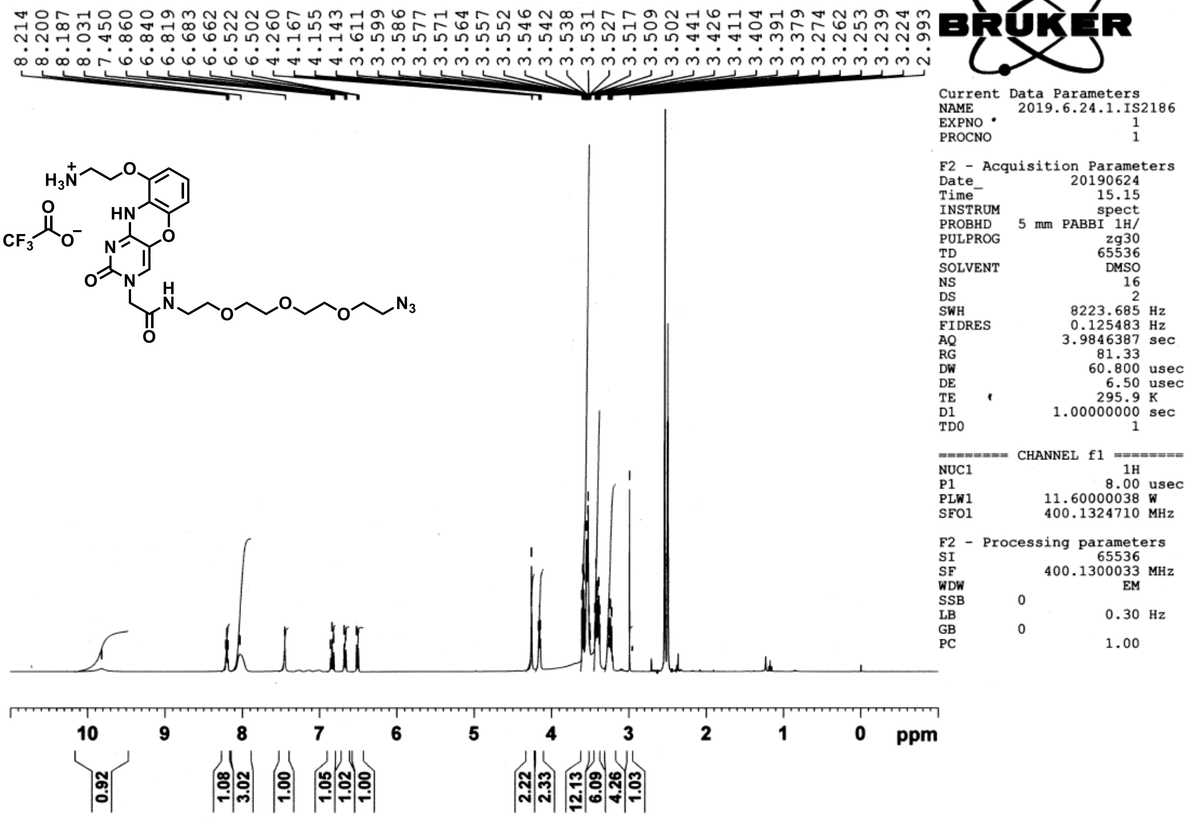

G-clamp-N_3, ^{13}C NMR (151 MHz, DMSO-d_6)

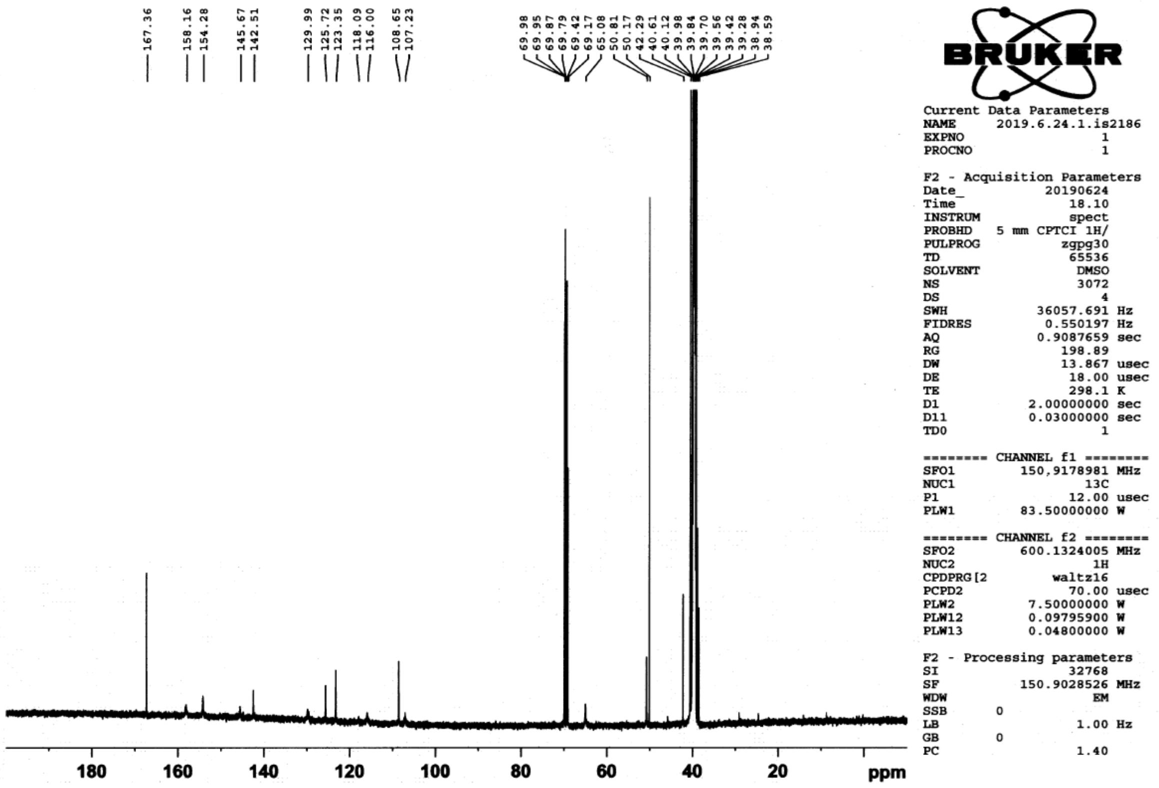

Compound **5**, ^{1}H-NMR (400 MHz, DMSO-d_6)

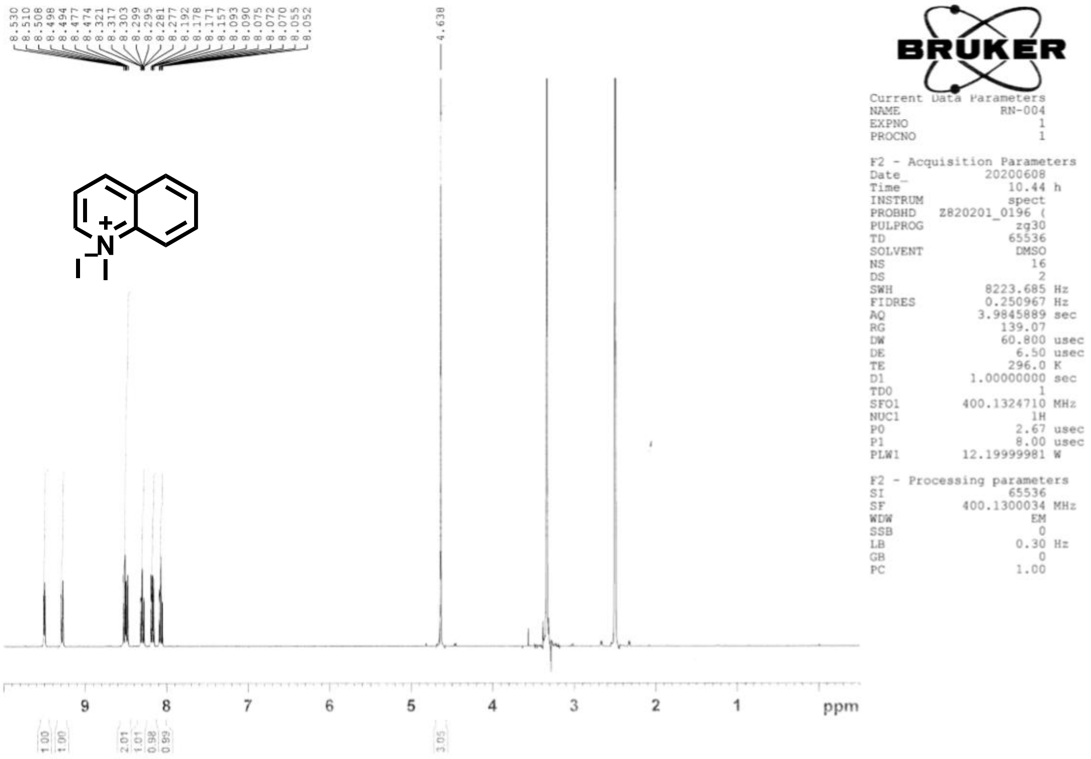

Compound **6**, ^{1}H-NMR (400 MHz, DMSO-d_6)

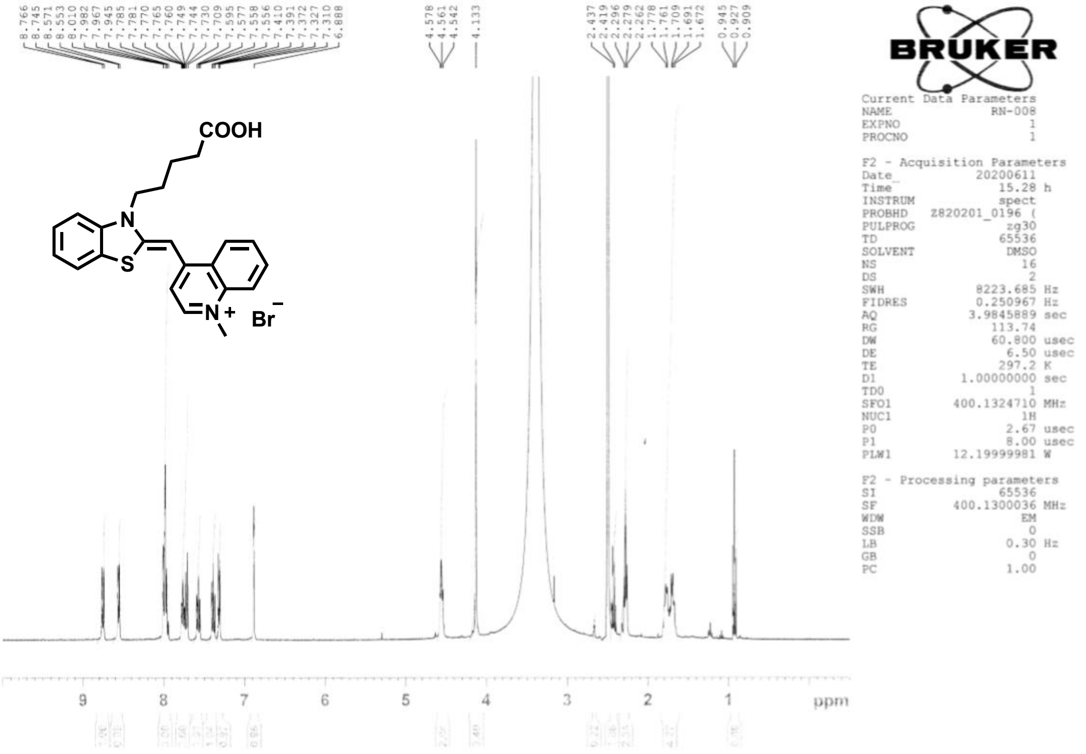

TO-N_3, ^{1}H-NMR (600 MHz, DMSO-d_6)

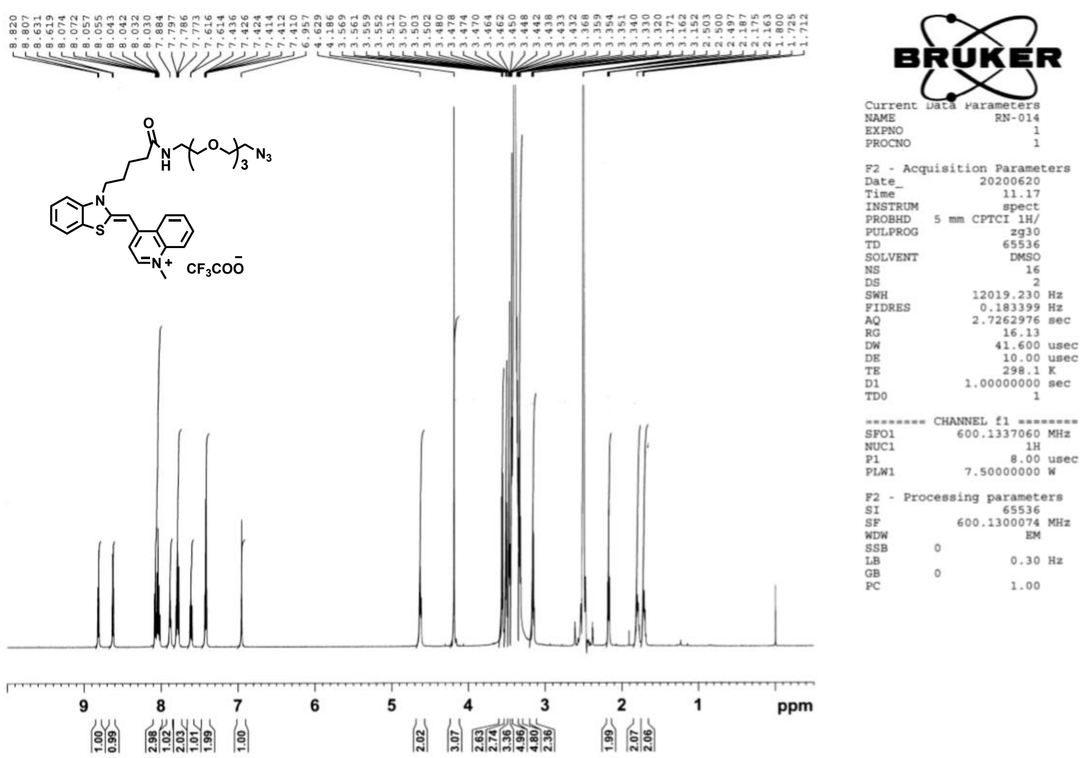

TO-N_3, ^{13}C-NMR (151 MHz, DMSO-d_6)

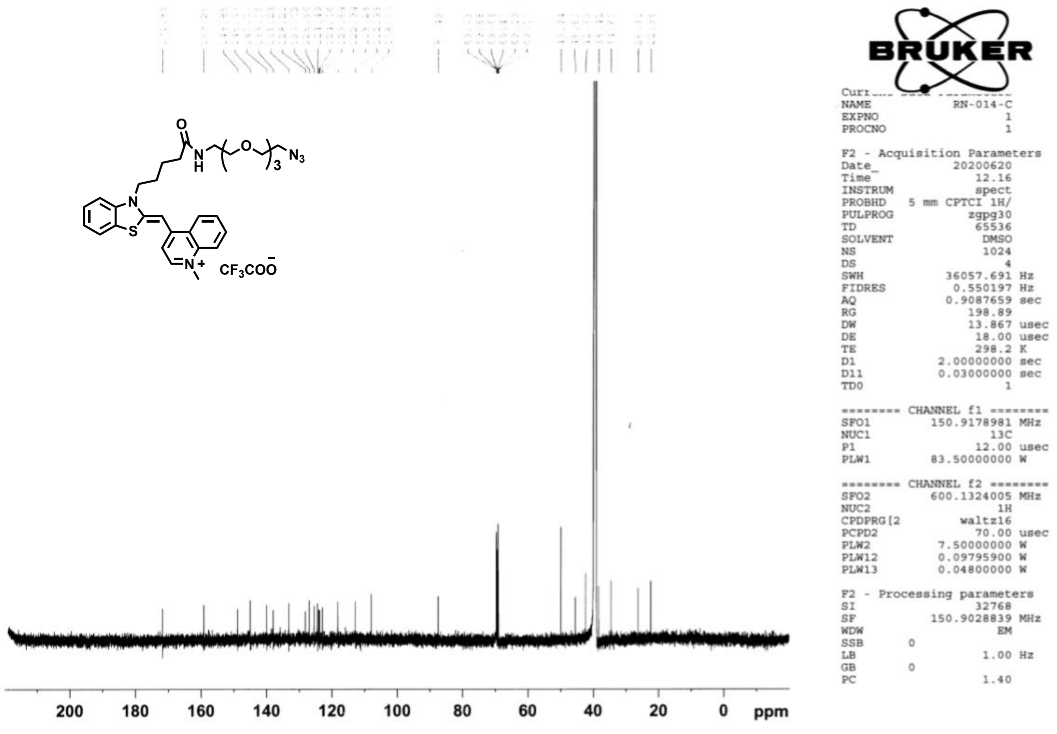

Compound **10**, ^{1}H-NMR (400 MHz, DMSO-d_6)

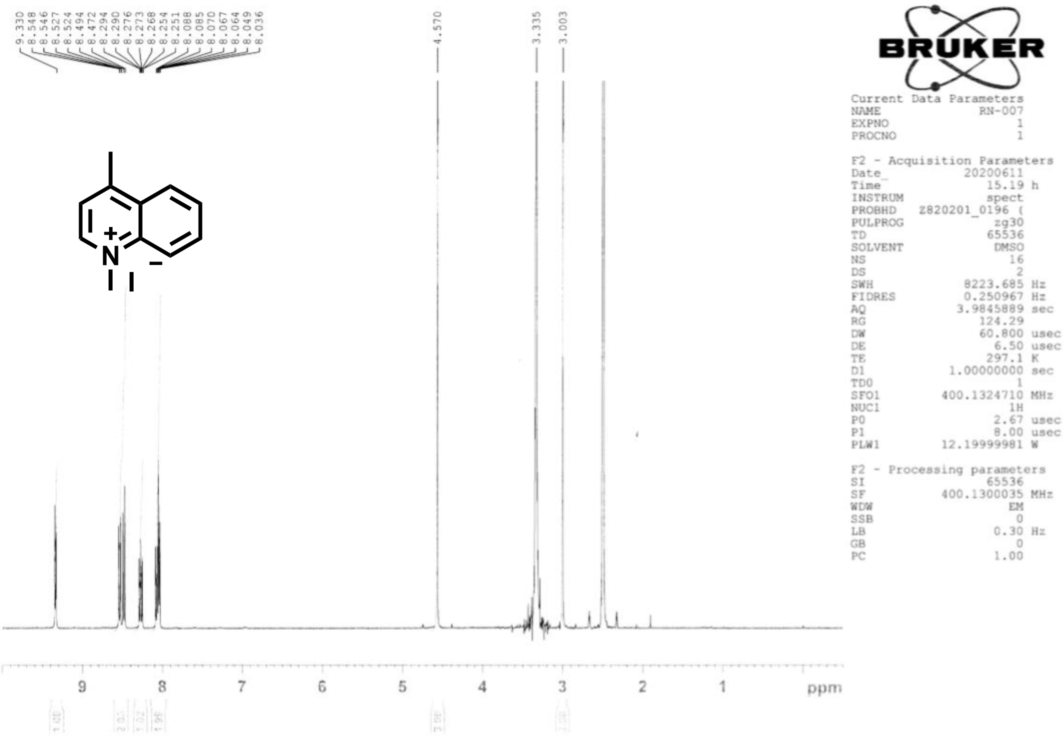

Compound **11**, ^{1}H-NMR (400 MHz, DMSO-d_6)

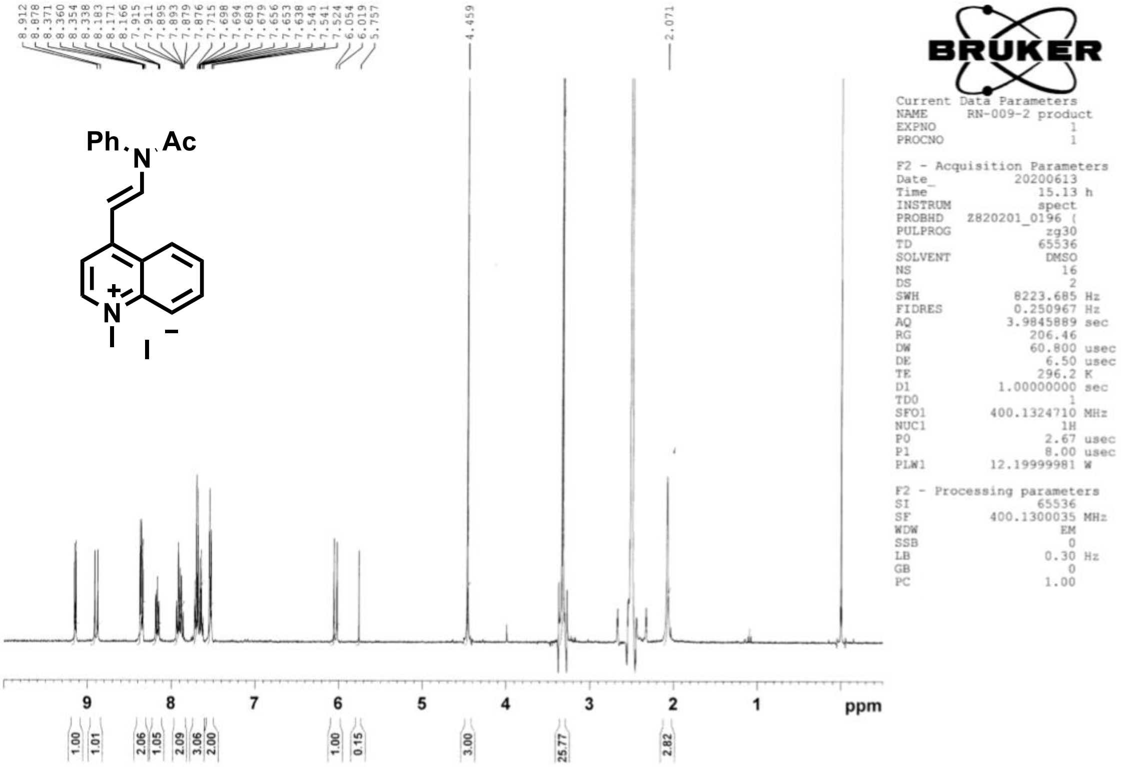

Compound **12**, ^{1}H-NMR (400 MHz, DMSO-d_6)

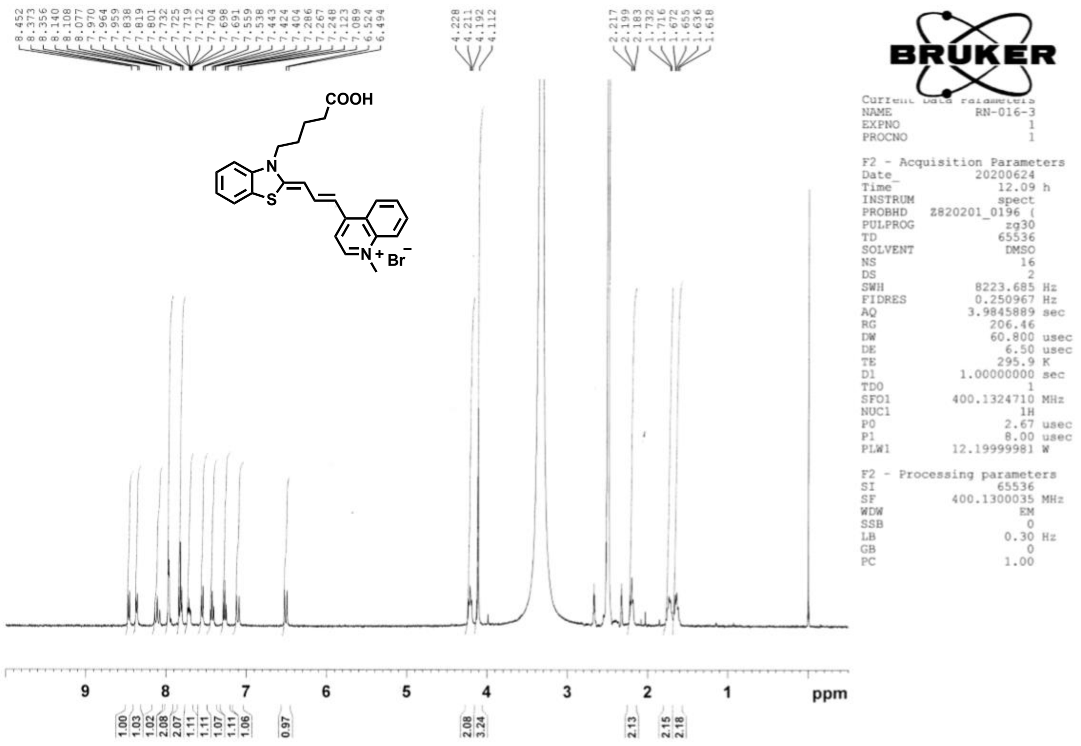

TO-3-N$_3$, ^{1}H-NMR (600 MHz, DMSO-d_6)

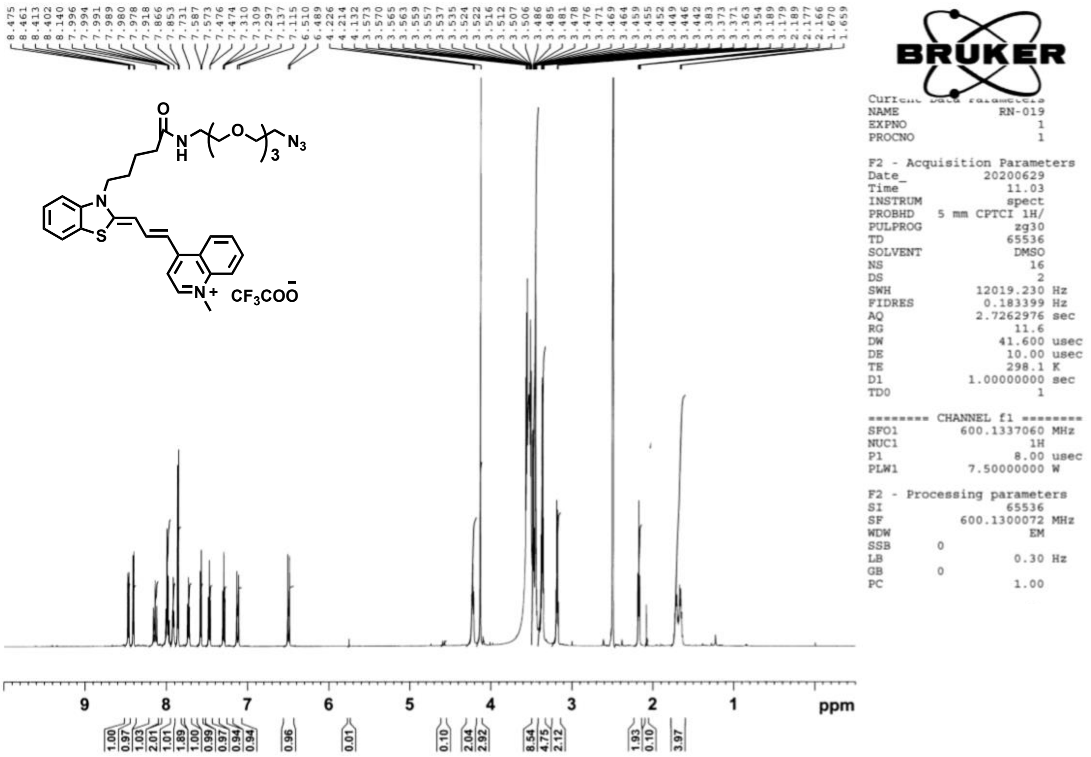

TO-3-N$_3$, ^{13}C-NMR (151 MHz, DMSO-d_6)

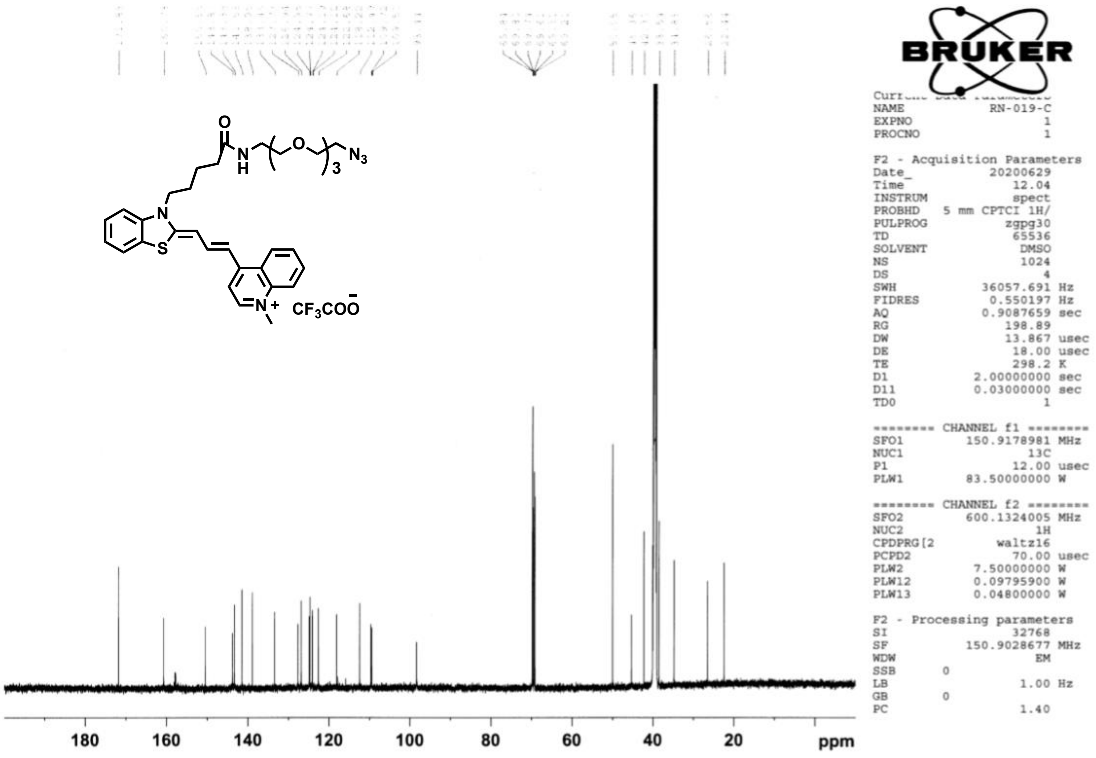

Compound **15**, ^{1}H-NMR (500 MHz, $CDCl_3$)

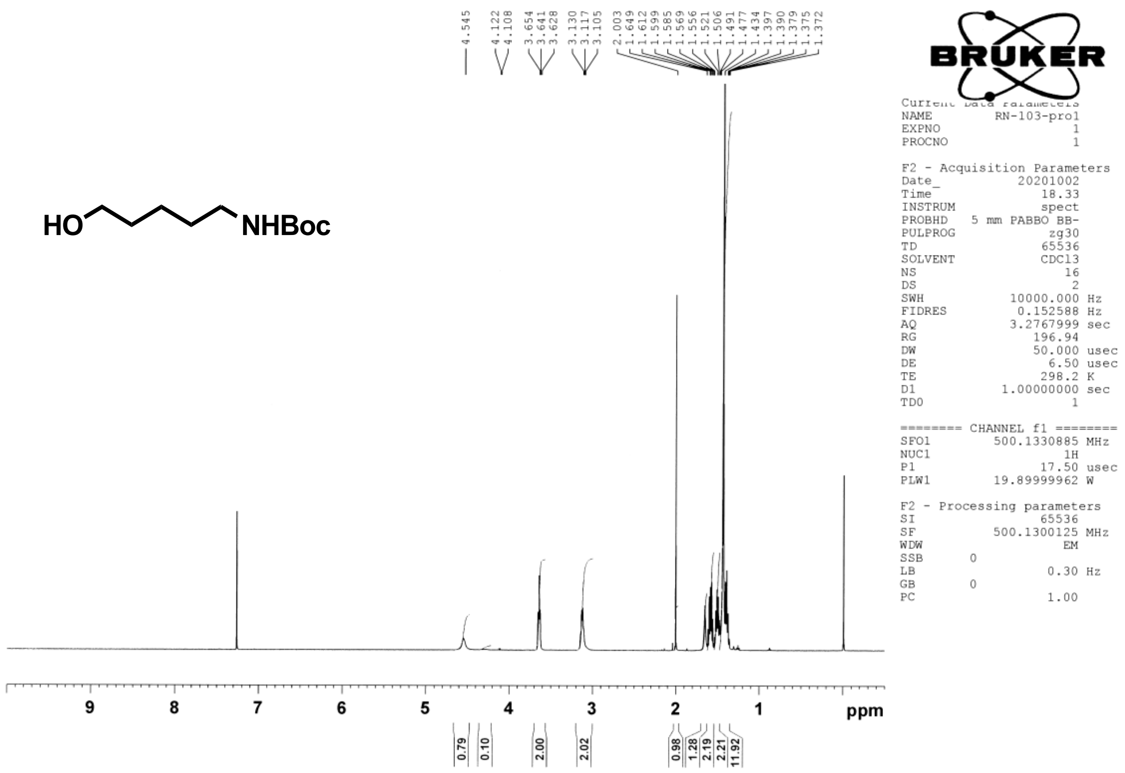

Compound **16**, ^{1}H-NMR (400 MHz, $CDCl_3$)

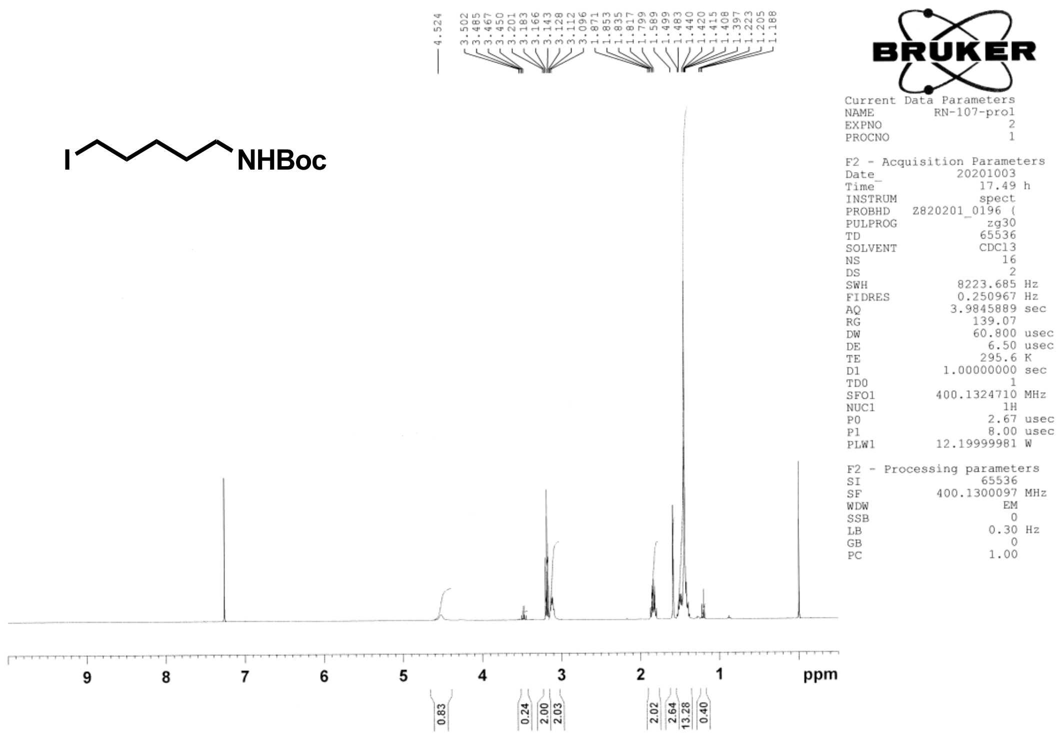

Compound **17**, ^{1}H-NMR (500 MHz,DMSO-d_6)

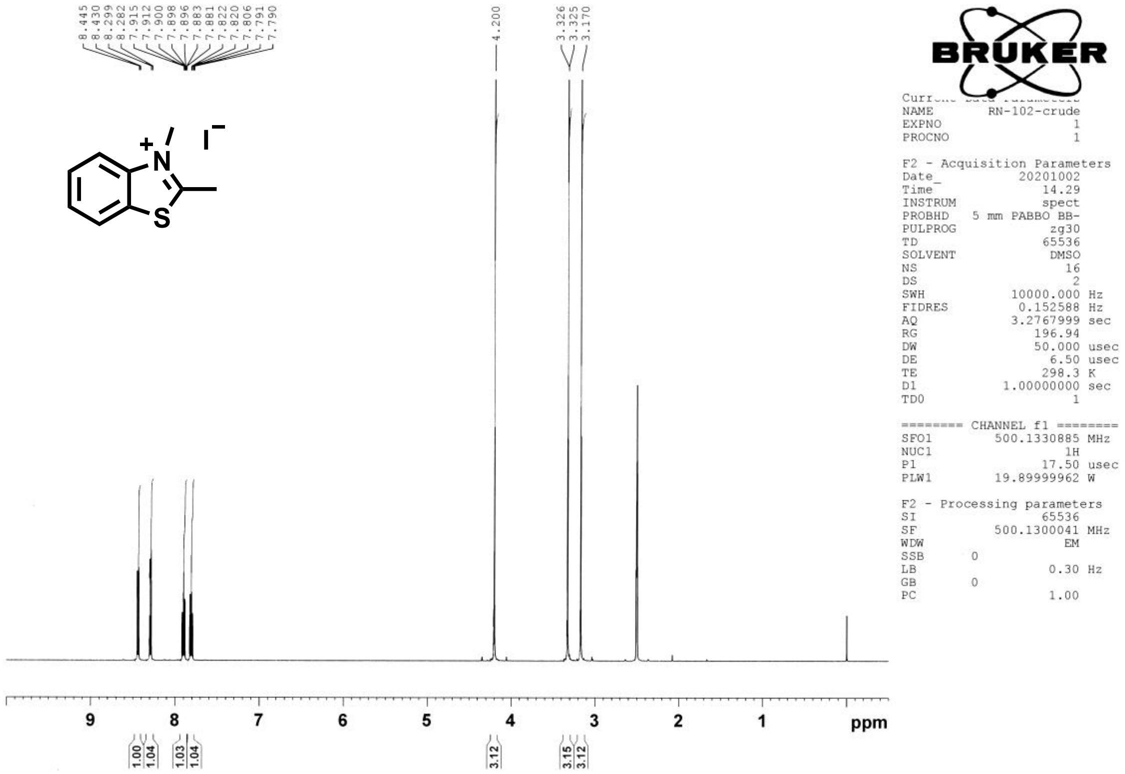

TO-N_3-2, ^{1}H-NMR (600 MHz, DMSO-d_6)

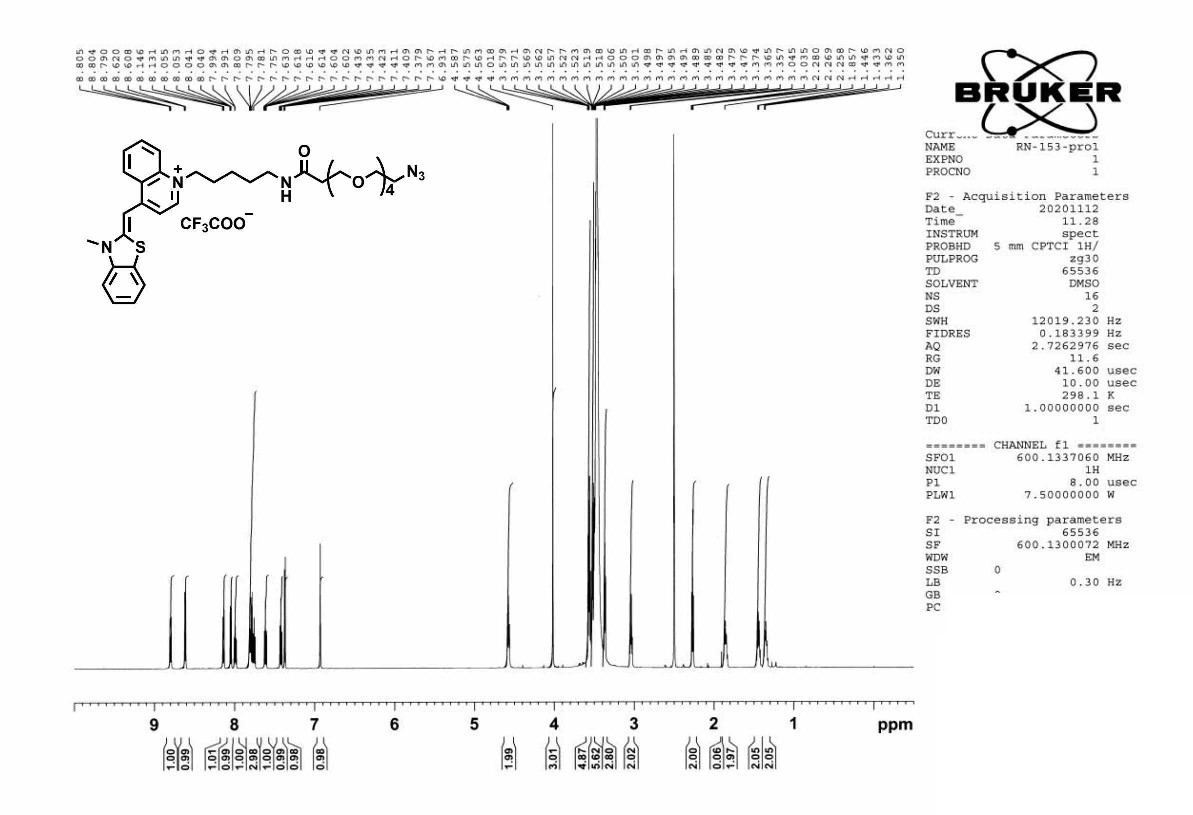

TO-N_3-2, ^{13}C-NMR (151 MHz, DMSO-d_6)

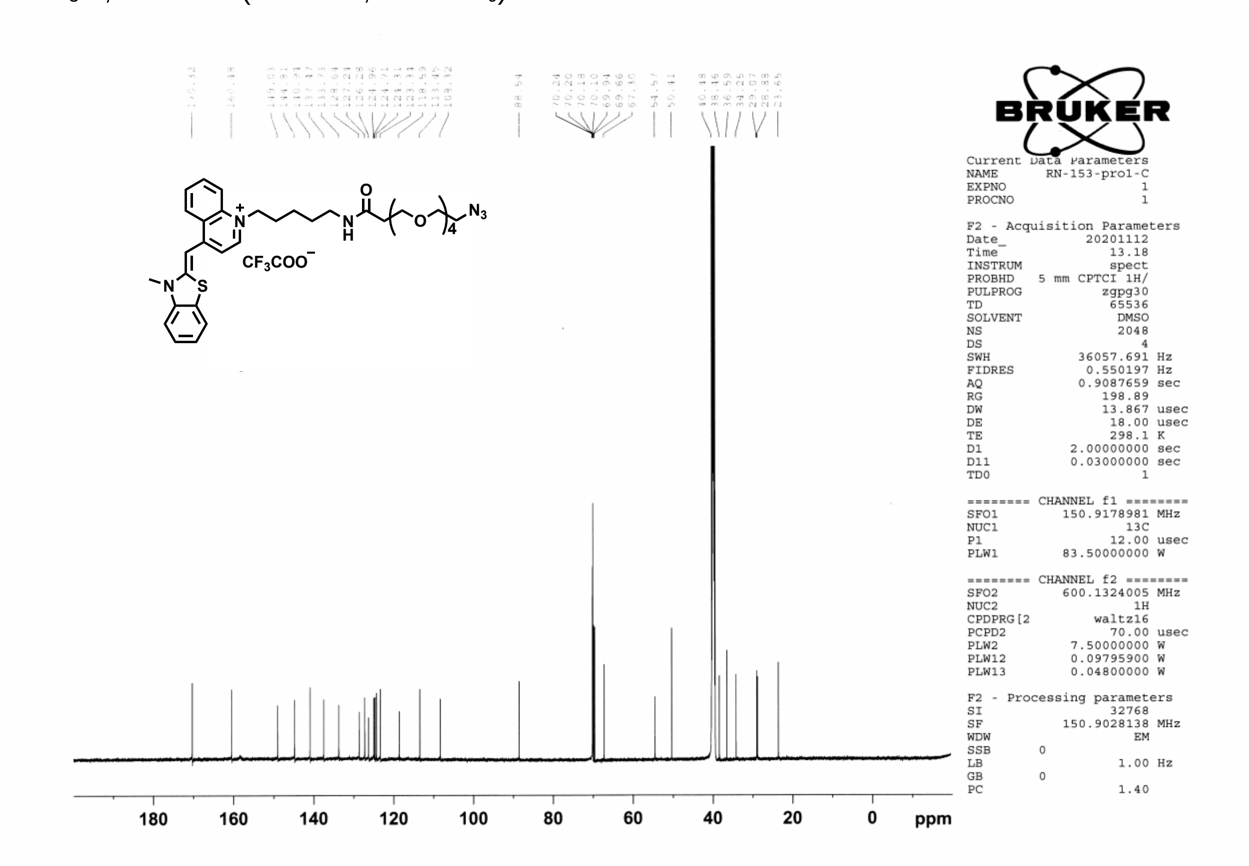

Compound **23**, ^{1}H-NMR (400 MHz, DMSO-d_6)

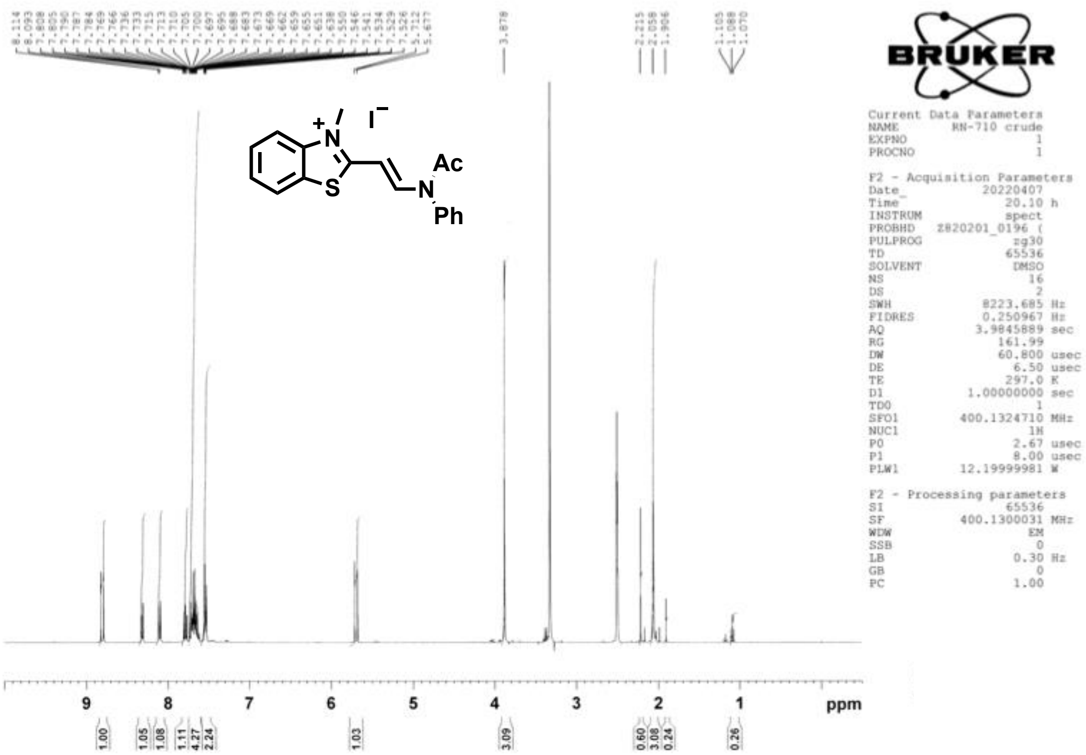

Compound **25**, ^{1}H-NMR (600 MHz, DMSO-d_6)

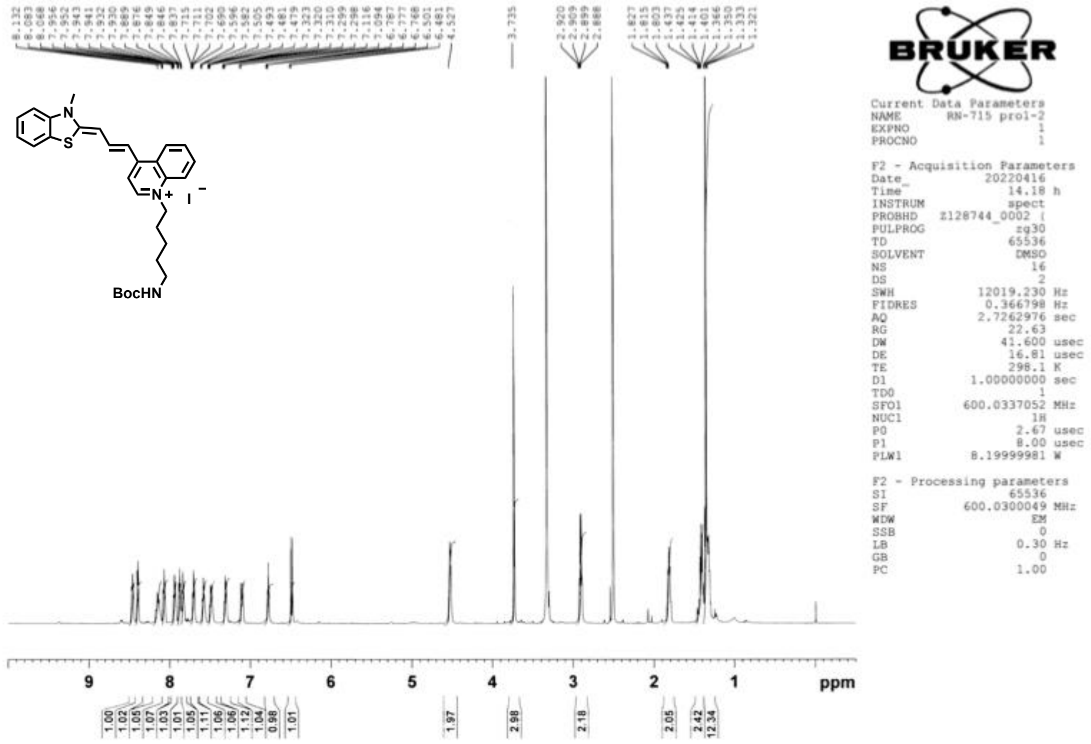

Compound **25**, ^{13}C-NMR (151 MHz, DMSO-d_6)

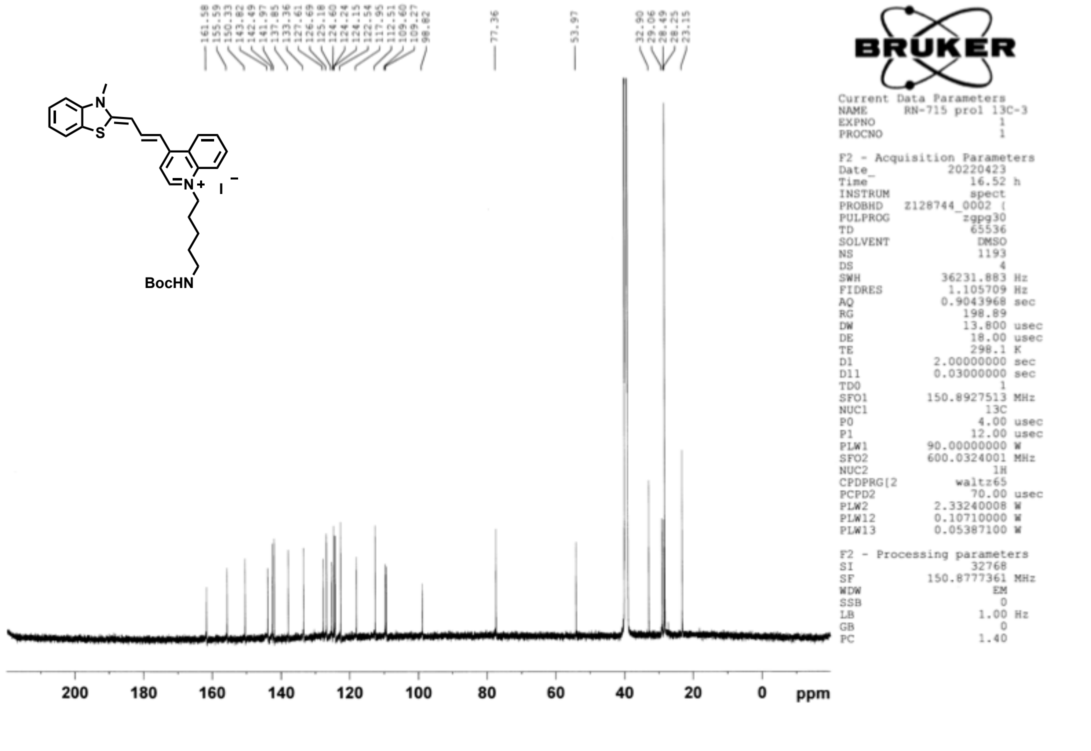

TO-3-N_3-2, ^{1}H-NMR (600 MHz, DMSO-d_6)

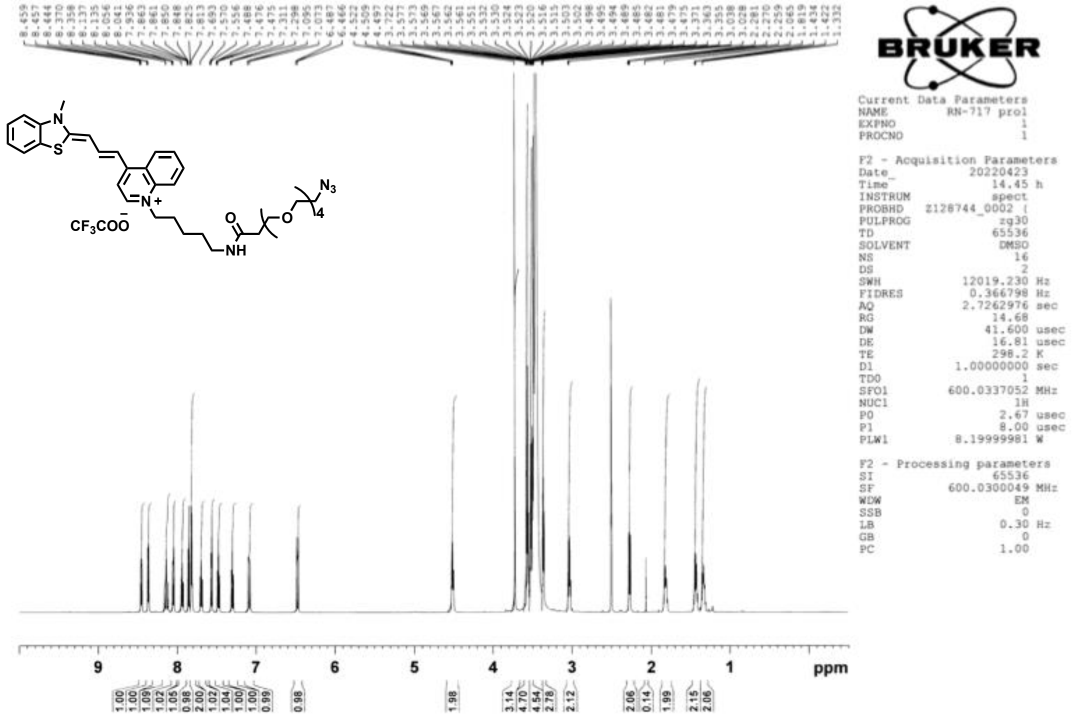

TO-3-N_3-2, ^{13}C-NMR (151 MHz, DMSO-d_6)

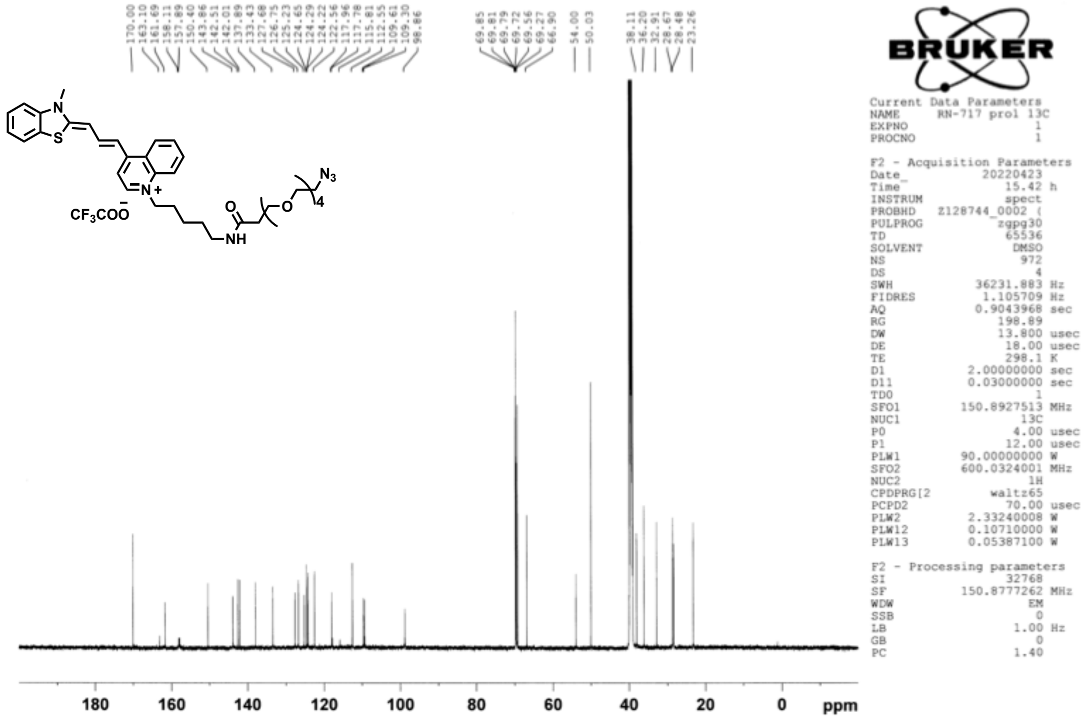

Supplement: Supplementary file 2 — Supplemental Information [file 42004_2024_1181_MOESM2_ESM.pdf]
