# Supplementary material for: Large-scale analysis of small molecule-RNA interactions using multiplexed RNA structure libraries
Source: Commun Chem. 2024 May 1;7:98. doi: 10.1038/s42004-024-01181-8 (PMC11865577; doi:10.1038/s42004-024-01181-8)
Supplement: Supplementary file 3 — Description of Additional Supplementary Files [file 42004_2024_1181_MOESM3_ESM.pdf]

Description of Additional Supplementary Files

File name: Supplementary Data 1

Description: Ranking list (G-clamp)

File name: Supplementary Data 2

Description: Ranking list (TO-N_3)

File name: Supplementary Data 3

Description: Ranking list (TO-N_3-2)

File name: Supplementary Data 4

Description: Ranking list (TO-3-N_3)

File name: Supplementary Data 5

Description: Ranking list (TO-3-N_3-2)

File name: Supplementary Data 6

Description: Structures of chemical library for FID assay

File name: Supplementary Data 7

Description: Supplementary list of FID assay results using TO-PRO-1

File name: Supplementary Data 8

Description: Supplementary list of FID assay results using TO-PRO-3
