# Supplementary figures and images for: Large-scale analysis of small molecule-RNA interactions using multiplexed RNA structure libraries
Source: Commun Chem. 2024 May 1;7:98. doi: 10.1038/s42004-024-01181-8 (PMC11865577; doi:10.1038/s42004-024-01181-8)

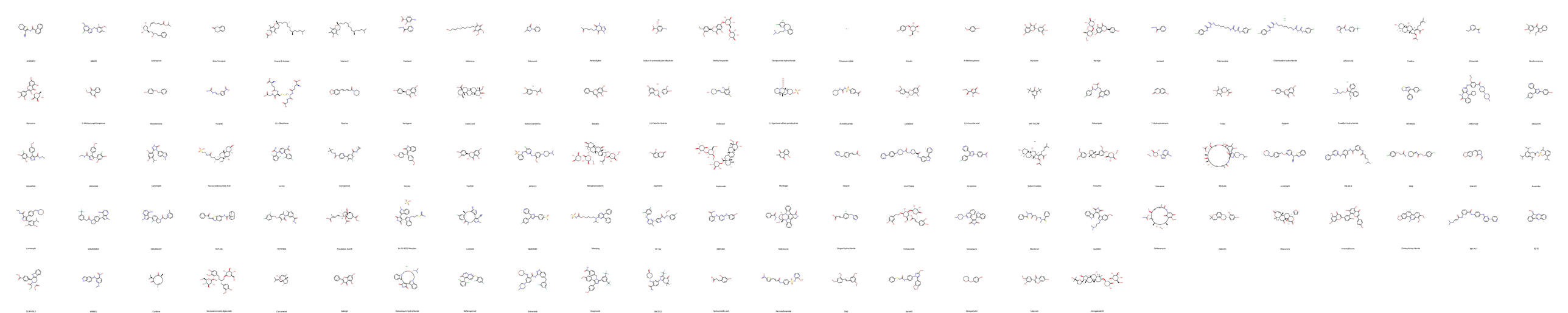

Supplement: Supplementary file 9 — Supplementary Data 6 [file 42004_2024_1181_MOESM9_ESM.jpg]
